# Transcription-dependent confined diffusion of enzymes within subcellular spaces of the bacterial cytoplasm

**DOI:** 10.1186/s12915-021-01083-4

**Published:** 2021-09-02

**Authors:** Daniel A. O. Rotter, Christoph Heger, Luis M. Oviedo-Bocanegra, Peter L. Graumann

**Affiliations:** 1 SYNMIKRO, LOEWE Center for Synthetic Microbiology, Marburg, Germany; 2 Department of Chemistry, Philipps-Universität Marburg, Marburg, Germany

**Keywords:** Bacterial nanocompartments, Riboflavin biosynthesis, Heavy riboflavin synthase, Lumazine synthase, Single particle tracking, Brownian motion, Anomalous diffusion, transcription, *Bacillus subtilis*

## Abstract

**Background:**

Knowledge on the localization and mobility of enzymes inside bacterial cells is scarce, but important for understanding spatial regulation of metabolism. The four central enzymes (Rib enzymes) of the riboflavin (RF) biosynthesis pathway in the Gram positive model bacterium *Bacillus subtilis* have been studied extensively in vitro, especially the heavy RF synthase, a large protein complex with a capsid structure formed by RibH and an encapsulated RibE homotrimer, which mediates substrate-channeling. However, little is known about the behavior and mobility of these enzymes in vivo.

**Results:**

We have investigated the localization and diffusion of the Rib enzymes in the cytoplasm of *B. subtilis*. By characterizing the diffusion of Rib enzymes in live cells using single particle tracking (SPT) we provide evidence for confined diffusion at the cell poles and otherwise Brownian motion. A majority of RibH particles showed clear nucleoid occlusion and a high degree of confined motion, which is largely abolished after treatment with Rifampicin, revealing that confinement is dependent on active transcription. Contrarily, RibE is mostly diffusive within the cell, showing only 14% encapsulation by RibH nanocompartments. By localizing different diffusive populations within single cells, we find that fast diffusion occurs mostly across the nucleoids located in the cell centers, while the slower, confined subdiffusion occurs at the crowded cell poles.

**Conclusions:**

Our results provide evidence for locally different motion of active enzymes within the bacterial cytoplasm, setting up metabolic compartmentalization mostly at the poles of cells.

**Supplementary Information:**

The online version contains supplementary material available at 10.1186/s12915-021-01083-4.

## Background

Eukaryotic cells use cell compartments for spatial separation and regulation of their metabolism, while it has long been thought that the bacterial cytoplasm does not allow for such spatial regulation because of rapid mixing of its components by passive diffusion (Brownian motion). Since the advent of bacterial cell biology, we know that bacteria are not simple “bags of enzymes,” but show a high degree of intracellular organization in time and space [1]. Pioneering work has shown that the bacterial cytoplasm can even have glass-like properties that are dependent on the size of the particle studied, as well as on the metabolic state of the cell [2], but it has been unclear if the non-compartmentalized bacterial cell offers subcellular regions where metabolic enzymes might preferably localize. The large central bacterial nucleoid has been considered as a subcellular space enriched in DNA and in proteins/enzymes with DNA binding capabilities, and thus reduced in cytoplasmic proteins. Potentially, nucleoids could serve as a physical barrier setting up concentration gradients between cell poles and nucleoid(s) [3]. Furthermore, aqueous phase separation has been discussed as the overriding physical principle governing micro-compartmentation of constituents in the bacterial cytoplasm as a consequence of molecular crowding [4].

Based on the idea that nucleoid central occupation could exclude space available for cytoplasmic proteins, spaces at the cell poles and at future division sites of cells between segregated nucleoids could comprise localized metabolism. These regions are likely to be very dynamic in their composition due to colocalized active translation and do thus also feature distinct biophysical properties like, e.g., high molecular crowding that decreases diffusion of enzymes and thus may serve to promote interactions of enzymes acting in successive reactions.

Modeling approaches have suggested that bacterial cell poles accumulate large complexes such as ribosomes (~ 2 MDa) in the course of volume exclusion effects caused by the large central nucleoid [5], which has been observed for different bacteria [6, 7]. This polar distribution for larger assemblies has been proposed to be further enhanced by their increased hydrodynamic interaction potential (i.e., from larger molecule surfaces) and by entropic effects [5]. When large complexes come into proximity (caused by weakly attractive forces, e.g., Lennard-Jones Potential), they can exclude smaller molecules (the depletant; this can be water, ions, metabolites or proteins) from their occupied interfacial volumes, which results in an entropy gain of the system because many (excluded) smaller particles show less order than the unspecific complex interaction. In contrast to larger assemblies, smaller proteins were predicted to be directed towards less crowded regions, since their portion of excluded volume is smaller in those areas [8].

To address the question if metabolism of rod-shaped bacterial cells may be concentrated in specific subcellular regions, we set out to analyze four enzymes/enzyme complexes with strongly different sizes predicted to be cytoplasmic in the Gram positive model bacterium *Bacillus subtilis*. Like many other bacteria, plants, and fungi, *B. subtilis* is able to synthesize Riboflavin (Vitamin B2, RF) from minimal carbon and nitrogen sources. Four Rib enzymes, organized in an operon containing several internal promoters (Additional file 1: Figure S1A), namely 2,5-diamino-6-ribosylamino-4(3*H*)-pyrimidinone 5′-phosphate deaminase/5-amino-6-ribosylamino-2,4(1*H*,3*H*)-pyrimidinedione 5′-phosphate reductase (RibDG), GTP-Cyclohydrolase II/3,4-dihydroxy-2-butanone-4-phosphate-synthase (RibAB), dimethylribityl-lumazine-synthase (RibH), and RF-synthase (RibE), catalyze the six-step pathway from Guanosine triphosphate (GTP) and Ribulose-5-phosphate (R5P) to yield RF (Additional file 1: Figure S1B). RF is converted to its biological functional derivatives Flavin mononucleotide (FMN) by a bifunctional flavokinase/FAD adenylyltransferase, termed RibC, or further to Flavin adenosine dinucleotide (FAD) by the same enzyme. The encoding gene *ribC* is not part of the *rib*-operon structure comprising five genes in total, of which four are essential for RF-biosynthesis, whereas the fifth gene of the *rib*-operon encodes for a GCN-5-related N-acetyltransferase (Additional file 1: Figure S1A), for which so far, only its crystal structure is known [9, 10]. The first four steps of biosynthesis are carried out by the bifunctional enzymes RibAB and RibDG, which are assumed to be present as dimers and tetramers, respectively [11, 12]. RibAB catalyzes the first reaction, starting with GTP hydrolysis, guanine ring-opening, subsequent elimination of formate and release of 2,5-diamino-6-ribosylamino-4(3*H*)-pyrimidinone 5-phosphate (DAROPP, Additional file 1: Figure S1B). The two following reactions are carried out by the bifunctional enzyme RibDG, which first deaminates DAROPP to 5-diamino-6-ribosylamino-2,4(1*H*,3*H*)-pyrimidinedione 5-phosphate (AROPP) and further reduces the ribityl-side chain yielding 5-amino-6-ribitylamino-2,4 (1*H*,3*H*)-pyrimidinedione 5-phosphate (ARIPP). The latter compound is furthermore dephosphorylated by a so far unknown specific phosphatase, or a general phosphatase with broad substrate specificity. In a second reaction branch, RibAB catalyzes the conversion of the five-carbon precursor molecule R5P, derived from pentose phosphate pathway, to 3,4-dihydroxy-butanone-phosphate (DHBP, Additional file 1: Figure S1B). The dephosphorylated product, 5-amino-6-ribitylamino-2,4 (1*H*,3*H*)-pyrimidinedione (ARIP), as well as DHBP are both substrates for RibH, which catalyzes their condensation to yield 6,7-dimethyl-8-ribityllumazine (DMRL). Interestingly, RibH forms capsids, structurally best described as dodecamers built from pentamers. These 60-mer capsids harbor homotrimeric RibE in their lumen, generating substrate channeling for DMRL produced by RibH, which is then taken by RibE to generate RF and ARIP as products (Additional file 1: Figure S1B) [13, 14]. The detailed pathway and advances in biotechnological production of RF have been extensively reviewed [15–18].

Historically, this enzyme complex is termed “heavy RF synthase” or α_3_β_60_-complex and has been the object of intensive structural and biochemical research [19–22]. For a 3D structure modeled with PYRE2 in case of RibE [23] using published structures of RibH from *B.subtili*s and homologous RibE from *E.coli*, see Additional file 1: Figure S2 [24–26]. It has been considered as a drug target and may also have applications in biotechnology, vaccine carrier molecule development, and other fields due to its useful encapsulation abilities [27–29].

Given the splitting of the RF biosynthesis pathway into an apparently non-compartmentalized and a compartmentalized part, we investigated the spatial organization of Rib enzymes. We used *B. subtilis* because this organism is widely applied for industrial-scale production of RF [30, 31]. We generated functional fluorescent protein fusions to the four Rib enzymes encoded by the *rib*-operon, and provide evidence for transcription dependent, subcellular compartmentalization of capsid forming RibH and encapsulated RibE as well as for the existence of different diffusive subpopulations of the other Rib enzymes, and also analyzed their interplay with a particular focus on the two partner proteins of the heavy RF synthase.

## Results

### Enzymes of riboflavin synthesis show distinct diffusion coefficients and exhibit different spatial distributions

In order to study the dynamics of Rib enzymes, we generated C-terminal mVenus (mV) fluorescent protein (FP) fusions whose coding sequences were integrated into the respective original gene locus, with a xylose promoter driving continued transcription of downstream genes [32], which was required in case of the RibDG-mV fusion. In case of all other fusions, internal promoters within the *rib*-operon ensured continued transcription. This way, expression of fusion protein mRNA is driven by the original promoter and fusion enzymes are the only source of the protein within the cell. All fluorescent protein fusions described in this work supported wild type (wt)-like growth of cells on minimal medium, showing that they could functionally replace the wt proteins. We verified that only full-length fusion proteins were present by in-gel fluorescence detection (Additional file 1: Figure S3). Trajectories derived from FPs were acquired using single particle tracking (SPT) slimfield microscopy (Additional file 1: Figure S4, see corresponding Additional file 2: Movie S1 as an example) with subsequent post-processing of point-spread functions (PSF) and tracking by U-track [33], followed by statistical analysis of trajectories using SMTracker software [34, 35].

**Fig. 1:**
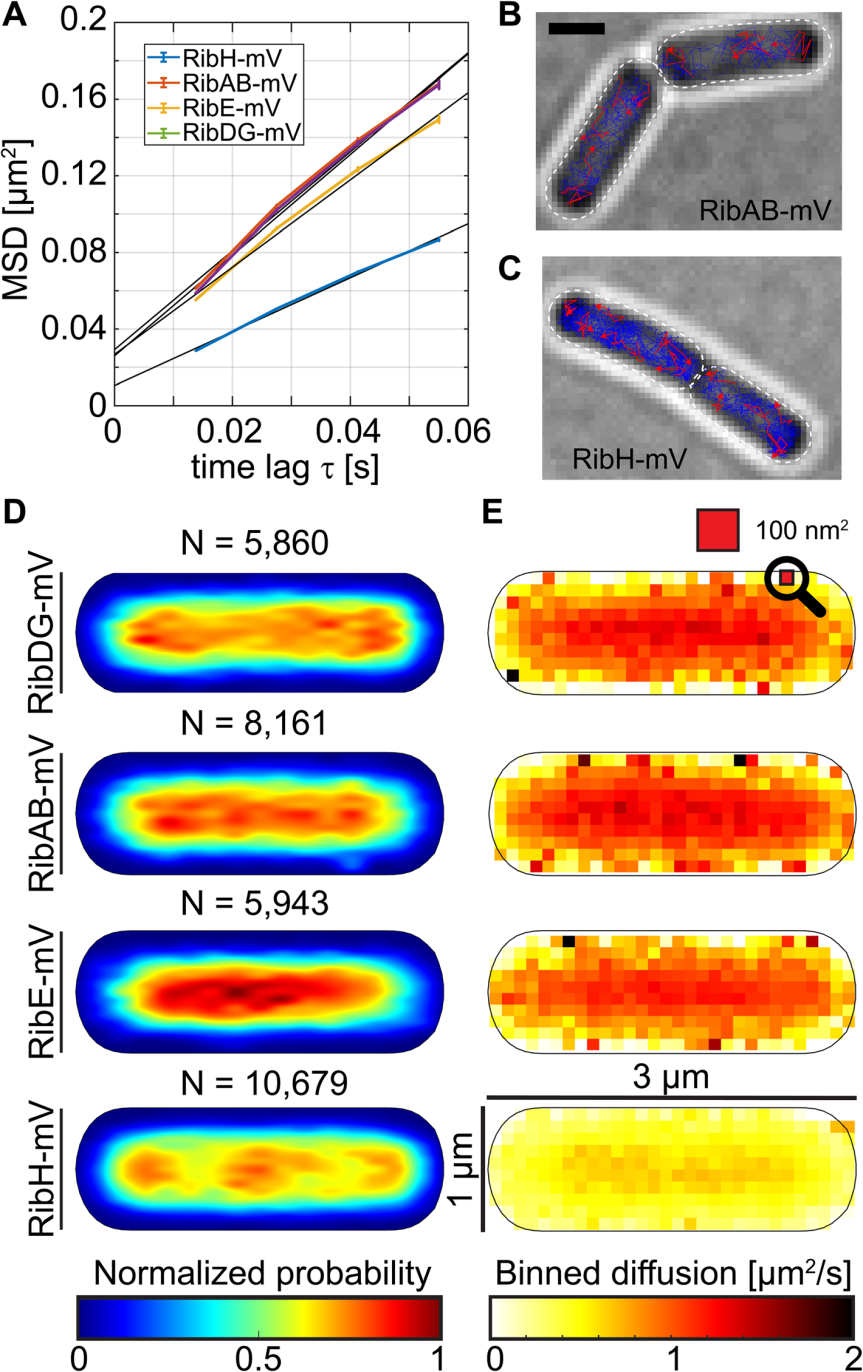
Characterization of Rib FP enzymes using SPT. **A** EAMSD versus time plot for the Rib FP enzymes RibDG-, RibE-, RibAB-, and RibH-mV. All SPT data taken for the EAMSD analysis and obtained results are summarized in Tables 1 and 2. **B**, **C** Bright field images of cells expressing *ribAB-mV* or *ribH-mV* from the original gene loci in exponential growth phases overlaid with trajectories derived from the according SPT datasets. Examples of trajectories (well separated examples were chosen) are shown in red. Scale bar represents 1 μm and is valid for both image montages. **D** Spot location heat maps displaying the spatial distributions of trajectories for all four Rib FP enzymes. Trajectories were projected onto the two dimensional cytoplasmic area of a normalized cell which resembles an averaged sized cell of *B. subtilis* in mid-exponential growth phase (1 μm × 3 μm). The likelihood of finding trajectories at a certain place in the cytoplasm is indicated by a color code from blue to red (indicated below the cell maps). Signal intensities of spatial distribution maps have been normalized with each other. **E** Speed maps displaying the spatial distributions of the average single step diffusions for all four Rib-mV fusion enzymes binned over areas of 0.1 μm^2^ for normalized cells

We first used ensemble-averaged mean squared displacement (EAMSD) analyses to describe the overall diffusion coefficients (DCs) for each fusion protein. RibDG-mV (determined to form a tetramer in vitro, which would correspond to 267 kDa including the FP fusion, Table 1), RibE-mV (trimer in vitro, 153 kDa with FP, Table 1), and RibAB-mV (dimer, 143 kDa with FP) showed similar DCs in the range of ~ 0.6 μm^2^/s (Fig. 1A, Table 1). In contrast, RibH-mV (2.7 MDa as 60-mer, Table 1) exhibited a much lower overall DC (~ 0.35 μm^2^/s), indicating obvious differences in size (Fig. 1A). Examples of randomly chosen trajectories are shown for RibAB- and RibH-mV fusions (Fig. 1B, C). Note that based on data measuring longitudinal diffusion along the long axis of cells, the DCs of some cytoplasmic proteins in *Escherichia coli* (*E. coli*) ranging from 26 kDa to 70 kDa have been shown to scale with the square of their radii, whereas proteins ranging from 70 kDa to 250 kDa revealed only a small size-dependence and reached a plateau at 0.8 μm^2^/s [38]. Thus, only large increments in protein mass appear to lead to considerable changes in diffusion. Measurements of cytoplasmic diffusion by, e.g., Fluorescence Recovery after Photobleaching (FRAP) do not allow distinguishing between different diffusive subpopulations; we applied SPT to determine the spatial distribution of fusion enzymes and to investigate if their dynamics may be based on different subpopulations having distinct DCs. Distinct subpopulations might be caused by protein-protein interactions, by high molecular mass (MM) cytoplasmic discontinuities such as the centrally compacted chromosome(s), called the nucleoid(s), by molecular crowding or by other unknown factors. To obtain estimates for cytoplasmic diffusion of non-fluorescently labeled Rib enzymes, as well as for their diffusion in less dense environments like under typical in vitro conditions with dilute buffered solutions, we applied an in silico approach using Hydropro software (http://leonardo.inf.um.es/macromol/programs/programs.htm), which calculates hydrodynamic properties (e.g., DCs) of proteins and their respective oligomeric assemblies based on available structural data, using either atomic-level structures or coarse-grained modeling for lower resolution structures [39] (Additional file 1: Table S1 [24, 25, 40, 41]). We assumed a value of 0.0529 Pa × s for the viscosity of the bacterial cytoplasm at room temperature (see Calculation S1, the “Methods” section), and a value of 0.001 Pa × s for the viscosity of dilute buffered solutions at room temperature, and took available structural data for Rib enzymes or closely homologous enzymes. From the comparison of obtained estimated (calculated) DCs to experimental data, one can measure up the diffusion of assumed oligomeric states of proteins, e.g., 60-mers and pentamers in case for RibH, as well will see later on.

**Table 1:** Charge analysis of Rib enzymes and their respective FP rib enzymes used for SPT in this study

Fusion enzyme/protein	N aa (pI)	N + charged aa (R, K)	N – charged aa (D, E)	Net charge monomer	Oligomeric state (MM in kDa)	Net charge of oligomeric state(s)
**RibDG**	361 (6.09)	+ 39	− 44	− 5	Tetramer (156.6)	− 20
**RibDG-mV**	611 (5.92)	+ 66	− 78	− 12	Tetramer (267.29)	− 48
**RibAB**	398 (5.64)	+ 43	− 58	− 15	Dimer (87.92)	− 30
**RibAB-mV**	648 (5.67)	+ 70	− 92	− 22	Dimer (143.28)	− 44
**RibE**	215 (5.87)	+ 22	− 26	− 4	Trimer (69.99)	− 12
**RibE-mV**	465 (5.78)	+ 49	− 60	− 11	Trimer (152.99)	− 33
**RibH**	154 (5.35)	+ 14	− 17	− 3	60-mer (968.4); 5-mer (80.7)	− 180* − 15
**RibH-mV**	404 (5.61)	+ 41	− 51	− 10	60-mer (2628.23); 5-mer (219.02)	− 600* − 50
**mV (fluorescent protein)**	239 (5.71)	+ 27	− 34	− 7	Monomer [36, 37] (26.89)	− 7

Returning to experimental data, we generated heat maps of spot locations to access the subcellular localization of enzymes (Fig. 1D). For this approach, trajectories were transformed into a heat map and projected into a normalized cell (1 μm × 3 μm) having an average size of *B. subtilis* during mid-exponential growth (Fig. 1D; also illustrated in Fig. 1E). The likelihood of finding a fluorescent protein at a certain place in the cytoplasm is indicated by a color code from blue (low occupancy) to red (high occupancy). Additionally, we quantified the likelihood of finding one of the fusion proteins along the long axis of cells using one-dimensional probability plots for different classes of cell sizes (Additional file 1: Figure S5A). We found that all four Rib enzymes are cytoplasmic, as expected, but intriguingly, with different preferences for subcellular locations (Fig. 1D), dependent on the size of the particular cell studied (Additional file 1: Figure S5A). For the bifunctional enzyme RibDG-mV, an increased probability for spotty localizations close to cell poles could be seen (Fig. 1D). This bipolar distribution is most apparent for larger cells. In case of medium and small cells, a spotty localization pattern was observed, however also arranged in a bipolar manner as evident by higher overall probabilities at the poles (Additional file 1: Figure S5A).

In comparison to RibDG-, RibAB-mV revealed a somewhat spottier localization pattern in the entire cytoplasm when averaged over all cell sizes (Fig. 1D). Differentiating those cells by size reveals a noticeable tendency of accumulation towards the cell poles and the periphery of the cytoplasm, especially for large cells (Additional file 1: Figure S5A).

While RibE-mV was visually evenly distributed throughout the cytoplasm and could be found with high likelihood at the central spaces of cells (keeping in mind that Bacilli cells are spherocylindrical), for RibH-mV, we observed the strongest bias for being positioned close to the cell poles or at mid-cell, most apparent in mid-sized to large cells (Additional file 1: Figure S5A). Cell poles are subcellular spaces where the nucleoid is absent, and the cell center just before cell division, when cells have two separated nucleoids, which will become the future new pole [6, 7] (Additional file 1: Figure S5C-D, “cell 4”). Thus, capsid-like RibH-mV shows clear nucleoid occlusion (NO), similar to what was observed for actively translating ribosomes [6].

Areas of highest likelihood for RibE-mV overlap only to a minor degree with those of RibH-mV (Fig. 1D), which is counterintuitive because both enzymes are known to form a bifunctional complex [26]. As we will see later, only ~ 14% of RibE-mV molecules are encapsulated in RibH nanocompartments, while a majority (85%) diffuses throughout the cytoplasm, in agreement with biochemical data showing that enzymatic activity of the heavy enzyme yielding RF can account for 22% up to 44% of the total activity depending on the strain studied [42]. High probabilities for enzyme localizations close to polar regions or mid-cell may be accompanied by a decrease in mobility in those regions; therefore, we binned single step diffusions over areas of 0.1 μm^2^ and created speed map representations for normalized cells able to reveal areas of different mobility for the same protein (Fig. 1E). Decreases in mobility might be caused by the large number of cytoplasmic proteins assumed to be present in high amounts at polar regions of the bacterial cytoplasm (molecular crowding and phase separation) and may be further enhanced by geometrical constraints of the cell poles. This analysis showed that RibDG- and RibAB-mV obey a similar distribution of single-step diffusions throughout the center of the cytoplasm where they tend to diffuse the fastest. Contrary to the center, the periphery of the cytoplasm as well as the cell poles represents spaces where these two enzymes generally diffuse slower (Fig. 1E). Similarly, the monofunctional RF synthase RibE-mV tends to diffuse the fastest in the center of the cytoplasm but shows a higher degree of slow diffusion at the cell poles compared to RibDG- and RibAB-mV (Fig. 1E). RibH-mV moved slowest compared to the other enzymes, however with a similar tendency of faster diffusion towards the long axis of the cytoplasm (Fig. 1E). The cytoplasmic periphery as well as the spaces close to cell poles and the at middle of the cytoplasm are spaces where RibH-mV diffused the slowest. Interestingly, those spaces coincide with the spaces of highest likelihood for the localization of RibH-mV (Fig. 1C–E), underlining our hypothesis of crowding for larger assemblies at areas where the nucleoid is absent.

### RibDG is mainly a freely diffusive enzyme

**Fig. 2:**
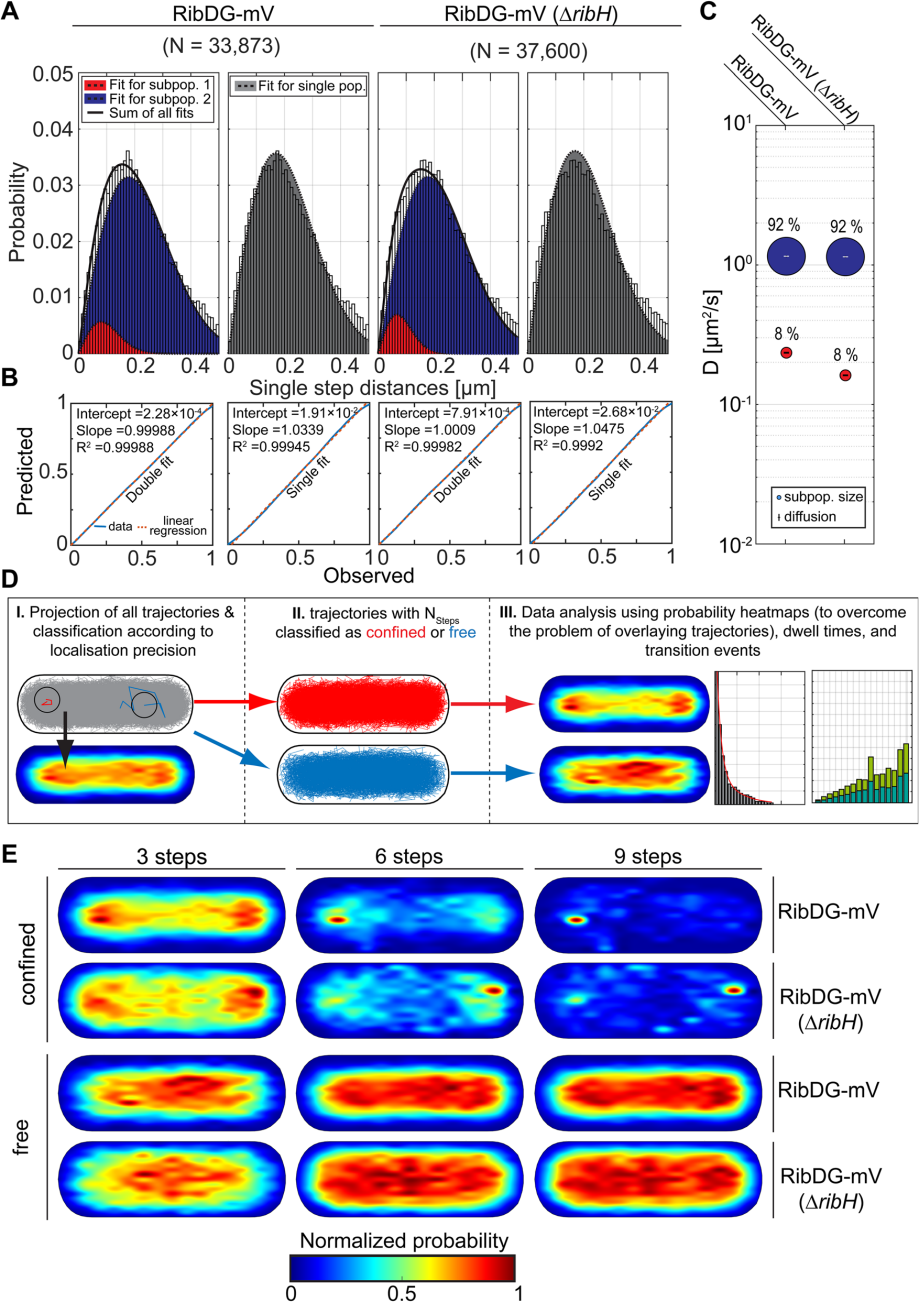
**A** Diffusion of RibDG-mV with respect to existing subpopulations and subcellular localization of free and confined trajectories. A Jump distance (JD) analysis of RibDG-mV and RibDG-mV (Δ*ribH*) SPT data using a two-component Rayleigh distribution model or a one-component Rayleigh distribution model (in gray). A non-linear least-squares fitting method has been applied to estimate their parameters, being the optimal number of components determined using statistical F-test for nested models. SPT data taken for this analysis and its results are summarized in Tables 1 and 2. **B** Model prediction-versus-observation plots for the Rayleigh distribution models of JDs in A. Data modeled based on Brownian motion are shown as a straight, red dotted line, the deviation of the observed data (fitted by double or single distributions, as indicated) to the modeled data is shown by the blue curved line, indicating very good agreement **C** Bubble plots displaying relative proportions of subpopulations for RibDG-mV and their according DCs that are derived from JD analysis in A. **D** Method for subcellular analysis of confined and free diffusing trajectories. (I) In the first step, all trajectories from a particular SPT dataset with at least five steps of consecutive localizations are projected onto a normalized 2D representation of the bacterial cytoplasm and transformed into a heat map displaying the overall spatial distribution of FP enzymes. (II) In the second step, the localization precision of each trajectory is estimated, an average value is calculated and further multiplied by a factor of 2.5 to yield the radius for a circle used for the analysis of confinement. (III) As a result of the analysis, two classes of trajectories arise (IV) which can be further transformed into separate heat maps. Trajectories can furthermore be analyzed in terms of transitions to confinement with respect to different numbers of confined steps yielding characteristic dwell times. **E** Subcellular analysis of confined and free trajectories shown for different numbers of consecutive steps projected onto the cytoplasm of a normalized cell representation. Displayed are the results using the radii given in Table S2 with three, six or nine consecutive steps of confinement

After having found that RibDG-mV shows a non-homogeneous localization, we analyzed if different diffusive populations may exist by applying Jump Distance (JD) analysis on the obtained SPT data [43]. JD pools the Euclidean distance between every two consecutive nodes of all trajectories and estimates the underlying probability density function (PDF) parameters with a Levenberg-Marquardt nonlinear curve fitting algorithm using Rayleigh distributions. We found that the RibDG-mV SPT dataset (Fig. 2A) cannot be fully modeled with a single distribution but is convincingly explained using a Rayleigh mixture model of two distributions (Fig. 2B), as judged from statistical F-test for nested models (Table 2). This result implies that the diffusive behavior of RibDG-mV can be explained best by the existence of two populations: a major, freely diffusive population (~ 92%) that has a mean DC of 1.16 μm^2^/s, and a minor subpopulation (~ 8%) of 0.23 μm^2^/s (Fig. 2C, Table 3). Although RibDG does not produce a substrate for the RibH/RibE complex, like RibAB does, we tracked RibDG-mV in a strain in which *ribH* had been replaced by an antibiotic resistance cassette [44]. Exponential growth of the deletion strain was ensured by the addition of RF to the minimal growth medium. We found that a deletion of *ribH* did not lead to a change in the number of fractions or to a change in population sizes itself, but slightly affected diffusion (0.16 μm^2^/s versus 0.23 μm^2^/s) of the slower moving population (Fig. 2A–C), which is likely caused by experimental or statistical variations.

**Table 2:** *p*-values derived from F-test for nested models (independent Rayleigh distribution modeling of JD distributions)

Strain relevant genotype	P_12_	P_23_	Bayesian information criterion	Final model
***ribDG-mV***	4.9303e−10	0.99993	Decrease less than 5% (pop. overestimation)	2 pop.
***ribDG-mV*(**Δ***ribH*)**	1.5694e−11	0.0043949	Decrease less than 5% (pop. overestimation)	2 pop.
***ribAB-mV***	1.1883e−6	1	–	2 pop.
***ribAB-mV*(**Δ***ribH*)**	0.34061	0.99947	Decrease less than 5% (pop. overestimation)	1 pop.
***ribE-mV***	3.0261e−7	0.00078246	Decrease less than 5% (pop. overestimation)	3 pop.
***ribE-mV*(**Δ***ribH*)**	1	1	Decrease less than 5% (pop. overestimation)	1 pop.
***ribH-mV***	0	0	–	3 pop.
***ribH-mV*(**Δ***ribE*,** ***incl. outliers)***	0	2.8755e−14	Decrease less than 5% (pop. overestimation)	3 pop.
***ribH-mV*(**Δ***ribE*,** ***excl. outliers)***	0	4.774e−15	–	3 pop.
***ribH-mV (control)***	0	0	–	3 pop.
***ribH-mV (30 min Rifampicin)***	0	0	–	3 pop.
***ribAB-mV (ectopic)***	5.4646e−5	0.99675	Decrease less than 5% (pop. overestimation)	
***ribAB-mV (ectopi*c,** Δ***ribH)***	1	1	Decrease less than 5% (pop. overestimation)	1 pop. (3 pop. with same DC)
***ribE-mV (ectopic)***	0.55303	1	Decrease less than 5% (pop. overestimation)	2 pop.
***ribE-mV (ectopi*c,** Δ***ribH)***	1	1	–	1 pop. (3 pop. with same DC)
***ribE-mV (ectopi*c,** Δ***ribE)***	0.090675	0.93867	Decrease less than 5% (pop. overestimation)	1 pop.

**Table 3:** Summary of diffusion coefficients (DCs) derived from EAMSD analysis using five lag times for all four Rib FP enzymes

Gene	Enzyme function(s)	MM of native monomer (MM of FP fusion [kDa])	Oligomeric state and MM of oligomer (MM of FP oligomer [kDa])	Diffusion [μm^**2**^/s] from EAMSD (R-value)	N of cells (N of movies)	N of trajectories per cell (N of total trajectories)	Average lifetime [s] (frames)
***ribDG (-mV)***	DAROPP deaminase/AROPP reductase	39.15 (66.82)	Tetramer 156.6 (267.29)	0.657 (0.994)	144 (49)	44 (5860)	0.092 (6.7)
***ribE (-mV)***	RF synthase	23.33 (50.99)	Trimer 69.99 (152.99)	0.570 (0.994)	94 (36)	70 (5943)	0.11 (7.7)
***ribAB (-mV)***	GTP cyclohydrolase II/DHBP synthase	43.96 (71.64)	Dimer 87.92 (143.28)	0.646 (0.992)	179 (55)	49 (8161)	0.1 (7.3)
***ribH (-mV)***	DMRL synthase	16.14 (43.80)	1. 60-mer 968.4 (2628.23) 2. Pentamer 80.7 (219.02)	0.352 (0.997)	76 (25)	143 (10,679)	0.14 (10)

To investigate if polar clustering of RibDG-mV might correlate with possible subcellular locations of confined particle movement, and to quantify observed transitions between freely diffusive and confined diffusive states, we applied an approach that employs a predefined radius of confinement (R): a trajectory is classified as confined if it can fit entirely (allowing one outlier event to account for stochasticity of molecule diffusion and for decrease in localization precision) inside a circle of R for a given number of steps (Fig. 2D). We chose R as 2.5 fold the mean estimated localization error (Additional file 1: Figure S6A, Table S2). The mean localization error was calculated from all estimated individual localization errors derived from the intersection points with the ordinate axis in time-averaged MSD (TAMSD) analyses (as described in the “Methods” section).

Using at least five consecutive steps of confined motion, we found that 28.1% of all trajectories of RibDG-mV were freely diffusive without any detectable switching from a free to a confined state or vice versa. On the other hand, we found that around 67.5% of trajectories underwent, at least partially, confined movement with characteristically small JDs before steps taken by these molecules changed towards free diffusion, judged from a larger JD distribution between consecutive steps (Additional file 1: Table S3). Therefore, we regard this fraction as subpopulation showing “mixed diffusive behavior.” In case of RibDG, transitions between confinement and free diffusion occurred with equal frequency (Additional file 1: Figure S7A), indicating an equilibrium process without any weight towards one state. When we analyzed RibDG-mV in a *ribH* deletion strain, we obtained very similar results in terms of transitions (Additional file 1: Figure S7A, Table S2), indicating robustness of analyses.

From SPT data, we extracted average dwell times for molecules, i.e., times molecules arrest in their movement, by plotting the Empirical cumulative distribution Function (1-ECDF), versus time of molecules showing the probability of dwelling for three or more consecutive steps within the radius of confinement (Additional file 1: Figure S7B, Table S2). We found that ECDFs of dwell times for few steps could be explained by a single exponential decay model (Additional file 1: Figure S7B), yielding an average of 50 ms (~ 4 frames), while for six or nine steps, two-component fitting dominated, indicating different populations for higher numbers of steps. In order to gain access to the subcellular localizations of the corresponding fractions, we projected the trajectories classified as confined or free into separated normalized cells. The graphical subcellular localization analysis revealed that confined trajectories of RibDG-mV obeyed a polar localization rule for different numbers of steps used and thus showed NO to a noticeable degree, similar to what we found for RibH-mV (Fig. 2E). These findings suggest that the spotty localization pattern described above (Fig. 1D) may be due to areas of low diffusion outside of the nucleoids, as also indicated by our speed map analysis (Fig. 1E). However, this effect was strongest for short events of confinement (three steps) and became less pronounced with increasing number of steps. Contrary to the polar, confined trajectories, those classified as free, were oriented towards the middle of the cytoplasm (Fig. 2E). Complementary to the decreasing polar localizations of confined trajectories for increasing numbers of steps, the more central localization of free trajectories was decreasing and thus freely diffusing molecules were distributed over the entire cytoplasm. As expected, very similar results were obtained for the auxotrophic strain carrying the *ribH* deletion (Fig. 2E).

In summary, although RibDG-mV should be a freely diffusive enzyme, it undergoes confined movement, mostly at regions that are devoid of the nucleoid. This behavior might be caused by the relatively large size of the tetrameric RibDG-mV (Table 1) but is not based on the presence of RibH.

### RibAB shows a minor degree of confined movement, influenced by RibH

**Fig. 3:**
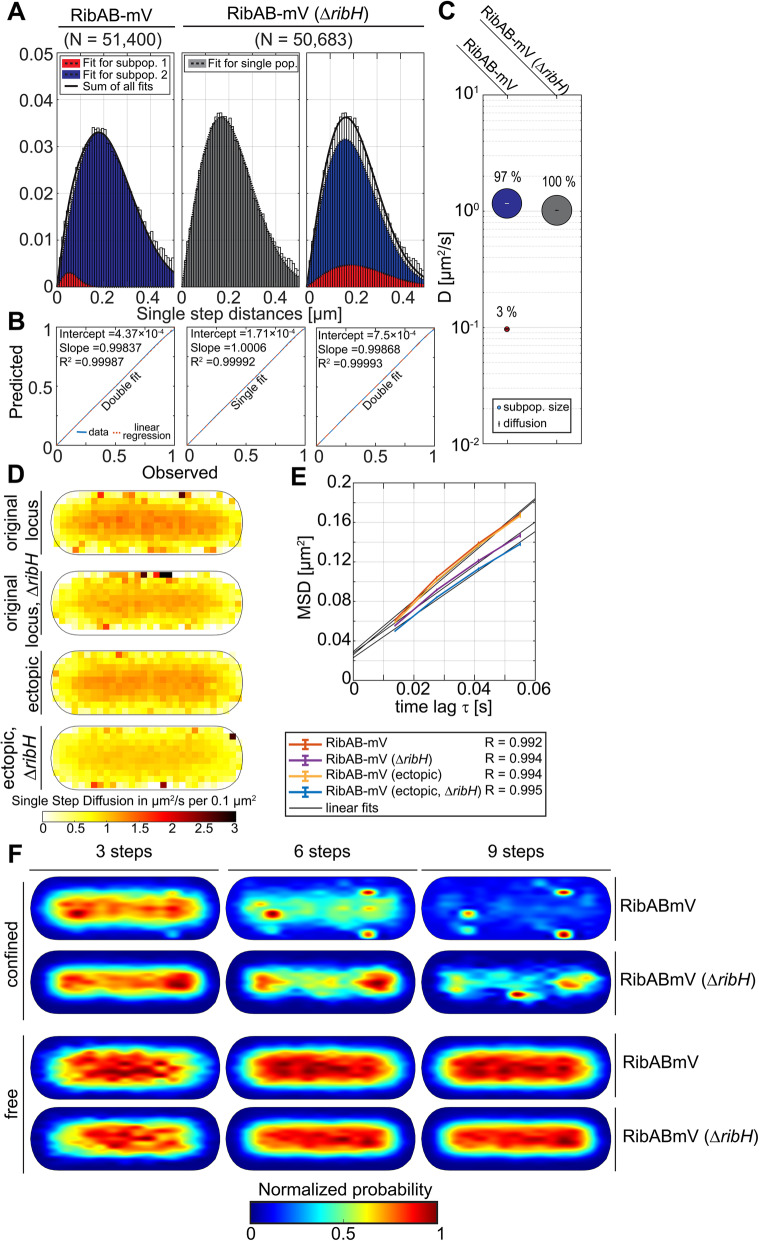
Comparison of diffusion and localization of RibAB-mV with and without RibH being present in the cytoplasm. For calculation of overall DCs from EAMSD, as well as, for JD analysis, we took a comparable number of trajectories (Tables 1 and 2). **A** Analysis of JD distribution for RibAB-mV in the presence and absence of RibH. JD distributions were fitted using two (left panel), one (middle panel), or two (right panel) Rayleigh distributions. The fitting algorithm used was the same as for RibDG-mV. **B** Model prediction versus observation plots for Rayleigh distribution modeling of JDs in A are displayed. **C** Bubble plots displaying relative proportions of diffusive populations and their according DCs (Table 3). **D** EAMSD analysis after five lag times for RibAB-mV in the presence (~ 0.63 μm^2^/s) and absence (~ 0.64 μm^2^/s) of RibH. **E** Speed map representations of RibAB-mV either in the presence or absence of RibH displaying the spatial distributions of single step diffusion binned over areas of 0.1 μm^2^ for normalized cells. **F** Subcellular analysis of confined and free trajectories for RibAB-mV either in the presence or absence of RibH. Displayed are the results using the radii given in Table S2 with three, six, or nine consecutive steps of confinement. The normalized probability is given below the normalized cell representations

RibAB plays a central role for RF biosynthesis by catalyzing the very first, as well as the fourth reaction. Thus, this bifunctional enzyme flanks the reactions catalyzed by RibDG, providing substrates necessary for RibDG as well as for RibH. It has been reported that native RibAB is a major bottleneck for RF biosynthesis due to its comparably low abundance at physiological level, and further because of its slow reaction kinetics in vitro*,* which are thought to be caused by slow release of DAROPP that is subsequently deaminated to AROPP by RibDG [45, 46]. JD distributions of RibAB-mV could be best represented by a two-population fit, having DCs of D_1_ = 0.0971 μm^2^/s and D_2_ = 1.17 μm^2^/s, with population sizes of ~ 3% and ~ 97%, respectively (Fig. 3A–C). Although a population size of 3% could be thought to be an experimental artifact, we note that a single fit was not able to explain the determined dynamics of RibAB. In contrast to the reaction described above, which is considered as the starting point of RF biosynthesis, the second reaction of RibAB, namely the conversion from R5P to DHBP, has not gained much attention. Because the synthesized DHBP is further used by RibH as a substrate (Additional file 1: Figure S1B), a putative transient interaction might allow for transfer of DHBP between both enzymes. Accordingly, we analyzed confined diffusive behavior of a strain expressing *ribAB-mV* from the original *rib*-locus in which *ribH* had been replaced by an antibiotic resistance cassette [44]. The deletion of *ribH* resulted in an unexpected increase in trajectories found per cell, so both datasets were adjusted at the level of trajectory numbers (Table 4). Interestingly, the JD distribution for RibAB-mV changed by the deletion of *ribH* (Fig. 3A–C)*,* which in this case was best described by a single diffusive population (D = 1.02 μm^2^/s), assuming two distributions did not make sense, because the DCs obtained were very similar (Fig. 3A, right panel). A similar observation was made when RibAB-mV was expressed at low level from an ectopic site on the chromosome in a *ribH* deletion strain (Fig. 3D, A–C, Table 4). Although these findings suggest that a small subset of diffusing RibAB-mV (~ 3%) is influenced by the presence of RibH, this number is too low to deduce any conclusion.

**Table 4:** Summary of DCs determined by EAMSD or JD analysis (independent fitting)

Strain relevant genotype	Diffusion [μm^**2**^/s] from EAMSD (R-value)	D_**1**_ [μm^**2**^/s] (Pop. %)	D_**2**_ [μm^**2**^/s] (Pop. %)	D_**3**_ [μm^**2**^/s] (Pop. %)	N of cells (N of movies)	N of trajectories per cell (N of total trajectories)	Av. life time [s] (frames)	Av. cell length [μm]
***ribDG-mV***	0.657 (0.994)	0.234 ± 0.002 (7.9%)	1.16 ± 0.001 (92.1%)	–	144 (49)	44 (5860)	0.092 (6.7)	2.86
***ribDG-mV*(**Δ***ribH*)**	0.682 (0.994)	0.162 ± 0.001 (7.69%)	1.14 ± 0.001 (92.3%)		126 (44)	52 (5918)	0.1 (7.4)	2.87
***ribAB-mV***	0.646 (0.992)	0.0966 ± 0.001 (2.6%)	1.17 ± 0 (97.4%)	–	179 (55)	49 (8161)	0.1 (7.3)	2.74
***ribAB-mV*(**Δ***ribH*)**	0.553 (0.994)	1.02 ± 0 (100%)	–	–	65 (19)	115 (7262)	0.11 (8)	2.82
***ribE-mV***	0.570 (0.994)	0.0287 ± 0.001 (1.3%)	0.514 ± 0.008 (14%)	1.12 ± 0.002 (84.7%)	94 (36)	70 (5943)	0.11 (7.7)	2.69
***ribE-mV*(**Δ***ribH*)**	0.691 (0.994)	1.3 ± 0 (100 %)	–	–	316 (121)	19 (5868)	0.091 (6.6)	2.75
***ribH-mV***	0.352 (0.997)	0.0815 ± 0 (10%)	0.375 ± 0 (65%)	1.01 ± 0.001 (25%)	76 (25)	143 (10,679)	0.14 (10)	2.80
***ribH-mV*(**Δ***ribE*,** ***incl. outliers)***	0.203 (0.989)	0.0164 ± 0 (16%)	0.168 ± 0 (37%)	0.684 ± 0.001 (47%)	80 (27)	133 (10,637)	0.17 (13)	2.81
***ribH-mV*(**Δ***ribE*,** ***excl. outliers)***	0.237 (0.993)	0.0319 ± 0 (9.1%)	0.18 ± 0 (40%)	0.691 ± 0.001 (50.9%)	79 (27)	134 (10,622)	0.16 (11)	2.82
***ribH-mV (control)***	0.329 (0.997)	0.0855 ± 0 (11.3%)	0.37 ± 0 (66.1%)	1.04 ± 0.001 (22.6%)	239 (83)	148 (33,386)	0.14 (10)	2.86
***ribH-mV (30 min Rifampicin)***	0.463 (0.997)	0.0665 ± 0 (3.79%)	0.475 ± 0 (64.2%)	1.11 ± 0.001 (32.01%)	227 (78)	161 (34,352)	0.13 (9.7)	2.73
***ribAB-mV (ectopic)***	0.653 (0.994)	1.13 ± 0.001 (94.4%)	0.343 ± 0.004 (5.65%)	–	201 (60)	51 (10,342)	0.12 (7.4)	2.70
***ribAB-mV (ectopi*c,** Δ***ribH)***	0.532 (0.995)	0.913 ± 0 (100%)	–	–	65 (27)	172 (11,186)	0.1 (8.5)	2.70
***ribE-mV (ectopic)***	0.589 (0.995)	1.1 ± 0.002 (98.8% ± 0.004)	0.539 ± 0.067 (98.8% ± 0.004)	-	95 (30)	126 (10,690)	0.11 (7.8)	2.90
***ribE-mV (ectopi*c,** Δ***ribH)***	0.556 (0.995)	1.08 ± 0 (100%)			63 (31)	178 (10,265)	0.11 (7.8)	2.88
***ribE-mV (ectopi*c,** Δ***ribE)***	0.514 (0.994)	0.917 ± 0 (100%)			111 (45)	102 (10,612)	0.11 (8.2)	2.69

To visualize our observations, trajectories were transformed to heat maps in normalized cell representations. Resulting heat maps for confinement at different numbers of steps (Fig. 3D) clearly show intense spots of confined diffusion for RibAB-mV under wt conditions close to cell poles and at the periphery of the cell. Contrary to the spotty confined trajectories, those classified as freely diffusing were oriented towards the long axis of the cell, and their normalized probability showed no bipolar preference but spotty patterns distributed over the central space occupied by the nucleoid(s). Using six steps, we found that the two component dwell times were similar between wt and *ribH* mutant cells (Fig. 3E, Additional file 1: Table S3).

Speed map representation of single step diffusion showed lower values for sites towards the cell periphery for RibDG (Fig. 3F), and subcellular analysis of confined and free trajectories revealed clear accumulation of confined motion at the cell poles, especially apparent considering six steps, while free diffusion occurred mostly in the center of the cylindrical cell (Fig. 3G).

### RibH shows a pattern of localization resembling NO that is disturbed in the absence of RibE

**Fig. 4:**
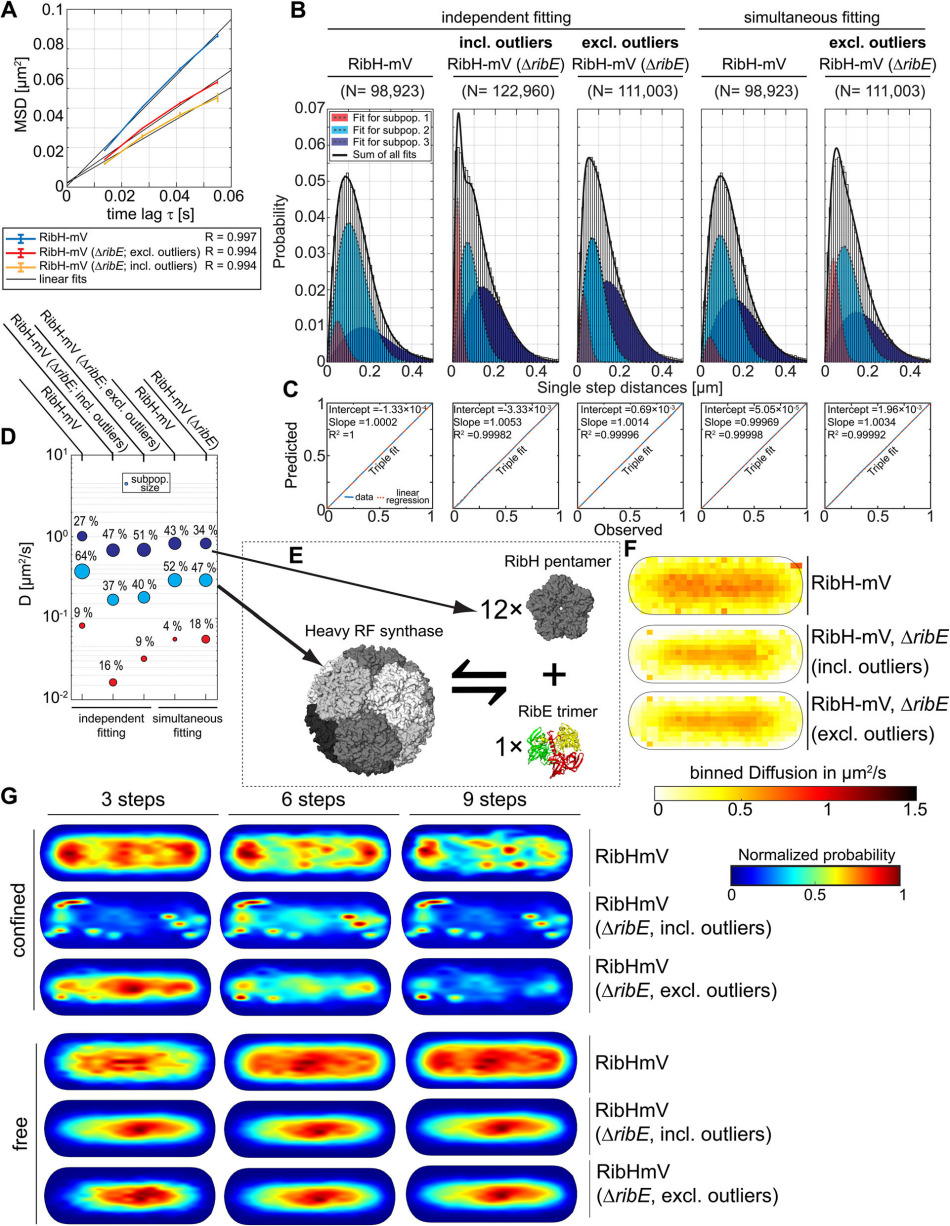
RibH-mV reveals three different subpopulations that change upon deletion of the gene encoding for interacting RibE. **A** EAMSD analysis of two strains expressing fluorescent RibH-mV in the presence or absence of RibE. In case of the *ribE* deletion strain results including outliers and results after their removal are shown. **B** Comparative JD analysis for the same strains as in A shows the existence of three diffusive subpopulations for RibH-mV. Those different subpopulations have been colored according to their mean DC derived from JD analysis (Table S2). Subpopulations in JD distributions were fitted using up to three Rayleigh distributions either fitted independently or simultaneously. **C** Model prediction versus observation plots for Rayleigh distribution modeling of JDs in B. **D** Bubble plot illustrating differences in relative proportions of subpopulations with respect to DCs derived from JD analysis. **E** Proposed model for encapsulation of RibE by RibH pentamers assuming either an equilibrium between free and encapsulated RibE (posttranslational mechanism), a cotranslational encapsulation mechanism, or a mixture of both mechanisms. **F** Speed map representations of RibH-mV either in the presence or absence of RibE displaying the spatial distributions of single step diffusion binned over areas of 0.1 μm^2^ for normalized cells. **G** Subcellular analysis of confined and free trajectories for RibH-mV either in the presence or absence of RibE and further with outliers removed for the deletion mutant. Displayed are the results using the radii given in Table S2 with three, six, or nine consecutive steps of confinement. The normalized probability is given on the right to the normalized cell representations

DMRL synthase RibH forms large spherical capsids with ~ 16 nm in diameter built from twelve RibH-pentamers and encapsulating homotrimeric RibE as a core enzyme (Additional file 1: Figure S2). RibH can be purified as a capsid from its native host *B. subtilis* or from *E. coli* as a recombinant enzyme and allows for C- or N-terminal fusion protein attachment without disturbing polymerization properties [29, 42, 47, 48]. Thus, we assumed that this FP enzyme would exclusively be present as a capsid in the bacterial cytoplasm, such that its comparably slow diffusion, as well as its NO (Fig. 1D, Additional file 1: Figure S5A), might be a consequence of its large spherical structure (Additional file 1: Figure S2). If this was the case, we would have expected to find only a single diffusive population. However, when we analyzed RibH-mV using JD distribution, data could best be explained by fitting with a model of three Rayleigh distributions. The populations had relative proportions of 9%, 64% and 27%, with DCs of D_1_ = 0.1 μm^2^/s, D_2_ = 0.38 μm^2^/s, and D_3_ = 1 μm^2^/s, respectively (Fig. 4B–D, Table 4). Comparing these results to the in silico analysis suggests that the fast population likely originates from a pentameric fraction predicted to diffuse with 1.1 μm^2^/s without mV. The intermediate population likely comprises heavy RF synthases (close to 1 MDa) that are estimated to diffuse with 0.57 μm^2^/s when lacking mV, as evident from in silico prediction using structural templates. However, in case of RibH-mV, forming a 2.63 MDa 60-mer, we calculated theoretical DCs of 0.32 μm^2^/s or 0.37 μm^2^/s, assuming either a squared or a cubic relation for the fully fluorescently decorated capsid, respectively (Calculation S1, the “Methods” section). The calculated theoretical DC assuming a cubic relation is in good agreement with our measured mean value of 0.38 μm^2^/s for the major subpopulation of RibH-mV, suggesting that diffusion of those capsids can be described best by the Stokes-Einstein equation, rather than by a squared relation.

As we will see in the next section, JD distribution analysis for RibE-mV revealed a subpopulation of 14% molecules diffusing with 0.51 μm^2^/s (Table 4). Because RibE-mV is encapsulated by native RibH molecules (about a 1 MDa complex, Table 1), the diffusion of heavy RF synthase formed entirely by RibH-mV is likely considerably lower, being a 2.63 MDa complex (Table 1), in agreement with the in silico prediction. Accordingly, we assume that a majority of 64% of RibH-mV single particles is present as capsids in the cytoplasm of *B. subtilis,* but a considerable number of molecules freely diffuses, most likely as pentamers, considered to be the minimal building blocks of the heavy RF synthases [26]. This subpopulation comprises 27% and has a mean DC (1.01 μm^2^/s), similar to that of the other major subpopulations of Rib FP enzymes (1.12 μm^2^/s – 1.18 μm^2^/s), which can be explained by their comparable sizes as oligomers (Tables 1 and 2). We could not find any subpopulations for RibH-mV that diffuse higher than 1.01 μm^2^/s (our setup allows us to detect freely diffusing mV molecules [49]), so we conclude that self-assembly kinetics of pentamers from individual RibH subunits is a rapid process that might not be temporarily resolvable by SPT. The slow-diffusing population has a DC similar to that determined for translating ribosomes [50]. We speculate that it could be an assembly intermediate, which is further discussed in the context of RibE.

Visual inspection of stream acquisitions (Additional file 2: Movie S1) shows numerous examples of apparent PSF fusion events for short time intervals, possibly representative for a dynamic equilibrium of capsid assembly and disassembly from pentamers. Interestingly, we observed a subpopulation comprising 9% of RibH-mV molecules that diffuses slowly with 0.08 μm^2^/s. In order to localize hotspots of low diffusion in a subcellular representation, we applied heat map analysis for free and confined trajectories. The latter were localized predominantly close to cell poles or at mid-cell (Fig. 4G), showing a pattern resembling NO, whereas the freely diffusive trajectories were distributed throughout the center of the cytoplasm, visually avoiding regions around the cell poles (Fig. 4G).

Curiously, the effect of confined diffusion outside of the spaces occupied by the nucleoid(s) transformed into a more punctuated pattern when RibE was absent compared to wt cells (Fig. 4G, Additional file 3: Movie S2), and numerous spotty localizations near cell poles were present in the deletion strain, whereas the wt strain showed a discrete bipolar pattern with some mid-cell preference (Fig. 4G). Furthermore, trajectories classified as freely diffusive in the deletion strain strongly centered around the middle of the cytoplasm whereas their intensity was lowest at the poles, indicating subcellular differences in diffusion between wt and the deletion strain.

This idea is further supported by speed map analysis, showing that subcellular diffusion at polar regions is considerably decreased when *ribE* was deleted. Complementary, we found that the deletion of the *ribE* gene led to a decrease in overall DC of RibH-mV compared to the wt strain when analyzed by EAMSD (Fig. 4A, Table 3), and also JD distributions were remarkably different, with all three respective mean DCs being reduced (Fig. 4B–D). Visual inspection of stream acquisitions revealed highly fluorescent spots that appeared almost immobile (Additional file 3: Movie S2) suggesting the formation of large proteinaceous aggregates containing high amounts of FP enzyme. When the average number of PSFs per frame and cell over the entire exposure time were quantified (Additional file 1: Figure S8), we found a decrease from two PSFs per cell to one in case for the wild-type strain and a slight decrease to 1.5 in case of the auxotrophic strain. However, this value remained constant for the entire exposure time, which suggests that a considerable number of RibH-mV molecules formed intracellular aggregates when RibE was absent.

Analyzing the deletion strain, we found three existing subpopulations, however, clearly shifted towards slower DCs, and with largely differing population sizes compared to the RibH-mV wt populations (Fig. 4B, D). Outliers that were three times longer than the average fluorophore lifetime of 1.2 s (these can be seen in the second panels Fig. 4B as increased slow-mobile fraction shown in red) were excluded from the analyses. By doing so, we aimed to ensure that the analysis of JD distribution is not biased towards low diffusion of obvious aggregates. Even without outliers, DCs were shifted towards slower diffusion for all three subpopulations present, and population sizes changed in case of the slowest population (Fig. 4B–D).

In order to obtain a better comparison of population sizes with the wt strain, we applied a simultaneous fitting to both SPT datasets. We found that the fast population, likely comprising pentamers, was decreased in the *ribE* mutant strain, whereas the slowest fraction was increased more than fourfold (Table 5). Contrary, the intermediate populations likely comprising assembled capsids were similar for both datasets, indicating that RibH can potentially form capsids without encapsulating RibE. Nevertheless, the changes in localization for free and confined trajectories, accompanied by the increase of the slow diffusing population, argue for a disturbance in the capsid assembly mechanism upon lack of RibE. These analyses underline the existence of on and off binding of pentamers to and from capsids, which might be connected to encapsulation of RibE by a mechanism of localized confinement at the cell poles.

**Table 5:** Summary of DCs determined by JD analysis (simultaneous fitting)

Strain relevant genotype	D_1_ [μm^2^/s] (Pop. %)	D_2_ [μm^2^/s] (Pop. %)	D_3_ [μm^2^/s] (Pop. %)	N of cells (N of movies)	N of trajectories per cell (N of total trajectories)	Av. life time [s] (frames)	Av. cell length [μm]
*ribH-mV*	0.0555 ± 0 (4.5%)	0.293 ± 0.001 (52%)	0.827 ± 0.003 (43.5%)	76 (25)	143 (10,679)	0.14 (10)	2.80
*ribH-mV* Δ*ribE (excl. outliers)*	0.0555 ± 0 (18.4%)	0.293 ± 0.001 (47.4 %)	0.827 ± 0.003 (34.2 %)	79 (27)	134 (10,622)	0.16 (11)	2.82

### Nucleoid occlusion (NO) of RibH depends on ongoing transcription

**Fig. 5:**
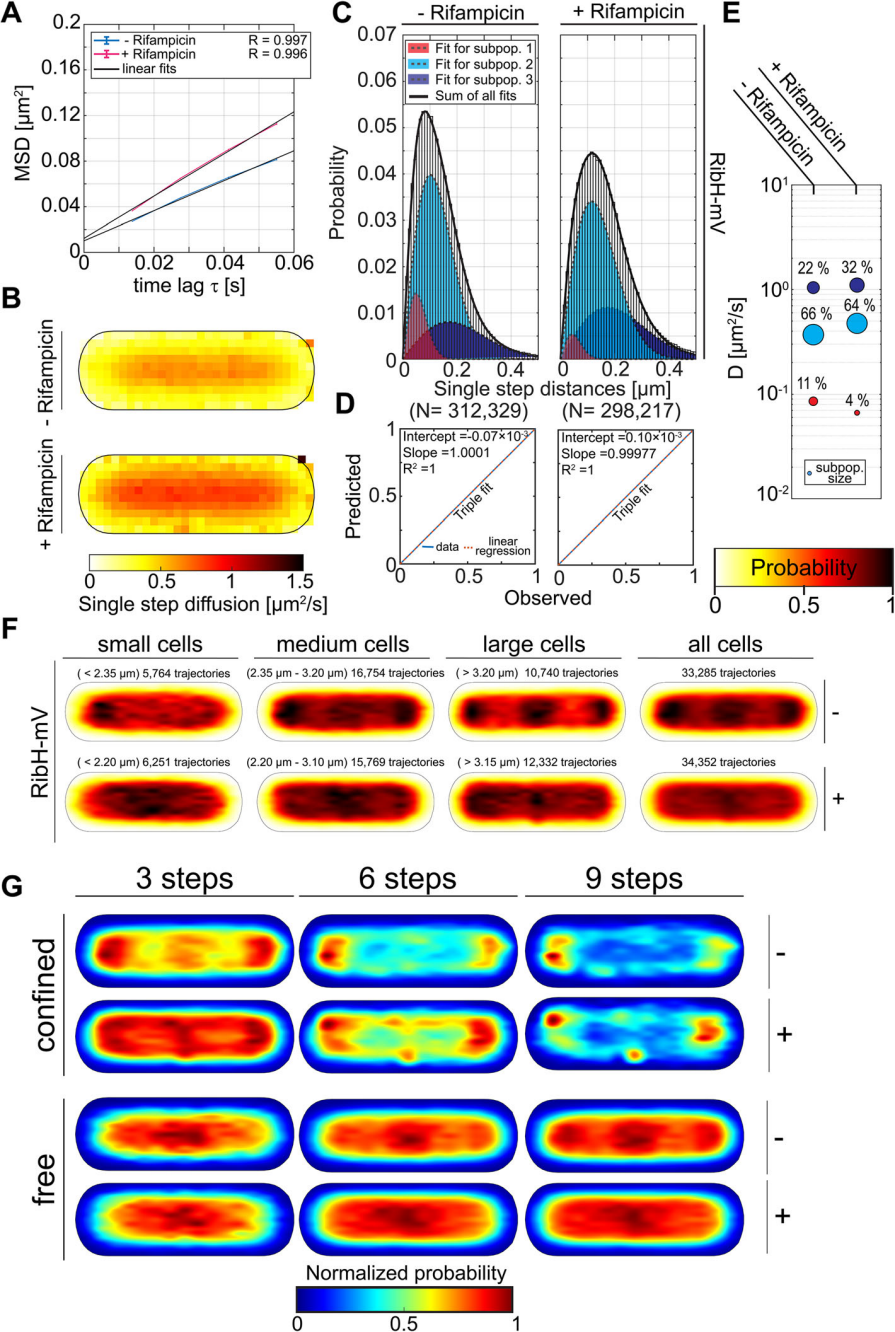
Nucleoid occlusion of RibH depends on ongoing transcription. All SPT data taken for the analysis and obtained results are summarized in Table 3. **A** EAMSD versus time plot for the Rib FP enzyme RibH-mV with and without added Rifampicin for inhibiting transcription by RNAP. **B** Speed map representations of RibH-mV with and without added Rifampicin displaying the spatial distributions of single step diffusion binned over areas of 0.1 μm^2^ for normalized cells. **C** Analysis of JD distribution for RibH-mV with and without added Rifampicin. JD distributions were fitted using up to three Rayleigh distributions. The independent fitting algorithm used was the same as used before. **D** Model prediction versus observation plots for Rayleigh distribution modeling of JDs in C are displayed. **E** Bubble plots displaying relative proportions of diffusive populations and their according DCs (Table 3). **F** Spot location heat maps with respect to different cell size classes for RibH-mV with and without added Rifampicin. For the analysis, cells were divided into three classes yielding small, medium, and large cells. Their respective trajectories were projected onto the two dimensional cytoplasmic area of those classified cells. The likelihood of finding trajectories at a certain place in the cytoplasm is indicated by a color code from white to black (indicated top right to the heat maps). Signal intensities of shown spatial distribution spot location heat maps for different cell sizes have been normalized with each other. Cell size intervals and the respective number of trajectories that have been considered are indicated on top of each heat map. **G** Subcellular analysis of confined and free trajectories for RibH-mV either with(+) or without(-) added Rifampicin. Displayed are the results using the radii given in Table S2 with three, six, or nine consecutive steps of confinement. The normalized probability is given on the right to the normalized cell representations

Confined trajectories of Rib enzymes are largely localized in a polar manner, suggesting that confined diffusion is located at regions devoid of the nucleoid(s). Those regions are usually spaces of ongoing translation, because fully assembled ribosomes also show NO [7]. Therefore, we used the RNA-polymerase (RNAP) inhibiting drug Rifampicin to study if NO of RibH depends on active transcription. After drug addition, the overall DC for RibH increased substantially (Fig. 5A). Rifampicin-treated cells also contained three existing subpopulations, but the fast and intermediate subpopulation were increased in their mean DCs, whereas the slow population was strongly decreased (about two fold) in size relative to subpopulations in non-treated cells. Contrary to untreated cells showing NO of RibH-mV, Rifampicin-treated cells revealed an almost equal distribution of intensities over the entire cytoplasm, which can be seen for cells of all sizes, clearly indicating a major loss of NO (Fig. 5F). Importantly, these experiments rule out that polar accumulation of molecules stems from proteins bumping into the cell membrane or from an interaction with membrane embedded/associated proteins and thereby arresting for some time, because this would equally happen in cells having a transcriptional downshift.

When confined and freely diffusive trajectories were analyzed in for a differential subcellular heat map analysis for different numbers of steps, the likelihood of finding confinement was increased all over the cytoplasm for shorter trajectories (Fig. 5G). These data show lower probabilities for polar localizations and higher probabilities for movement within spaces usually occupied by the central nucleoid(s) (Fig. 5G, Additional file 1: Figure S5). Thus, ongoing transcription in exponentially growing cells apparently influences diffusion and interaction of proteins, especially for large assemblies like the heavy RF synthase.

### RibE shows about 14% encapsulation within heavy RF synthase

**Fig. 6:**
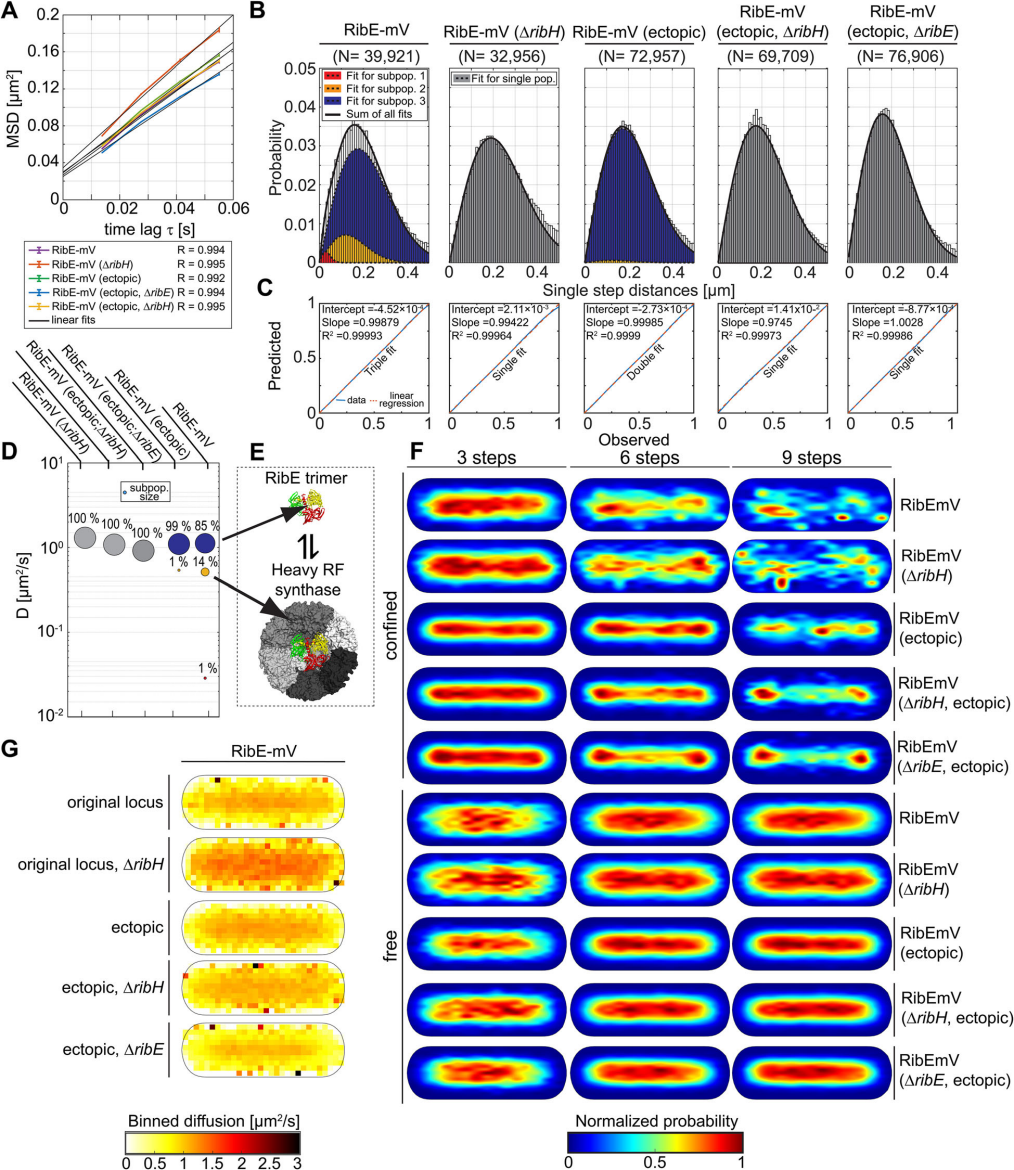
Diffusion analysis of RibE-mV and the impact of RibH on its confined motion. **A** EAMSD versus time plot for the Rib FP enzyme RibE-mV (original locus) in the presence and absence of RibH, as well as, for RibE-mV (ectopic locus) with and without native RibE or RibH being present. Datasets from the original loci and the ectopic loci were treated separately and equalized in terms of trajectory abundance. All results are summarized in Tables 1 and 2. **B** Comparative JD analysis for different strains producing RibE-mV (original locus) either in the presence (left) or absence (right) of RibH and for strains expressing *ribE-mV* ectopically with (middle) and without native RibE (right) or RibH (right to the middle) being present. To assess the existence of different diffusive subpopulations, we used a three-component Rayleigh distribution model and evaluated all possible models using statistical F-tests for nested models. The obtained results are summarized in Table 3. **C** Model prediction versus observation plots for Rayleigh distribution modeling of JDs shown on top in B. **D** Bubble plot illustrating the differences in relative proportions of subpopulations for both strains with respect to their mean DCs that are derived from JD analysis in B. **E** Proposed model for encapsulation of RibE by RibH pentamers assuming either an equilibrium between free and encapsulated RibE (posttranslational mechanism), a cotranslational encapsulation mechanism or a mixture of both mechanisms. **F** Subcellular analysis of confined and free trajectories for RibE-mV either in the presence or absence of RibH, as well as for RibE-mV (ectopic locus) with and without native RibE or RibH being present. Displayed are the results using the radii given in Table S2 with three, six, or nine consecutive steps of confinement. The normalized probability is given below the panel. **G** Speed map representations of RibE-mV (original locus) in the presence and absence of RibH, as well as for RibE-mV (ectopic locus) with and without native RibE or RibH being present. Displayed are the spatial distributions of single step diffusion binned over areas of 0.1 μm^2^ for normalized cell representations

Homotrimeric RibE is known to exist in two different forms: a free, monofunctional form, considered as the so called light RF synthase, and in its encapsulated form, in which it forms the core enzyme of the bifunctional nanocompartment heavy RF synthase, working in tandem with RibH and thus generating substrate-channeling [14]. We have shown that physiological localization and diffusion of RibH-mV is affected by the absence of RibE, so we next examined the dynamics of RibE at a single particle level. We found that that RibE-mV could be convincingly modeled by using a three-component Rayleigh distribution model, yielding three subpopulations with DCs of D_1_ = 0.03 μm^2^/s, D_2_ = 0.51 μm^2^/s, D_3_ = 1.12 μm^2^/s, having corresponding population sizes of 1%, 14%, and 85%, respectively (Fig. 6D). As stated before, the medium-fast population is likely encapsulated RibE, while the fastest population is freely diffusing RibE. Deletion of *ribH* led to a different JD distribution for RibE-mV, mainly showing a higher probability for having larger JDs accompanied by a decrease in probability for smaller JDs, indicating a higher overall diffusion as also evident by EAMSD analysis (Fig. 6A, Table 3). In the absence of *ribH,* the JD distribution could be best described using a single Rayleigh distribution, yielding one diffusive population with a mean DC of 1.3 μm^2^/s (Fig. 6B), strongly suggesting absence of encapsulation. This view is further supported by speed maps showing a clear bias towards faster diffusion in the cytoplasm including the polar regions for the *ribH* deletion strain, which was not the case when RibH was present (Fig. 6G). Additionally, we observed a threefold decrease in trajectories present per cell in the *ribH* deletion strain compared to the wt strain (Table 2). This decrease is likely caused by the required addition of RF to the growth medium, which activates the FMN riboswitch-driven transcriptional attenuation at the original gene locus [51, 52]. Therefore, datasets were equalized on the level of trajectory numbers, so that the analysis is statistically comparable (Table 4). Because the subpopulation of intermediate diffusion likely represents encapsulated RibE-mV, only 14% of these molecules are encapsulate in RibH 60-mers in vivo, and a majority of RibE-mV oligomers are freely diffusive, supporting the existence of the light RF synthase in vivo [42]. Thus 85% of RibE molecules are not available for substrate-channeling.

The coexistence of freely diffusive RibE and its encapsulated counterpart raises the question if transformation of a freely diffusive state into an encapsulated state is possible at all times by a posttranslational mechanism, or if encapsulation of homotrimeric RibE might only happen once, in a cotranslational manner. The observed small subpopulation of RibE-mV diffusing with 0.03 μm^2^/s (1%), which was absent in a *ribH* deletion strain diffuses similar to actively translating ribosomes in *E. coli* [53], which might indicate a cotranslational encapsulation mechanism (keeping in mind that such a small population may be an artifact). Interestingly, the immobile subpopulation of RibE-mV was absent when the gene was expressed from an ectopic locus (Fig. 6B–D). Additionally, encapsulation of RibE-mV decreased from 14% (*ribE-mV* expressed from the *rib*-locus) to only 1.2% (diffusing with 0.51 μm^2^/s, Fig. 6D, Table 4) when expressed from *amyE*, further supporting the hypothesis for a cotranslational encapsulation mechanism. As a further test, we investigated SPT data from a strain having *ribE* deleted from the original gene locus, and expressing the monocistronic mRNA for the fusion enzyme from ectopic *amyE* locus. EAMSDs analyses reveal that this strain has the highest decrease in overall diffusion (Fig. 6A), consisting of only one population diffusing with 0.917 μm^2^/s (Table 4), possibly indicating total absence of encapsulation. This idea is supported by comparison of speed maps of all strains expressing RibE-mV. Interestingly, the average DCs of ectopically expressed RibE were lowest at regions close to cell poles (Fig. 6G) when either *ribH* or *ribE* were deleted. Accordingly, we also observed an increased trend towards bipolar localization of confined trajectories for these two deletion strains compared to the non-ectopic strains, indicating that lower diffusion coincides with polar clustering of molecules. These findings notwithstanding, further experiments are required to either prove or refute the cotranslational encapsulation hypothesis. Interestingly, we noticed that the addition of RF to growth media was not required for exponential growth when *ribE-mV* was complemented from *amyE*, and deleted from original locus, however with no detectable encapsulation for RibE-mV as evident by JD analysis (Fig. 6B–E). Thus, we show that likely, RF biosynthesis (Additional file 1: Figure S1B) is not dependent on encapsulation of RibE by RibH and possibly works even without substrate-channeling.

### Anomalous and Brownian diffusion of Rib enzymes

**Fig. 7:**
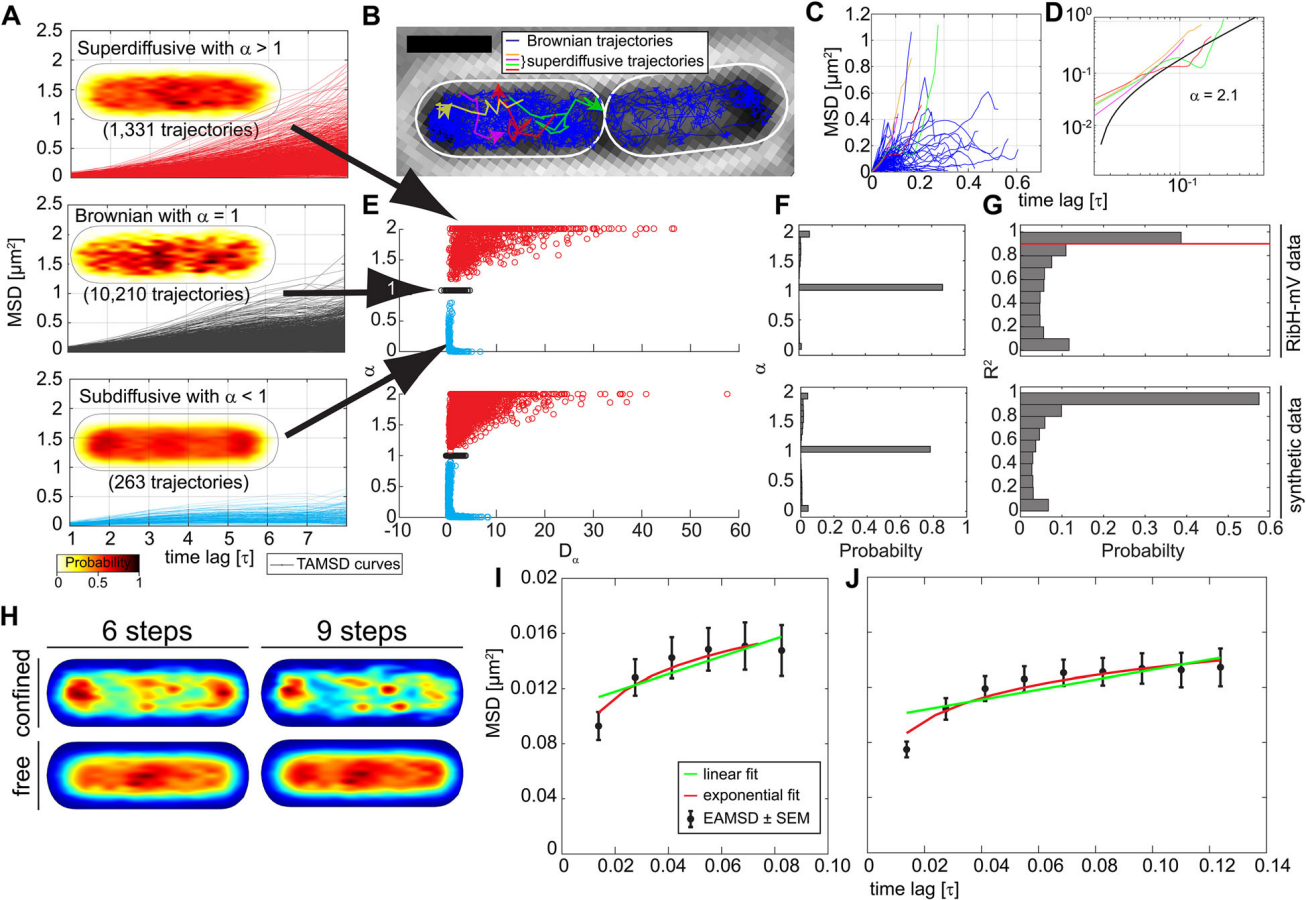
Mode of diffusion analysis for RibH-mV SPT data and comparison with synthetic data. **A** Individual TAMSD plots of trajectories derived from the RibH-mV SPT dataset sorted according to their mode of diffusion with inserted spot location heat maps displaying the localization of spots for those trajectories in a normalized cell representation. Signal intensities of spot location heat maps have been normalized with each other, and an absolute number of classified trajectories are given in brackets within the plot. **B** Image montage of a bright field microscopy picture overlaid with trajectories from exponentially grown PY79 expressing *ribH-mV*. Brownian trajectories are colored in blue; the four different identified superdiffusive trajectories in this particular cell are displayed in yellow, pink, red, and green. Scale bar represents 1 μm. **C** TAMSD plot for all trajectories displayed in the left cell of panel B. TAMSD curves of trajectories classified as Brownian are colored in blue, superdiffusive trajectories are colored as before in B. Shown are trajectories with R^2^ values that are at least 0.9 or higher (indicated in G as a red line). **D** Log-log plot showing TAMSD curves for superdiffusive trajectories shown in B. Colors are the same as before. **E** Probability plots showing the normalized probability of finding exponent α in our RibH-mV SPT data set (upper one with arrows) as well as for the synthetic data (lower one) generated with the parameters taken from JD analysis of the same dataset. Colors used are the same as in A. **F** Probability distribution of exponent alpha independently from its generalized DC for RibH-mV SPT data (upper one) as well as for the synthetic data. **G** Probability histograms displaying the likelihood of having an R^2^ value for individual fits of all trajectories from the RibH-mV dataset used before in F as well as for the synthetic data. The red line marks the threshold that has been used for selection of trajectories shown in C. **H** Subcellular analysis of confined and free trajectories for RibH-mV. Displayed are the results using the radius given in Table S2 with six or nine consecutive steps of confinement. The normalized probability is given below the normalized cell representations. **I**, **J** EAMSD plots with linear and exponential fitting for trajectories with at least six (I) or nine (J) consecutive steps classified as confined shown in H. The legend shown in I is also valid for J with indicated standard error of the mean (SEM)

The finding that confined movement of Rib enzymes mostly occurs at sites surrounding the centrally located nucleoids, as evident from heat map analysis of confined trajectories (Figs. 2E, 3G, 4G, 5G, 6F, 7H) raised the question if different subcellular locations offer different environments, resulting in different modes of diffusion. As a first indication for that, we have already shown that low diffusion of all RF fusion enzymes coincides with polar regions in the cytoplasm (Figs. 3F, 4F, 5B, 6G) and that the otherwise bipolar localization of RibH-mV can be disturbed by inhibiting RNAP, which revealed transcription dependency for confined diffusion at polar sites.

An explanation for different DCs within the cell would be if, e.g., molecules were crowded at high concentrations at the cell poles (where translation is mainly localized) and would move faster once they reached a region of less crowding which is/are the nucleoid(s) from the point of view of smaller proteins. So far, our report of diffusive properties for Rib enzyme fusions was based on analysis assuming Brownian motion, i.e., supposing a linear relation between observation time intervals and area being explored by an observable particle. Nevertheless, studies suggest that anomalous diffusion in cells exists [54–56] and modeling approaches have predicted non-Brownian motion emerging as a consequence of molecular crowding [57]. Anomalous diffusion follows a power law, <r^2^(τ) > ~τ^α^, exposing a non-linear relationship between the MSD and time. If the exponent α of this power law is equal to 1, it is called Brownian motion, but when α > 1 (superdiffusion) or 0 < α < 1 (subdiffusion), the diffusion is called anomalous (Formula S3). Our observations so far have revealed subpopulations with different DCs, and their respective transitions to confined states with characteristic dwell times (Additional file 1: Table S2), but this did not touch on the question of the mode of diffusion. We were therefore curious if the diffusional behavior observed for four very different enzymes might contain anomalous diffusion. To investigate whether anomalous diffusion can be observed for the four Rib enzymes, we determined the optimal fitting parameters for a particular TAMSD curve derived from a single trajectory, considering both a fixed α to 1 (assumption for Brownian motion) and free α (0 < α ≤ 2, assumption for anomalous motion). An ensuing hypothesis F-test for nested models decides which fitting yields best results to describe the mode of diffusion avoiding overfitting. To reliably fit TAMSD curves, we selected long trajectories to exclude artifacts generated by short numbers of steps and found that a length of at least nine consecutive localization steps was a good compromise between the number of trajectories and the bias towards Brownian motion for short-lifetime trajectories. We observed that approximately 90% of trajectories can be described best as having an exponent of one, meaning that Brownian motion clearly dominates over anomalous diffusion (Fig. 7F–H). However, we detected superdiffusion-like behavior for all enzymes for up to 10% (N > 1300 tracks) and additionally also apparent subdiffusion (Additional file 1: Figures S9 to S11). Due to limitations in trajectory length, we cannot exclude that molecules being detected as shorter trajectories (< 9 steps) might diffusive anomalously. With this caveat in mind, it appears that anomalous diffusion can be detected for a considerable number of molecules, a phenomenon that has been predicted by modeling studies as a consequence of molecular crowding at subcellular sites, and the existence of less crowded spaces [5].

Similar to Brownian motion across nucleoids (Figs. 5B, G, 6F, G), we observed that apparent superdiffusive trajectories are found with an increasing probability towards the center of cells occupied by the nucleoid(s) (Fig. 7A). As an example for such motion, we have depicted a cell overlaid with such anomalous trajectories derived from SPT data for RibH-mV (Fig. 7B). Cases of such apparent directed diffusion have been attributed to a diffusion process accompanied by advection, indicating a directionality of motion towards the long axes of rod-shaped cells [8]. Intriguingly, we found that superdiffusive trajectories with exponent α being > 1 or ≤ 2 exist for all other Rib enzymes to a very similar extent (Additional file 1: Figures S9 to S11). To probe if superdiffusion might occur at a particular subcellular space, we generated spot location heat maps for those trajectories that show either Brownian or anomalous (sub-, or super-) diffusion. Surprisingly, even though the nucleoids have been discussed to generate an “sieving” effect [38, 58], we found that apparent superdiffusion for RibH-mV has a higher probability towards the cell center, also reflecting the heat maps of freely diffusive trajectories, while subdiffusion represented the observed NO described above for confined trajectories (Fig. 7A, H). The trend that apparent superdiffusion occurs to a higher extent on the nucleoids can be seen for all three other Rib FP enzymes (Additional file 1: Figures S9, S10 and S11). The idea that nucleoids allow for faster diffusion of cytoplasmic proteins agrees with the finding that also average diffusion was higher at these positions than at the crowded cell poles as illustrated in the speed map analysis (Figs. 4F, 5B). However, in order to validate our results on the occurrence of anomalous diffusion in cells, we simulated two-dimensional diffusion of RibH-mV in a normalized cell according to Brownian motion with the subpopulation sizes and respective DCs derived from our JD analysis (Fig. 4, Tables 6 and 7). As before, we decided to include only trajectories of at least nine steps for reliable fitting. We found directed motion (α = 2) after nine steps in a ~ 3% of the tracks, and superdiffusion (α > 1) in around 13% of the tracks. Around 9% of trajectories were found to be subdiffusive (Table 7). The distinction was made using a significance F-test for nested models, with a confidence of 0.1.

**Table 6:** Input parameters for synthetic Brownian trajectories (*N* = 50,000) mimicking results derived from JD analysis of the RibH-mV SPT data

**Diffusion [μm**^**2**^**/s]**	0.08	0.375	1.01
**Population size**	10%	65%	25%

**Table 7:** Results for mode of diffusion analysis of synthetic Brownian trajectories

Mode of Diffusion	Brownian (α = 1)	Superdiffusive (α < 1)	Directed motion (α > 2)	Subdiffusive (α < 1)
**Proportion**	78.02%	10.16%	3%	8.82%

A comparison of those synthetic data with the original RibH-mV SPT dataset reveals very similar values for the relative proportions of Brownian and anomalous diffusion, suggesting domination of Brownian motion over anomalous diffusion, including a comparable proportion of sub- and superdiffusion (Fig. 7E–G). Contrary to seeming superdiffusion, which tends to appear in the cytoplasm along the long axis of the rod shaped cell, subdiffusion occurred mostly at the cell poles, in agreement with observed places of confined motion. When analyzing those confined trajectories by pooling them for an EAMSD analysis (Fig. 7I–J), enough evidence was found to not reject the existence of subdiffusion, indicating that subdiffusion at cell poles is a genuine effect intrinsically linked to confinement.

To further test if seeming superdiffusion are indeed fast-diffusing molecules, statistically arising from Brownian motion due to the stochastic nature of diffusion, we evaluated the classification of individual trajectories as sub- or super-diffusive based on a limited number of nine 9 steps with a hypothesis F-test for nested models comparing a linear (Brownian motion) and exponential weighted fit (anomalous motion). Weights were taken from the standard deviation of the MSD values at each interval time. These data show that except for RibDG-mV, where superdiffusive motion was rejected (*p* value of 0.213), in the rest of cases the existence of truly subdiffusive or superdiffusive motion was supported (Additional file 1: Figure S12).

Thus, our findings indicate that diffusion of Rib enzymes is mostly Brownian, with the exception of the cell poles where Rib enzymes tend to be confined, apparent as subdiffusion (Fig.7A, H, I, J). Our analyses suggest that there might be superdiffusion in cells for the four investigated enzymes, but more research is required to validate or dismiss this idea. The finding that freely diffusive trajectories and those classified as superdiffusive (either true or arising from the intrinsic variability of SPT data) do coincide with their central cytoplasmic representations (Fig. 7A, H) points out that nucleoids allow for fast diffusion of cytoplasmic proteins to exchange between crowded cell poles, which tend to confine protein motion potentially serving interactions between metabolic enzymes.

## Discussion

All life is based on the mobility of molecules within the cell or its interfaces. While Brownian motion and directed movement have been studied in great detail in eukaryotic cells, by default, mobility of cytoplasmic proteins within bacterial cells has been thought to be solely based on free diffusion for many decades. Interestingly, a recent report has shown that metabolic activity increases diffusion by fluidizing the cytoplasm [2]. In metabolic active cells, enzymes frequently undergo conformational changes in course of substrate binding, product release, or protein-protein interactions. These intra- and intermolecular dynamics are thought to be partially responsible for rearrangements of crowded regions allowing inert macromolecules like large viral protein capsids to escape from their immediate local surroundings [2]. Still, we are far from understanding the true mode(s) of protein mobility within non-compartmentalized cells and lack knowledge of possible spaces with different protein mobility within (bacterial) cells, which would generate metabolic channeling via enzyme crowding [59]. It has been shown that cell poles in several bacterial species are crowded by actively translating ribosomes, a process that is set up by RNAP actively synthetizing mRNA on the nucleoids, which is thought to be extruded towards the poles [6, 7, 50]. In this work, we provide evidence that bacterial cell poles and the periphery of the cytoplasm represent spaces of confined motion for enzymes, while centrally located nucleoids that contain the genome are places of fast Brownian motion. As this effect is also strongly reduced in the absence of active transcription, leading to more homogeneous subcellular diffusion of RibH, it appears that ribosome crowding also induces confined motion of other enzymes. We therefore propose that confined diffusion around nucleoids is a general property, especially for large enzymes or enzyme complexes, setting up subcellular spaces of enzyme and metabolite crowding that may speed up general metabolism in a fast-growing bacterial cell. In support of our findings, a recent study has shown that opposed to common views, nucleoids in *E. coli* cells do not present diffusion barriers [60].

We have characterized the localization, interplay and diffusion of the essential enzymatic pathway of RF biosynthesis using functional FP fusions. The pathway includes a capsid composed of 60 RibH subunits, known as heavy RF synthase, which achieves substrate channeling, together with its encapsulated RibE trimer [14]. Interestingly, RibE also exists in a non-encapsulated state, termed light RF synthase [42, 61]. Despite excellent biochemical research on this topic (reviewed in [15, 17, 62–65]), cell biological investigations of Rib enzymes have been missing. By taking SPT to a level in which different diffusive subpopulations of the same molecule species can be spatially depicted and quantified, we show that three enzymes of the RF biosynthesis pathway reveal confined diffusion occurring at the cell poles and the periphery of the cytoplasm, which for RibH was strongly reduced after inhibition of transcription. As indicated by the use of EAMSD plots for the tracks categorized as anomalous and our simulations, superdiffusion could exist in cells, though it is not clear to which extent, with a trend towards localization in the cell center where a majority of freely diffusive, fast trajectories localize. Furthermore, apparent superdiffusion revealed a trend towards the cell center where a majority of freely diffusive, fast trajectories was observed. Our findings indicate that for a protein traversing the nucleoid(s) is generally faster than motion at cell poles. This behavior of diffusing molecules can be explained in light of modeling approaches: authors found that a heterogeneous distribution of macromolecules in the bacterial cytoplasm model leads to a non-Gaussian behavior of displacements for smaller molecules like, e.g., single enzymes. As a consequence of introducing a heterogeneous distribution of crowding - directed motion, anomalous diffusion emerged stochastically, and this motion was calculated to tend towards regions of less molecular crowding [8].

We found localized confined motion at the cell poles for all enzymes, suggesting that proteins are crowded by their immediate surroundings represented by other intracellular molecules in those regions, as predicted by some studies [5, 8]. This uneven molecular distribution is likely the synergistic result of a complex interplay of physical phenomena in the bacterial cytoplasm, namely polydispersity of solute macromolecules (nucleoid(s), RNA, proteins, and their complexes), size-dependent volume exclusion effects (nucleoid occlusion e.g., of translating ribosomes), entropic effects, intermolecular attraction, and repulsion forces (electrostatics, hydrophobic effects) [5, 66]. Importantly, the sequestration of actively translating ribosomes seems to set off this uneven distribution, as we found that inhibition of transcription leads to a much more homogeneous localization of ribosomes [7] as well as of the Rib enzyme RibH (Fig. 5).

In case of capsid-forming RibH-mV, we show pronounced confined movement at the poles, which manifests itself as nucleoid occlusion (NO) for some, but not all RibH molecules. Molecular crowding at the cell poles appears to mainly affect larger assemblies like the heavy RF synthase and the RibDG tetramer, because the smaller enzymes RibE and RibAB showed NO only to a minor extent, if at all, whereas the center of the cytoplasm acts as a subcellular space that allows faster diffusion for all enzymes tested. Dwell time analyses suggest that confined motion surrounding the nucleoids occurs stochastically, indicating specific or unspecific interactions, which might be connected to spatial hindrance, in those areas. This notion is in agreement with modeling approaches of the bacterial cytoplasm in which unspecific interactions between differentially sized proteins, be they repulsive or attractive, have been shown to be an important factor to accurately reproduce in vivo derived DCs of bacterial FP fusion proteins [66].

Looking at RF biosynthesis in more detail, we provide evidence that confined motion of RibAB-mV and RibE-mV is to some degree dependent on the presence of RibH, which for RibAB may be indicative for transient protein-protein interactions, and encapsulation in case of RibE. In vitro, RibAB rapidly precipitates upon mixing with its first substrate GTP, for which major conformational changes exposing hydrophobic regions have been discussed [46]. However, it is conceivable that in vivo this increased hydrophobicity might be important for passive localization of enzymes to crowded regions, where interaction with subsequent enzymes RibDG or RibH occurs through crowding effects and possible direct interactions. Here, our data further support this idea by revealing spotty localization patterns of enzymes close to cell poles, mid-cell and the cytoplasm periphery for RibAB-mV, and for all other fusion enzymes as well. Interestingly, we observed different levels of encapsulation for RibE-mV, depending on the loci of corresponding gene expression, which may indicate co- and posttranslational encapsulation. We propose that a majority of capsids encapsulate RibE in a cotranslational manner, but that posttranslational dynamics also exist, because we observed encapsulation even when *ribE-mV* was expressed from an ectopic locus, however only if native RibE was also present. Nevertheless, in order to obtain a full picture of encapsulation dynamics, further studies are required.

Furthermore, our data underline the existence of a high proportion of light RF synthases, which have been found in biochemical studies [42, 61]. As these free RibE enzymes switch their diffusive states from free to confined, in a RibH-dependent manner, we propose that encapsulation dynamics may be the source of this behavior. In support of this idea, we found that RibH-mV likely exists in different oligomeric states, probably 60-meric capsids with encapsulated RibE, but also as freely diffusive pentamers. Therefore, our data strongly suggest an in vivo equilibrium between fully assembled RibH capsids containing trimers of RibE and free pentameric RibH oligomers. This idea gains further support by our finding that deletion of *ribE* leads to an apparent aggregation of RibH, supporting the importance of their interaction for proper capsid homeostasis in *B.subtilis*. This observation is also in agreement with in vitro experiments showing that upon chaotropically induced dissociation of the heavy RF synthase, stable light RF synthases are liberated from the capsid, which subsequently allow for renaturation of the heavy RF synthase by serving as their nucleation core [67]. In light of this, it is striking that heterologous RibH can be purified by itself as a capsid from *E. coli* [47, 68, 69] without the need for an encapsulated protein. However, proper 60-meric capsids can only be assembled in the presence of substrates (which are naturally present in *E. coli*) or substrate mimicry (in vitro), which have been shown to connect single subunits within pentamers, and so, seem to provide pentamer integrity and enhance capsid stability [67]. Empty capsids (containing substrates) obey the same overall 60-meric structural arrangement as in the heavy RF synthase; however, their sizes are slightly extended (⌀:17.4 nm) compared to that of heavy RF synthases (⌀:15.6 nm) [67], indicating capsid contraction induced by encapsulated RibE. Posttranslational dynamics of the heavy RF synthase, which are likely to be specific for *B. subtilis*, may also be involved in the process of encapsulation. The so far uncharacterized GCN5-like acetyltransferase RibT, whose gene forms the 3′ part of the *rib*-operon, could be a putative candidate performing posttranslational acetylation of Rib enzymes [9, 10]. Strikingly, purified RibH capsids have been shown to disassemble with a subsequent change of conformation into larger capsids (⌀: 29 nm) in a buffer-, pH-, and substrate-dependent manner [21, 67], and might thus be triggered to assemble as capsids from isolated pentamers by in vitro conditions. Besides that, it still remains mysterious how substrates or products enter or leave the active site or the lumen of the heavy RF synthase subunits, respectively. Each single RibH subunits comprises one half of a substrate binding pocket on each of its sites, so a single subunit is unable to bind any substrate. A pentameric RibH assembly comprises five full binding pockets and serves as the minimal building block for capsids [26], and these five active sites are not accessible from the exterior when assembled as a capsid. Further, the fivefold axis of each pentamer forms a channel that theoretically allows for passage of the substrates ARIP and DHBP, but it is too narrow for passage of the products RF and DMRL [19]. For this reason, a major conformational change combining catalysis and multiple assembly states of the heavy RF synthase based on structural investigations has been proposed [21]. Alternatively, capsid assembly and disassembly dynamics have been discussed to be guided by delicate electrostatic interactions between subunits of RibH pentamers [70]. Especially in the ligand-free form of capsids, the electrostatic attraction between RibH dimers (forming part of a pentamer) has been calculated to be decreased in comparison to the ligated capsids and thus a weakened dimer interaction has been proposed to precede capsid disassembly [70], which is also well in line with experimental observations in vitro [67]. Such a mechanism could provide a good explanation how substrates and products enter or leave the active sites of individual pentamers in vivo, respectively, and is supported by our findings that pentamers are in frequent exchange with 60-mers, apparently in a stochastic manner. A leaky behavior of the nanocompartment could also explain the occurrence of light RF synthases (non-encapsulated), which account for ~ 80% of total cellular RF synthase activity [67]. In light of our findings, these non-encapsulated enzymes could catalyze the formation of RF from the bulk DMRL that leaks from the heavy RF synthase because of the described assembly and disassembly dynamics.

## Conclusions

In toto, our results show that RF biosynthesis is dominated by free diffusion of enzymes, and that substrate channeling exists for a minor fraction in vivo. The finding that all four enzymes show clustering at the cell poles and the periphery of the nucleoids in the form of confined motion suggests that this is a general property of the bacterial cytoplasm for those species that contain condensed nucleoids and separated ribosomes, such as *E. coli* and *B. subtilis*, in contrast to *Caulobacter crescentus* with its apparently completely mixed cytoplasm [2]. We propose that enzyme clustering at the poles may speed up metabolism to a noticeable extent, and may also play a role in metabolic regulation in cells.

## Methods

### Bacterial strains and growth conditions

Bacterial strains used in course of this study are listed in Additional file 1: Table S4. For molecular cloning of plasmids and their propagation, we used the *E. coli* strain XL-1 Blue (Stratagene). All *E. coli* strains were routinely cultivated in liquid LB media at 37 °C under constant shaking (200 rpm) for plasmid preparations with addition of the respective antibiotics (Additional file 1: Table S5) ensuring selective pressure. For preparation of plasmids, we used Plasmid Mini Kit (Qiagen) according to the manufacturer’s instructions. Preparation and heat-shock transformation of competent *E. coli* XL-1 was achieved by standard protocols [71]. The *B. subtilis* strains created in course of this study were derived from the prototrophic laboratory strain PY79, the auxotrophic wild-type strain 168 or its respective deletion mutants. Strains of *B. subtilis* were routinely grown in liquid lysogeny broth (LB) media at 30 °C (200 rpm) for overnight culturing with addition of the respective antibiotics. For SPT microscopy overnight cultures were diluted 75 fold in glass tubes with 1 ml of freshly prepared S7_50_ minimal growth media containing glucose as a carbon source. These cultures were grown under constant shaking at 30 °C till reaching exponential growth phase (OD_600_: 0.4–0.6) until samples of live cells were taken for SPT microscopy experiments. S7_50_ minimal media was prepared according to published procedures [72]. In order to inhibit ongoing transcription by RNAP, cells of the strain PY79 *ribH-mV* were grown to exponential growth phase, as described before, and Rifampcin was diluted 1:1000 in the culture tube to yield a final concentration of 25 μg ml^-1^. Before performing further SPT studies, those cells were incubated for 30 min under the same growth conditions as noted before.

### Plasmid construction

We used the integrative single-crossover plasmid *pSG1164* [32] (Additional file 1: Table S6) to construct *pSG1164-linker-mVenus* in order to generate C-terminal mV fusion proteins replacing corresponding wild-type proteins. For this purpose, monomeric Venus (mV) coding sequence carrying the A206K mutation [36] was amplified by polymerase-chain reaction (PCR) using a lab stock plasmid carrying mV as a template, Oligonucleotides *Fw linker-mVenus ApaI* and *Rev mVenus SpeI* (Additional file 1: Table S7), Phusion DNA polymerase, and Deoxynucleotide solution (both from New England Biolabs, NEB). The resulting 771 bp PCR product was digested successively with *ApaI* and *SpeI-HF* yielding a 755 bp DNA fragment with cohesive 3′ overhanging ends; the vector *pSG1164* (4.8 Kbp) was digested accordingly and subsequently dephosphorylated by Calf intestine phosphatase (CIP, NEB); both linear DNA fragments were purified using agarose gel-electrophoresis followed by gel extraction (QIAquick Gel Extraction Kit, QIAGEN), ligated by T4 DNA ligase (NEB), and heat-shock transformed into chemical competent *E. coli* XL-1 Blue (Stratagene) to yield a strain carrying *pSG1164-linker-mVenus* as a plasmid. All further constructs with respect to integration at the original gene loci were derived from *pSG1164-linker-mVenus*. Similarly, we constructed *pSG1193-linker-mVenus* from *pSG1193* [73] in order to generate constructs able to ectopically integrate fusion enzyme genes into the *amyE* gene locus of *B. subtilis* PY79. To generate all final plasmids used to transform *B. subtilis*, we amplified the respective sequences by PCR using genomic DNA of *B. subtilis* PY79 as a template and adding the according oligonucleotides listed in Additional file 1: table S7. PCR products were purified, successively digested with restriction enzymes *AvrII* and *ApaI* (NEB) to generate in-frame fusions to the flexible 12 amino acids residue linker (G-G-S-G-G-G-S-G-G-G-S-G) coding sequence (5′-GGTGGAAGTGGAGGTGGATCAGGTGGAGGTTCTGGT-3′); *pSG1164-linker-mVenus* or *pSG1193-linker-mVenus* were digested accordingly and dephosphorylated by treatment with CIP, purified and subsequently ligated with the respective linear DNA insert fragment, and transformed into *E. coli* XL-1 Blue to yield the plasmids listed in Additional file 1: table S6. Correct insert sizes were confirmed by analytical restriction digestion of the respective plasmid using *XbaI* (NEB), and correct insert sequences were verified by Sanger sequencing (Eurofins).

### Strain construction

For construction of deletion strains, we transformed PY79 wt with chromosomal donor DNA of the respective 168 deletion strain (Additional file 1: Table S4) obtained from the BGSC [44]. Resulting transformants were carefully selected for RF deficiency, Kanamycin resistance, PY79 phenotype, and further used for transformation. To construct the strains used for our SPT measurements, we used the naturally competent *B. subtilis* strain PY79 as a recipient for the integrative *pSG1164-linker-mVenus* or *pSG1193-linker-mVenus* derived plasmids (Additional file 1: Table S6). Transformation of PY79 using was achieved by growing overnight cultures at 30 °C and 250 rpm in liquid LB media (10 grams Sodium chloride per liter). The next day, we used a 200 ml shaking flask to inoculate 10 ml of freshly prepared liquid modified competence media (MCM) with our overnight culture to yield an optical density of 0.08-0.1 measured at 600 nm (OD_600_). MCM was prepared according to published procedures [74]. The prepared culture was grown in MCM at 37 °C under constant shaking (200 rpm) to ensure proper aeration till it reached stationary growth phase indicated by an OD_600_ of 1.4–1.6. For subsequent transformation of plasmid or chromosomal DNA, we used an aliquot of 1 ml from that culture and added a total of 1 μg of the respective DNA to it (either plasmid or chromosomal DNA); as a control, we used 1 ml of the same culture without any addition of DNA. Each culture was further incubated at 37 °C in tubes with constant shaking for two more hours, followed by streaking out different amounts of culture aliquots onto fleshly prepared, solid LB-agar plates containing the appropriate antibiotics (Additional file 1: Table S5) to maintain selective pressure for the respective strain. In order to allow for efficient growth of generated deletion strains, we had to add minimal amounts of RF (1 μg/ml, Sigma-Aldrich) to all growth media used for their cultivation.

### SPT slimfield microscopy

To perform SPT experiments, we used Olympus IX-71 equipped with a high numerical aperture (NA) Total internal reflection (TIRF) objective (UAPON × 100, Oil, NA = 1.49), a back-illuminated Electron Multiplying Charge Coupled Device (EMCCD) iXon Ultra camera (Andor Solis) for rapid image acquisition, and a light emitting diode laser LuxX 515-100 (515 nm, 100 mW) for fluorophore excitation. Our laser beam was focused on the back focal plane and operated during image acquisition with up to 2 mW (60 W/cm^2^ at the image plane). Andor Solis software 4.21 was applied for imaging stream acquisitions of 1500 frames with 13.76 ms interval time (12 ms integration time). Acquired streams were equally cropped according to photobleaching curves to yield 1000 frames, adjusted for pixel sizes of 100 nm and time increments using Fiji [75]. Tracking of single particles was done by u-track 2.2 [33]. Trajectories were only considered for further statistical analysis if they had a length of at least five steps. Data analyses were done using SMTracker v1.5 [34], deposited at https://sourceforge.net/projects/singlemoleculetracker/.

### Calculation of dwell times

Dwell times are the average amount of time that a particle stays inside a circle of a certain radius centered in any point or node of the trajectory. For that matter, dwell times calculations need two parameters: the radius and the minimum number of steps that should remain inside the circle (1 step = 1 interval time). The procedure operates in such a way that maximizes how long the dwell event lasts (we call “dwell event” when a trajectory has at least one subset of nodes that fulfills the conditions). First, the node in the trajectory as the center of a circle which contains the maximum number of consecutive points of the trajectory is chosen, the amount of time the molecule stays is counted and the same track again previously excluding that segment of trajectory is explored. The procedure finishes when no more dwell events can be found. In our procedure, one gap (point absent for one frame) or one point outside the circle that goes and comes back are also considered to have remained inside the circle (for quantification purposes). The number of dwell events and their frequency is plotted in an Empirical cumulative distribution function histogram, and this data is fitted to a multi-exponential decay

$$ \sum \limits_{\mathrm{n}=1}^{\mathrm{n}=2}{\mathrm{A}}_{\mathrm{i}}\ast {\mathrm{e}}^{-{\frac{\mathrm{t}}{\uptau}}_{\mathrm{i}}},\mathrm{where}\sum {\mathrm{A}}_{\mathrm{i}}=1 $$

using a Levenberg-Marquardt nonlinear curve fitting algorithm, in order to distinguish up to two different populations of dwell times events In another plot, it is shown the average number of dwell events per length of the track and their respective standard error.

### Localization error estimation

In order to estimate average localization errors, we calculated the localization errors of all trajectories using the intersection point of the ordinate axis and the linear fit for five lag times in each individual TAMSD plot. This way, we calculated average values for the estimated localization error for each SPT dataset used in this study.

### Confinement maps

Determine where a protein has restricted movement is important to locate areas where it is interacting or to detect possible binding partners. To this end, a confinement map has been developed using the information given by the dwell times calculation algorithm. In the same way, a trajectory is considered to present confinement when it has at least one dwell event. This confinement can be total (confined track), partial (mixed behavior), or absent (freely diffusive). To build the confinement map, the free diffusive tracks are colored in blue. The rest are colored in green, while their confinement segment or partial trajectory that takes part in a dwell event is colored in red. Another plot is shown along with the confinement map, with the number and probability of a track of a certain length to do “transitions.” Transition means that a protein changes their state from confined to free and vice versa. It is considered that a track has transitioned from one state to the other if the trajectory, while not in confined state, traveled an average step distance longer than the confinement radius for at least three steps (3*interval time).

### Staining of live cell DNA and epifluorescence microscopy

For staining of DNA in live cells of *B. subtilis*, we have used the laboratory wt strain PY79. We have grown cells in S7_50_ minimal media (see Material and Methods section: “Bacterial strains and Growth conditions”) to early exponential growth phase, indicated by an OD_600_ of ~ 0.3 and subsequently added 4′,6′-diamino-2-phenylindole dihydrochloride (DAPI; Sigma-Aldrich) to a final concentration of 0.3 μg/ml. To allow for efficient DNA labeling, we incubated the cell suspension for 10 min with constant shaking (200 rpm). Cells samples were prepared as described in the Material and Methods section “Preparation of live cells for SPT experiments.” Samples were measured by microscopy using Zeiss Observer Z1 inverted epi fluorescence microscope equipped with an α Plan Fluor (NA: 1.54, oil immersion, × 100 magnification) objective, a fluorescence lamp (LQ-HXP 120-3 from Lighting & Electronics Jena), a EMCCD-camera Cascade II (512 × 512 pixels, with 16 × 16 μm physical pixel size, Photometrics), a 1.6× Optovar magnification, and a C-mount (2× magnification), resulting in a total magnification of × 3200 and a pixel size of 50 nm. Microscopy pictures were acquired using MetaMorph software (from Molecular devices) with 100–250 ms of integration time. For DNA imaging, a filter set allowing excitation of DAPI at 365 nm and passage of emission light at 445 nm was used. To draw cell outlines, bright field images of DAPI stained cells were captured and cell outlines were assigned using Oufti software [76]. DAPI-DNA fluorescence pictures have been background subtracted and treated with a Gaussian-Blur filter using Fiji [75]. For DAPI-DNA intensity measurements, we applied line plots along the entire longitudinal axis of each cell using Fiji [75] and plotted those values against their position.

### Preparation of live cells for SPT experiments

Cells were grown as described before and 3 μl of cell culture suspension were spotted on clean coverslips (25 mm, Menzel) and covered using 1% (w/v) ultra-pure agarose pads. Those pads were prepared before with fresh S7_50_ minimal medium by sandwiching melted agarose solution between two small 12 mm coverslips (Marienfeld). Coverslips for microscopy were intensively cleaned by ultra-sonication in Hellmanex II solution (2% v/v) for 30 min followed by intensive rinsing in distilled water and a second round of ultra-sonication in double distilled water.

### SDS-PAGE and fluorescence detection of fusion enzymes

For detection of fusion enzymes from whole cell lysates, we have grown the respective *B. subtilis* strains to exponential growth phase as described before (bacterial strains and growth conditions). Cell suspensions have been pelleted, washed in minimal media, and pelleted again discarding the supernatant for freezing. Next day, pellets have been suspended in buffer (100 mM NaCl, 50 mM EDTA, pH 7.5) and DNAse; RNAse and lysozyme were added to yield final concentrations of 1 mg/ml. To obtain cell lysates, the suspension was incubated for 30 min at 37 °C before SDS-sample buffer was added to yield a 10-fold concentrated lysate. In order not to quench mV derived fluorescence for imaging, samples were not cooked as usual. Samples of each strain were finally loaded on 12% SDS-PAGE gel for electrophoretic separation for 1 h at 4 °C using 50 mA and 120 V. The mV derived fluorescence from applied cell lysates was detected with Typhoon TRIO Imager System (GE Healthcare) using excitation at 488 nm.

### Simulation of synthetic data

Computer-simulated trajectories (*N* = 50,000) were generated inside a model cell with dimensions of 1 **×** 3 μm. To each track, T_i_, a diffusion coefficient D_j_ was assigned and keeping this diffusion along its lifetime and moving freely inside the model cell. Then, the location of the following step of each particle was generated following the equation

$$ {\mathrm{r}}_{\mathrm{i}+1}=\left({\mathrm{r}}_{\mathrm{i}}+{\updelta}_{\mathrm{i}}\right)+{\mathrm{E}}_{\mathrm{r}};{\updelta}_{\mathrm{i}}\sim \mathrm{N}\left(0,\sqrt{2{\mathrm{D}}_{\mathrm{j}}\uptau}\right) $$

Being ***r***_***i***_ the position of the particle in the frame *i*, δ_***i***_ Gaussian-distributed random variables with 0 mean and variance 2D_j_τ, and τ the interval time between simulation steps, and ***E***_***r***_ a gaussian error random variable, with standard deviation of 50 nm. Each observational step is divided by 100 sub-steps to assure the randomness of the procedure.

### Preparation of chromosomal DNA

In order to extract chromosomal DNA from *B. subtilis* 168 strains for transformation into PY79, we used phenol-chloroform extraction method [77]. After preparation, DNA was subjected to precipitation by 1-propanol for concentration, subsequently washed with 70% Ethanol (w/v), air dried, and resuspended in small amounts of TRIS-EDTA (TE) buffer.

### Amylase-assay

To probe successful integration of fusion enzyme coding sequences into the *amyE* locus of *B. subtilis*, we streaked single colonies from our transformation onto single wells of six-well plates that have been prepared with solid LB agar media containing 1% (w/v) of starch and respective antibiotics to maintain selective pressure, if needed. These plates were incubated overnight at 30 °C to allow for colony growth. As a positive amylase activity control, we streaked untransformed PY79 wt cells on one well of each plate. The next day, wells were slightly covered with Lugol’s iodine and incubated at 30 °C for 10 min to allow for visual screening of non-starch hydrolyzing colonies.

Calculation S1: RibH-mV forms 60-meric capsids with a corresponding MM of 43.403 kDa per single subunit resulting from C-terminal fusion to mV. Native, monomeric RibE accounts for 23.812 kDa. Using this values, we calculated a MM of 2,775.616 kDa for homotrimeric RibE entirely encapsulated by 60 subunits of RibH-mV. In contrast to the fully fluorescent capsid, a native heavy RF synthase would have a MM of 1,039.836 kDa with native RibH accounting for 16.14 kDa per subunit. In order to have an estimate for the mean diffusion coefficient of the heavy RF synthase, we calculated theoretical diffusion coefficients based on the Stokes-Einstein equation (Form. S1) and diffusion coefficients derived from JD analysis.

Stokes-Einstein equation: $\mathrm{D}=\frac{{\mathrm{k}}_{\mathrm{B}}\cdotp \mathrm{T}}{6\uppi \cdotp \upeta \cdotp {\mathrm{r}}_{\mathrm{H}}}$ (Formula S1)

(D: diffusion coefficient [m^2^/s], k_B_: Boltzmann constant [1.380649 × 10^-23^ J/K], T: absolute temperature [K], η: viscosity [Pa × s], r_H_: hydrodynamic radius of the diffusing particle [m])

Employing the Stokes-Einstein equation, we assumed that intracellular viscosity remains mostly constant for spherical particles having hydrodynamic radii of 8 nm (like the heavy RF synthase) or larger (like the heavy RF synthase formed by RibH-mV). As thermal energy is constant, as well, only the size of the particle studied is variable. However, suspecting the diffusion coefficients of the heavy RF synthase to be 0.51 μm^2^/s (as inferred from the diffusion of encapsulated RibE-mV) allows to calculate an estimate of the corresponding diffusion coefficient for heavy RF synthases formed by RibH-mV. This is simply done by calculating the ratios of cube roots for the corresponding MM of fully fluorescently RibH-mV capsids with encapsulated homotrimeric RibE and of the corresponding subpopulation for RibE-mV comprising 14% of relative population size (Fig. 5C). As RibE-mV is encapsulated by native RibH molecules, in this case, the MM is considerably different from the heavy RF synthases consisting of RibH-mV. Therefore, also diffusion is likely to be affected by these large differences in MM and thus in size. To rule out that diffusion of these protein complexes can be better described by a squared relation, as it has been found for a certain range of protein sizes, we also applied a calculation for a squared relation of ratios [38].

Assuming a squared relation between MM and diffusion:

$$ \frac{\sqrt[2]{\mathrm{MM}\ \left(60-\mathrm{meric}\ \mathrm{RibH}+\mathrm{trimeric}\ \mathrm{RibE}-\mathrm{mVenus}\right)}}{\sqrt[2]{\mathrm{MM}\ \left(60-\mathrm{meric}\ \mathrm{RibH}-\mathrm{mVenus}+\mathrm{trimeric}\ \mathrm{RibE}\right)}}=\frac{\sqrt[2]{60\ast 16286\ \mathrm{Da}+3\ast 50998\ \mathrm{Da}}}{\sqrt[2]{60\ast 43403\ \mathrm{Da}+3\ast }23812\ \mathrm{Da}}=0.6234 $$

Assuming a cubic relation between MM and diffusion:

$$ \frac{\sqrt[3]{\mathrm{MM}\ \left(60-\mathrm{meric}\ \mathrm{RibH}+\mathrm{trimeric}\ \mathrm{RibE}-\mathrm{mVenus}\right)}}{\sqrt[3]{\mathrm{MM}\ \left(60-\mathrm{meric}\ \mathrm{RibH}-\mathrm{mVenus}+\mathrm{trimeric}\ \mathrm{RibE}\right)}}=\frac{\sqrt[3]{60\ast 16286\ \mathrm{Da}+3\ast 50998\ \mathrm{Da}}}{\sqrt[3]{60\ast 43403\ \mathrm{Da}+3\ast }23812\ \mathrm{Da}}=0.7503 $$

The calculated ratios were further multiplied with the respective diffusion coefficient for encapsulated RibE-mV (Fig. 5, Table 4; 0.51 μm^2^/s). When calculated for a squared relation, we find a theoretical diffusion coefficient of 0.32 μm^2^/s for capsids formed by RibH-mV. In contrast to that, multiplying it with the result from using cube roots yields a diffusion coefficient of 0.37 μm^2^/s. This result is in very good agreement with our measurements showing a mean diffusion coefficient of 0.38 μm^2^/s for a major subpopulation of RibH-mV.

However, to get an estimate for intracellular viscosity faced by RibH and RibH-mV capsids and to calculate the theoretical radius of RibH-mV capsids, we additionally calculated these values by using the same diffusion coefficients as before (0.51 μm^2^/s for encapsulated RibE-mV) and the published radius for the heavy RF synthase (~ 7.8 nm) [78]. Further, we calculated the contribution of C-terminal fused linker-mV sequence to the hydrodynamic radius of capsids.

Stokes-Einstein with diffusion coefficient of encapsulated RibE-mV:

$$ 0.51\ {\upmu \mathrm{m}}^2/\mathrm{s}=\frac{{\mathrm{k}}_{\mathrm{B}}\ast 298.15\ \mathrm{K}}{6\uppi \ast \left(7.8\times {10}^{-9}\mathrm{m}\right)\ast \upeta} $$

Solving this formula for viscosity η:

$$ \upeta =\frac{{\mathrm{k}}_{\mathrm{B}}\ast 298.15\ \mathrm{K}}{6\uppi \ast \left(7.8\times {10}^{-9}\mathrm{m}\right)\ast \left(5.1\times {10}^{-13}{\mathrm{m}}^2/\mathrm{s}\right)} = 0.054(9)\ \mathrm{Pa}\ast \mathrm{s} $$

Insertion of calculated viscosity and solving the formula for the hydrodynamic radius r_H_ of 60-meric RibH-mV using the diffusion coefficient of 0.38 μm^2^/s (Fig. 4D, Table 4):

$$ {\mathrm{r}}_{\mathrm{H}\ 60-\mathrm{meric}\ \mathrm{ribH}-\mathrm{mV}}=\frac{{\mathrm{k}}_{\mathrm{B}}\ast 298.15\ \mathrm{K}}{6\uppi \ast 0.054(9)\ \mathrm{Pa}\ast \mathrm{s}\ast \left(3.8\times {10}^{-13}{\mathrm{m}}^2/\mathrm{s}\right)}=10.46(8)\ \mathrm{nm} $$

The calculated value for the contribution of linker-mV to the hydrodynamic radius of capsids formed by RibH-mV (10.46(8) nm–7.8 nm = 2.6(7) nm is well in line with previous studies that noted a hydrodynamic radius of 2.82 nm for the green fluorescent protein (GFP), which is very similar in sequence and structure to mV. Nevertheless, we expected a higher value since each radius of the heavy RF synthase should include one full diameter of an mV subunit, which in sum leads to an expected value of approximately ~ 13.6 nm.

Formula S2: Diffusion according to Brownian motion relies on a linear relation between time and area that has been explored by a particular particle. The linear relation allows to extract diffusion coefficients from the slope of an EAMSD curve or from the slope of a TAMSD curve derived from a particular trajectory:

$$ <{\mathrm{r}}^2\left(\uptau \right)>=2\mathrm{n}\cdotp \mathrm{D}\cdotp \uptau $$

(<r^2^(τ)>: mean squared displacement of an individual trajectory averaged over time (TAMSD) or of an ensemble-average of trajectories (EAMSD), n: dimensionality of the system, D: diffusion coefficient, τ: time lag)

Formula S3: Anomalous diffusion is characterized by a generalized diffusion coefficient (K_α_) which follows a power law with exponent α as the main characteristic. If α is equal to one, the diffusion behavior is called Brownian, and Anomalous otherwise.

$$ <{\mathrm{r}}^2\left(\uptau \right)>=2\mathrm{n}\cdotp {\mathrm{K}}_{\upalpha}\cdotp {\uptau}^{\upalpha} $$

(<r^2^(τ)>: mean squared displacement of an individual trajectory averaged over time (TAMSD), n: dimensionality of the system, K_α_: generalized diffusion coefficient, τ: time lag)

Calculation S2: We transformed the following formula [79] to calculate an estimate for the microscopic viscosity of the bacterial cytoplasm:

$$ \ln \left(\frac{{\mathrm{D}}_0}{{\mathrm{D}}_{\mathrm{JD}}}\right)=\ln \left(\frac{\upeta}{\upeta_0}\right)\kern0.50em \left(\mathrm{Form}.\mathrm{S}4\right) $$

(D_0_: diffusion coefficient in water at 25 °C [μm^2^/s]; D_JD_: diffusion coefficient from JD analysis [μm^2^/s]; η: viscosity of cytoplasm [Pa·s]; η_0_: viscosity of water at 25 °C [Pa·s])

As the diffusion coefficients of Rib enzymes have been calculated for the viscosity of water at 25 °C, we were interested in finding a suitable mean value for the viscosity of the bacterial cytoplasm at 25 °C. Since we measured the diffusion coefficients in vivo, we can use these values to calculate the cytoplasmic viscosity by transforming the upper formula into:

$$ {\mathrm{e}}^{\left(\ln \left(\frac{{\mathrm{D}}_0}{{\mathrm{D}}_{\mathrm{JD}}}\right)+\ln \left({\upeta}_0\right)\right)}=\upeta $$

For the calculation of a mean viscosity value, we first calculated all values for D_0_ at 25 °C for the viscosity of water (0.001 Pa·s) using Hydropro software [39, 80] and the structural files with their biological assembly (Additional file 1: Table S1) as an input. In the upper formula, we put in measured mean diffusion coefficients (D_JD_) from JD analysis for those subpopulations which we could assign to a distinct oligomeric state (Additional file 1: Table S1). Finally, we calculated a mean of 0.0529 Pa × s for the viscosity of the bacterial cytoplasm, which is in very good agreement with our value for the viscosity calculated by the Stokes-Einstein equation in Calculation S1 (0.0549 Pa × s).

## Supplementary Information

**Additional file 1: Figure S1.** Genetic organization of the *rib*-operon from *B. subtilis* and corresponding enzyme catalyzed reactions of RF biosynthesis. **Figure S2.** Model for heavy RF synthase. **Figure S3.** In-gel fluorescence detection of fusion enzymes from SDS-PAGE analysis displaying full length fusion enzymes. **Figure S4.** SPT method used to detect and analyze fusion enzymes in live cells of *B. subtilis.* **Figure S5.** Spatial distributions of detected spots for all four Rib FP enzymes in a cell size-dependent manner and localization of the nucleoid(s) in exponentially grown cells. **Figure S6.** Localization error histograms and cell size boxplots for all SPT datasets in this study. **Figure S7.** Transition analysis of RibDG-mV in the absence and presence of RibH and dwell-times for different numbers of confined steps. **Figure S8.** Spots detected per frame and per frame and cell for RibH-mV in the presence and absence of *ribE****.*** **Figure S9.** RibE shows different modes of diffusion in the bacterial cytoplasm. **Figure S10.** RibAB shows different modes of diffusion in the bacterial cytoplasm. **Figure S11.** RibDG shows different modes of diffusion in the bacterial cytoplasm. **Figure S12.** EAMSD plots for trajectories classified as anomalous of all four Rib FP enzymes. **Table S1.** Summary of confinement analysis from all SPT data used in this study. **Table S2.** Mean estimated localization errors and radii of confinement for each SPT dataset used in this study. **Table S3.** List of diffusion coefficients derived from structural data which have been calculated. **Table S4.** Strains used in this study. **Table S5.** Antibiotics and drugs used for cultivation of bacteria in this study. **Table S6.** Plasmids used in this study. **Table S7.** Oligonucleotides used in this study.

**Additional file 2: Movie S1.** SPT of tracked PSFs derived from RibH-mV. Fluorescence signal is derived from *B. subtilis* live cells producing the fluorescent fusion enzyme RibH-mV excited at 514 nm. Displayed are stream acquisitions of 1000 frames taken in 13.76 ms time intervals (12 ms integration time). Resulting trajectories from successful PSF localization and subsequent tracking are shown as red lines. Movie is shown in real time with 72.3 frames per second (fps). Cells have a size between 2 μm and 4 μm in length.

**Additional file 3: Movie S2.** SPT of tracked PSFs derived from RibH-mV using U-Track. Fluorescence signal is derived from *B. subtilis* live cells producing the fluorescent fusion enzyme RibH-mV excited at 514 nm in a Δ*ribE* deletion strain background. Displayed are stream acquisitions of 100 frames taken in 13.76 ms time intervals (12 ms integration time). Resulting trajectories from successful PSF localization and subsequent tracking are shown as red lines. Movie is shown in real time with 72.3 frames per second (fps). Cells have a size between 2 μm and 4 μm in length.

## Data Availability

All data described in this work are present within the manuscript and its additional files. The raw data (original movie files of terabyte dimensions) can be obtained from the corresponding author upon reasonable request.
