# Supplementary material for: Transcription-dependent confined diffusion of enzymes within subcellular spaces of the bacterial cytoplasm
Source: BMC Biol. 2021 Sep 2;19:183. doi: 10.1186/s12915-021-01083-4 (PMC8414670; doi:10.1186/s12915-021-01083-4)
Supplement: Supplementary file 1 — Additional file 1: Figure S1. Genetic organization of the rib-operon from B. subtilis and corresponding enzyme catalyzed reactions of RF biosynthesis. Figure S2. Model for heavy RF synthase. Figure S3. In-gel fluorescence detection of fusion enzymes from SDS-PAGE analysis displaying full length fusion enzymes. Figure S4. SPT method used to detect and analyze fusion enzymes in live cells of B. subtilis. Figure S5. Spatial distributions of detected spots for all four Rib FP enzymes in a cell size-dependent manner and localization of the nucleoid(s) in exponentially grown cells. Figure S6. Localization error histograms and cell size boxplots for all SPT datasets in this study. Figure S7. Transition analysis of RibDG-mV in the absence and presence of RibH and dwell-times for different numbers of confined steps. Figure S8. Spots detected per frame and per frame and cell for RibH-mV in the presence and absence of ribE. Figure S9. RibE shows different modes of diffusion in the bacterial cytoplasm. Figure S10. RibAB shows different modes of diffusion in the bacterial cytoplasm. Figure S11. RibDG shows different modes of diffusion in the bacterial cytoplasm. Figure S12. EAMSD plots for trajectories classified as anomalous of all four Rib FP enzymes. Table S1. Summary of confinement analysis from all SPT data used in this study. Table S2. Mean estimated localization errors and radii of confinement for each SPT dataset used in this study. Table S3. List of diffusion coefficients derived from structural data which have been calculated. Table S4. Strains used in this study. Table S5. Antibiotics and drugs used for cultivation of bacteria in this study. Table S6. Plasmids used in this study. Table S7. Oligonucleotides used in this study. [file 12915_2021_1083_MOESM1_ESM.docx]

**Supplementary data**

**Supplementary figures**

**
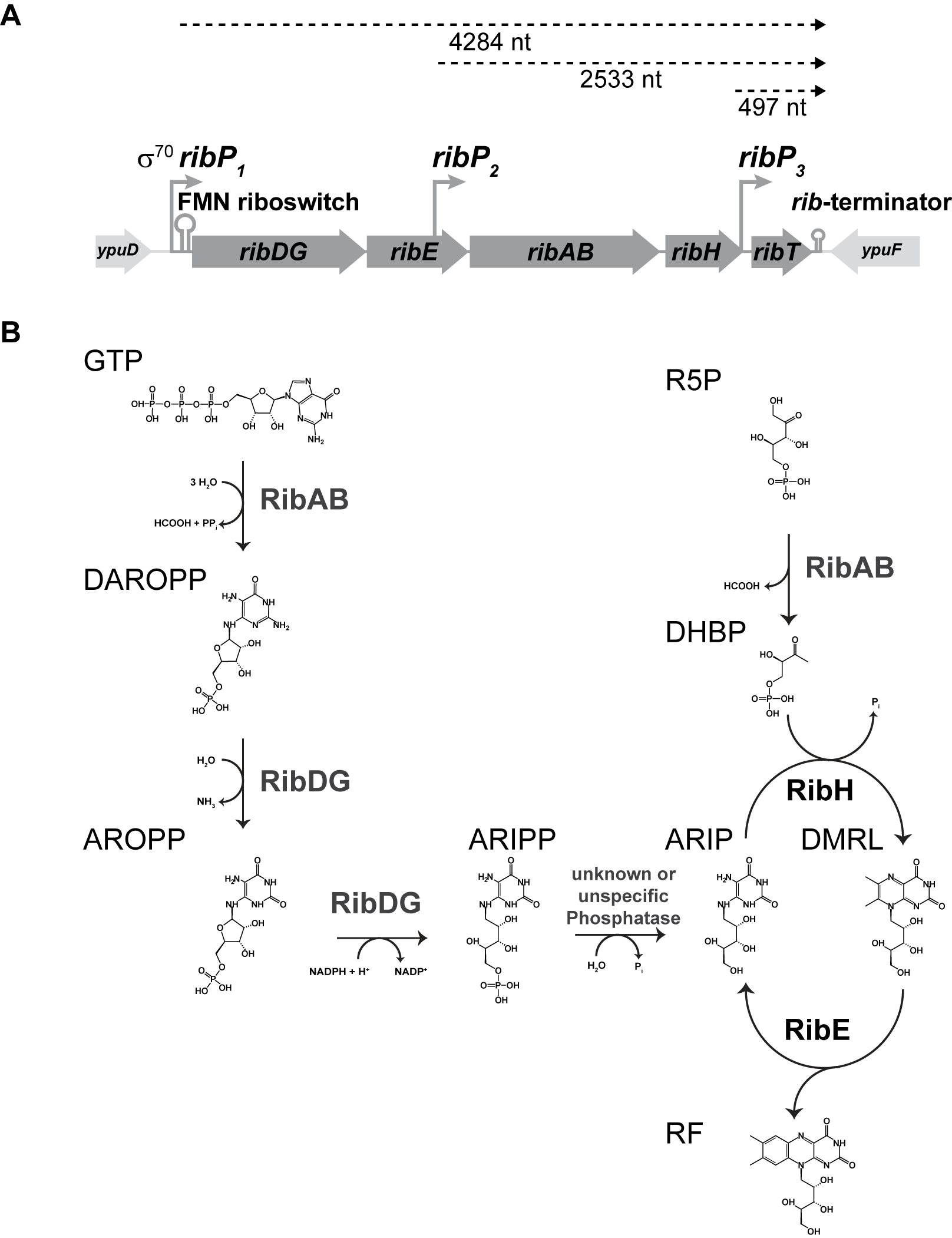
**

**Figure S1: Genetic organization of the *rib*-operon from *B. subtilis* and corresponding enzyme catalyzed reactions of RF biosynthesis. (A)** The *rib*‑operon of *B. subtilis* comprises five genes in total of which four (*ribDG*, *ribE*, *ribAB*, and *ribH*) are essential for RF biosynthesis. A schematic representation of the *rib*-genes (dark grey) and the genetic organization of the operon is displayed. Transcription start sites for the major σ^70^-dependent promotor, the two other minor promotors and the different resulting polycistronic mRNAs are indicated, as well as, the position of the regulatory region (FMN riboswitch) and the final terminator structure. Gene products of the *rib*‑genes and their respective abbreviations used throughout this study are displayed. **(B)** Biosynthesis of RF in *B. subtilis* at a glance with a brief description of the pathway: RibAB (GTP cyclohydrolase II (A) (EC 3.5.4.25) catalyzes ring opening of Guanosine triphosphate’s (GTP) imidazole group as well as cleavage of the phosphoanhydride bond resulting in pyrophosphate release. The resulting product 2,5-Diamino-6-(ribosylamino)-4(3*H*)-pyrimidinone 5'-phosphate (DAROPP) is converted in two enzymatic reaction steps by the bifunctional enzyme RibDG (DAROPP deaminase (D) (EC 3.5.4.26)) starting with the deamination at the position two amino group at the pyrimidine ring. The side chain of the second product 5-amino-6-(ribosylamino)-2,4-(1*H*,3*H*)-pyrimidinedione 5'-phosphate (AROPP) is subsequently reduced by RibDG (AROPP reductase (G) (EC 1.1.1.193)) resulting in the formation 5-Amino-6-ribitylamino-2,4 (1*H*,3*H*)-pyrimidinedione 5'-phosphate (ARIPP), which is further dephosphorylated to by a so far unknown phosphatase. RibH (DMRL synthase (EC 2.5.1.78)) catalyzes the regiospecific condensation of the dephosphorylated product 5-Amino-6-ribitylamino-2,4 (1*H*,3*H*)-pyrimidinedione (ARIP) with 3,4-Dihydroxy-2-butanone-4-phosphate (DHBP) to yield 6,7-Dimethyl-8-ribityllumazine (DMRL). DHBP is obtained from Ribulose‑5-phosphate (R5P) in stereochemical controlled rearrangement and elimination reactions catalyzed by RibAB (DHBP synthase (B) (EC 4.1.99.12)). Finally, RF biosynthesis involves a highly unusual dismutational reaction catalyzed by RibE (RF synthase (EC 2.5.1.9)) that transfers a four-carbon unit between two DMRL molecules resulting in formation of RF and ARIP, whereas the latter compound servers as a substrate for RibH and is further recycled by the heavy RF synthase.


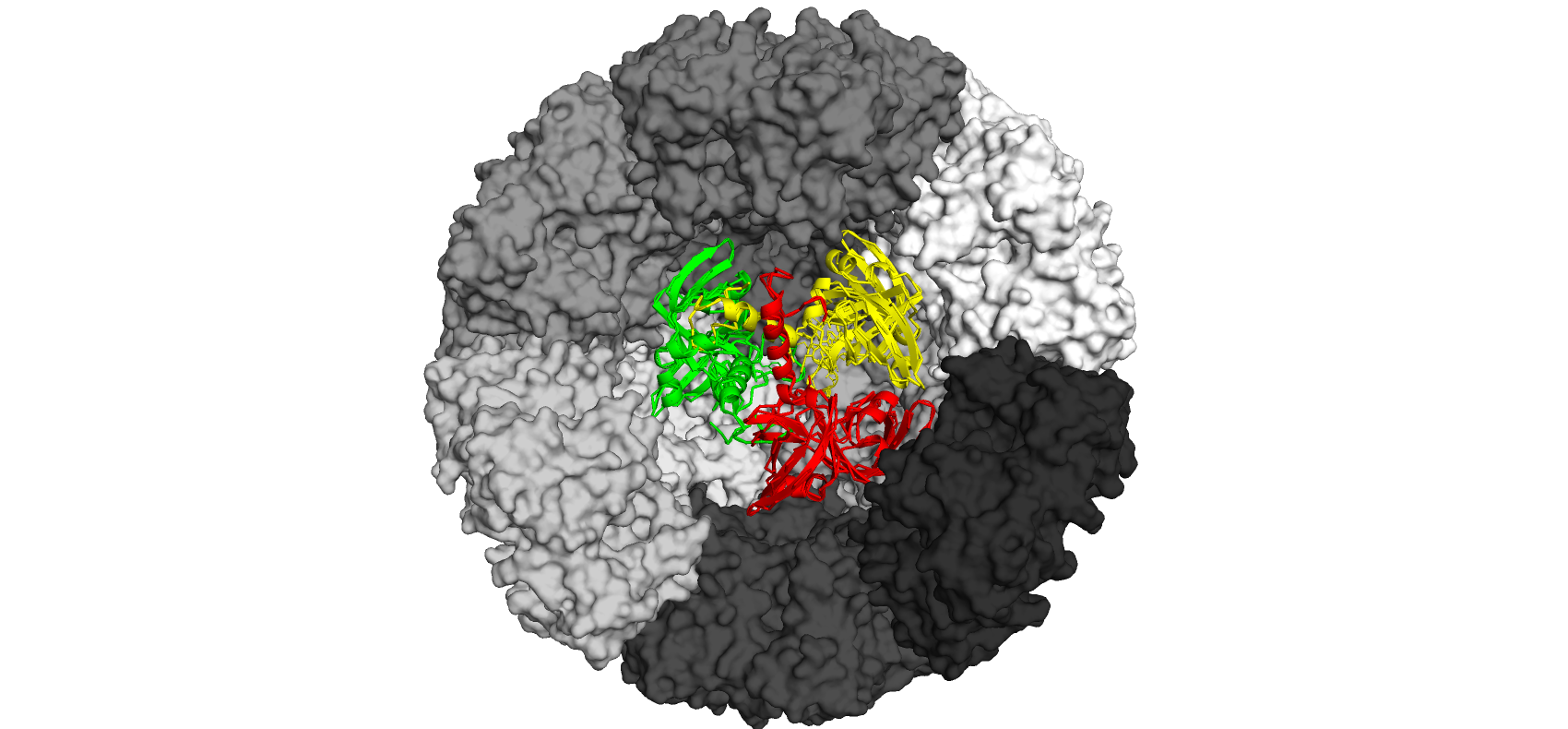


**Figure S2: Model for heavy RF synthase.** This model was created based on [24]. Individual pentamers of RibH are displayed in different shades of grey on their surfaces. Three facets of pentamers have been removed from the original model in order to observe encapsulated RibE which is shown in colored cartoon style. Since no crystal structure for the enzyme from B. subtilis is available, the enzyme has been homology modeled using PHYRE2 [23] with high confidence (≥ 99.9 %), was aligned to the available crystal structure of trimeric RibE from E. coli [25] and manually fitted into the lumen of 60‑meric RibH capsids. The outer diameter of the heavy RF synthase comprises 15.7 nm, whereas the inner diameter accounts for 7.8 nm [26].


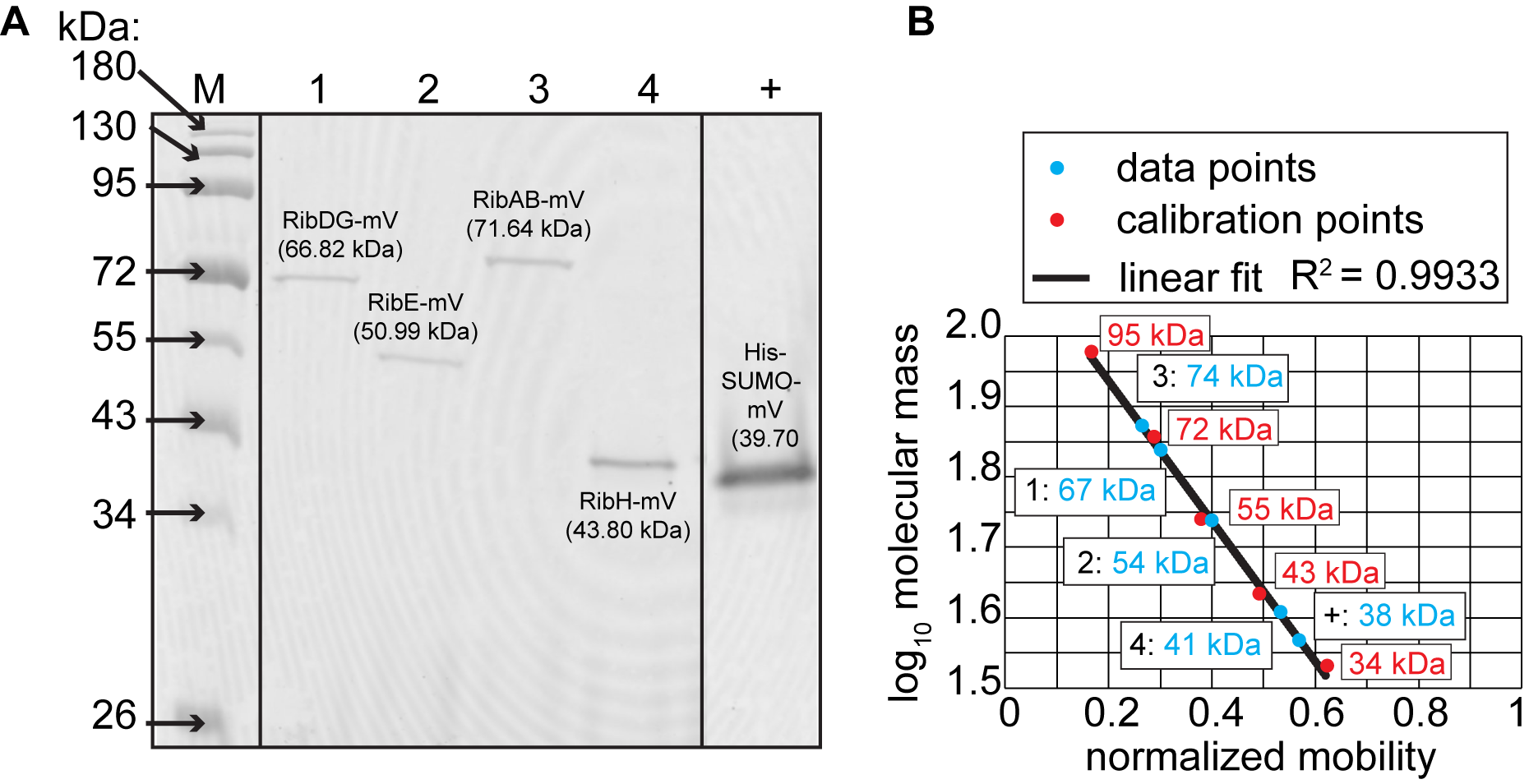


**Figure S3: In‑gel fluorescence detection of fusion enzymes from SDS‑PAGE analysis displaying full length fusion enzymes**. **(A)** The first lane (M) shows MM standard proteins (prestained), the lanes 1 to 4 refer to applied B. subtilis cell lysates from four different strains producing RibDG‑, RibE‑, RibAB‑ and RibH‑mV fusion proteins, respectively. The corresponding MM of each standard protein is indicated on the left. On the right lane (+) a purified His_6_-SUMO-mV FP sample form a frozen lab stock has been applied. Theoretical MM of fusion proteins have been calculated according to their primary sequences and are indicated on top of each fluorescent protein signal **(B)** Semi‑logarithmic plot displaying MM from standard proteins as log_10_ values (lane M; shown as red dots in the plot) used for linear calibration against their relative mobility. MM of fusion proteins and control (shown as light blue dots) have been calculated using the linear fit (R^2^ = 0.9933) and the corresponding linear equation given here in brackets (y $\text{=}\text{ }\text{-}\text{ }\text{0.9948}\text{ }\text{x}\text{ }\text{+}\text{ }\text{2}\text{.}\text{1348}$).


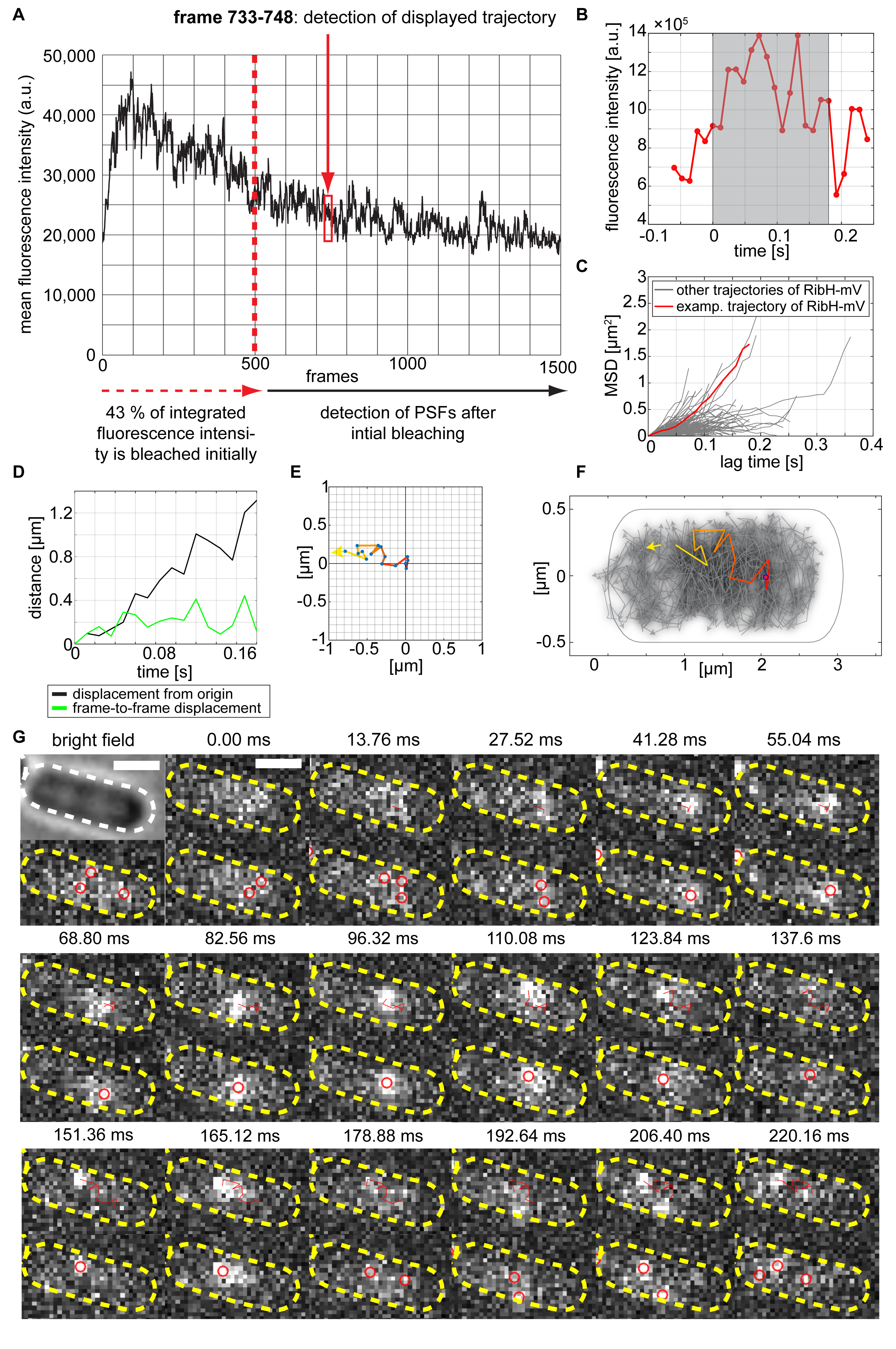


**Figure S4:** **SPT method used to detect and analyze fusion enzymes in live cells of B. subtilis** using FIJI, Oufti, Utrack & SMTracker **(A)** Fluorescence intensity of a particular cell (shown in G) is plotted versus the number of recorded frames (τ = 13.76 ms). Acquired streams were equally cropped for each SPT dataset (indicated here in red after 500 frames) in order to bleach the initial fluorescence yielding low PSF densities that allow reliable SPT. **(B)** Intensity versus time plot for the localized PSF derived from the same trajectory as shown in C, D, E, F and G. **(C)** Individual TAMSD plots of all trajectories from the cell displayed in A and B. The TAMSD curve of the trajectory from A is shown in red. **(D)** Displacement plot showing a comparison between frame‑to‑frame and displacement from origin over time for the same trajectory drawn in A. **(E)** Trajectory from A projected onto a coordinate system for visual step-size analysis. **(F)** Individual trajectories derived from a particular cell are projected onto the cytoplasmic area of a normalized cell and can be selected and analyzed individually. The trajectory from A is highlighted in rainbow colors. **(G)** Consecutive frames of PSF localizations (lower panels with circles) of an exemplary trajectory derived from the fluorescent fusion enzyme RibH‑mV in stream acquisitions of 1000 frames taken with 13.76 ms time intervals (12 ms integration time) and subsequent tracking (upper panels with trajectories) of the particular trajectory shown. Bright field images of cells (top left) are used to draw cell outlines using Oufti software (Paintdakhi A, Parry B, Campos M, Irnov I, Elf J, Surovtsev I, Jacobs-Wagner C: Oufti: an integrated software package for high-accuracy, high-throughput quantitative microscopy analysis. Mol. Microbiol. 2016, 99(4):767-777). Scale bar is shown top left, represents 1 µm and is valid for the entire image sequence.


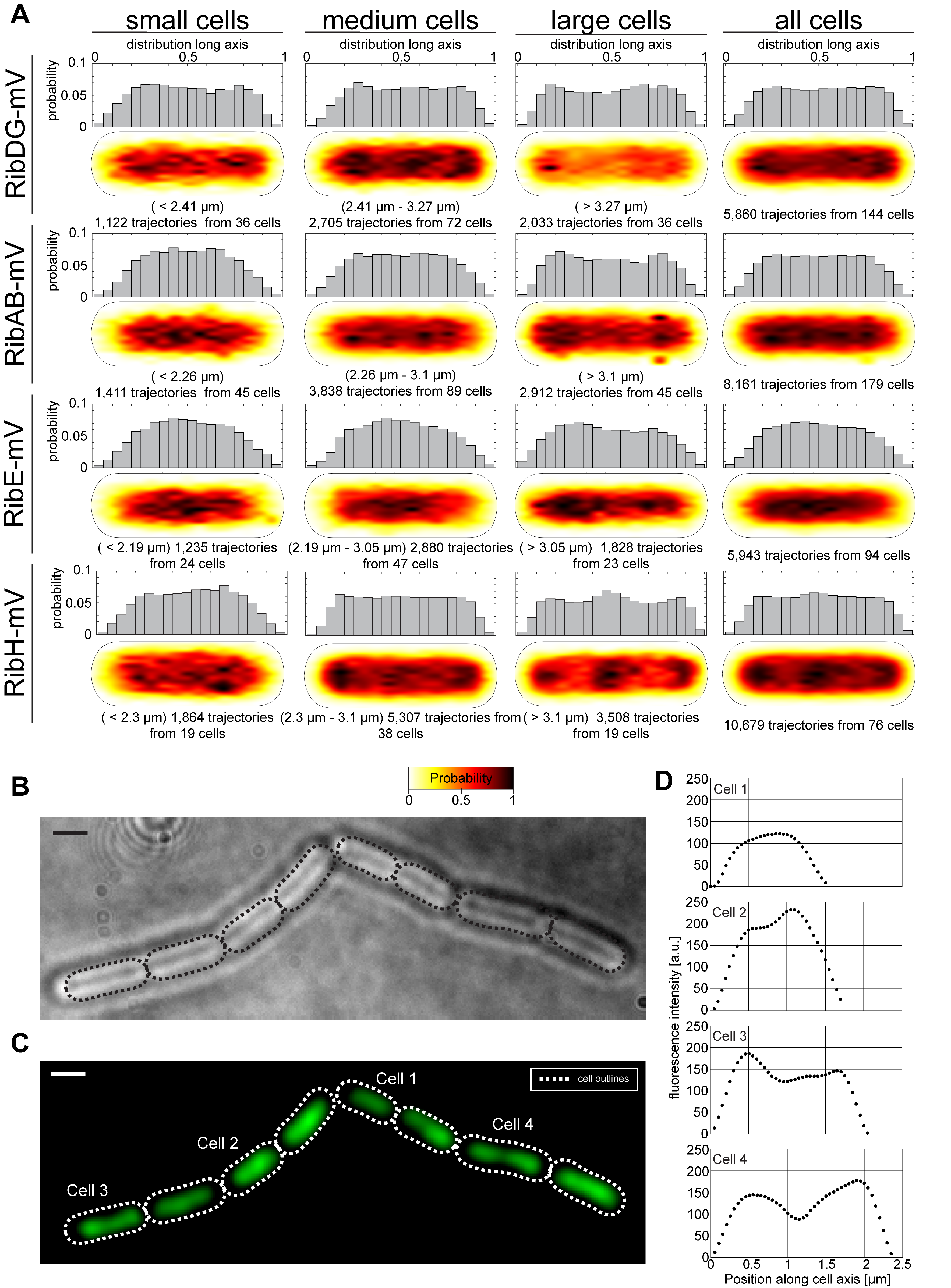


**Figure S5: Spatial distributions of detected spots for all four Rib FP enzymes in a cell size-dependent manner and localization of the nucleoid(s) in exponentially grown cells. (A)** Trajectories were projected into the two dimensional cytoplasmic area of cells divided into three classes yielding small, medium and large cells for further analysis. The likelihood of finding trajectories at a certain place in the cytoplasm is indicated by a color code from white to black (indicated below the heat maps) and further given on top of each cell as a histogram displaying the probability of occurrence along the cells’ long axis. SPT data taken for the spot analysis are summarized in Tab. 1. Signal intensities of shown spatial distribution spot location heat maps for different cell sizes have been normalized with each other. Cell size intervals and the respective number of trajectories that have been considered are indicated on top of each heat map. **(B)** Bright field image of exponentially growing B. subtilis PY79 wt cells. Dashed lines indicate drawn cell outlines, scale bar represents 1 µm. **(C)** Epi fluorescence picture of 4′,6-diamidino-2-phenylindole (DAPI) stained nucleoids with cells from Panel A and cell outlines. **(D)** Intensity profile plots of DAPI stain for four different cells from Panels A and B. Cells and plots have been numbered from small (one nucleoid) to large (two nucleoids), the corresponding cell numbers are indicated in the fluorescence image, as well as, in the corresponding plots. Line plots have been drawn centered along the long axis of each cell.


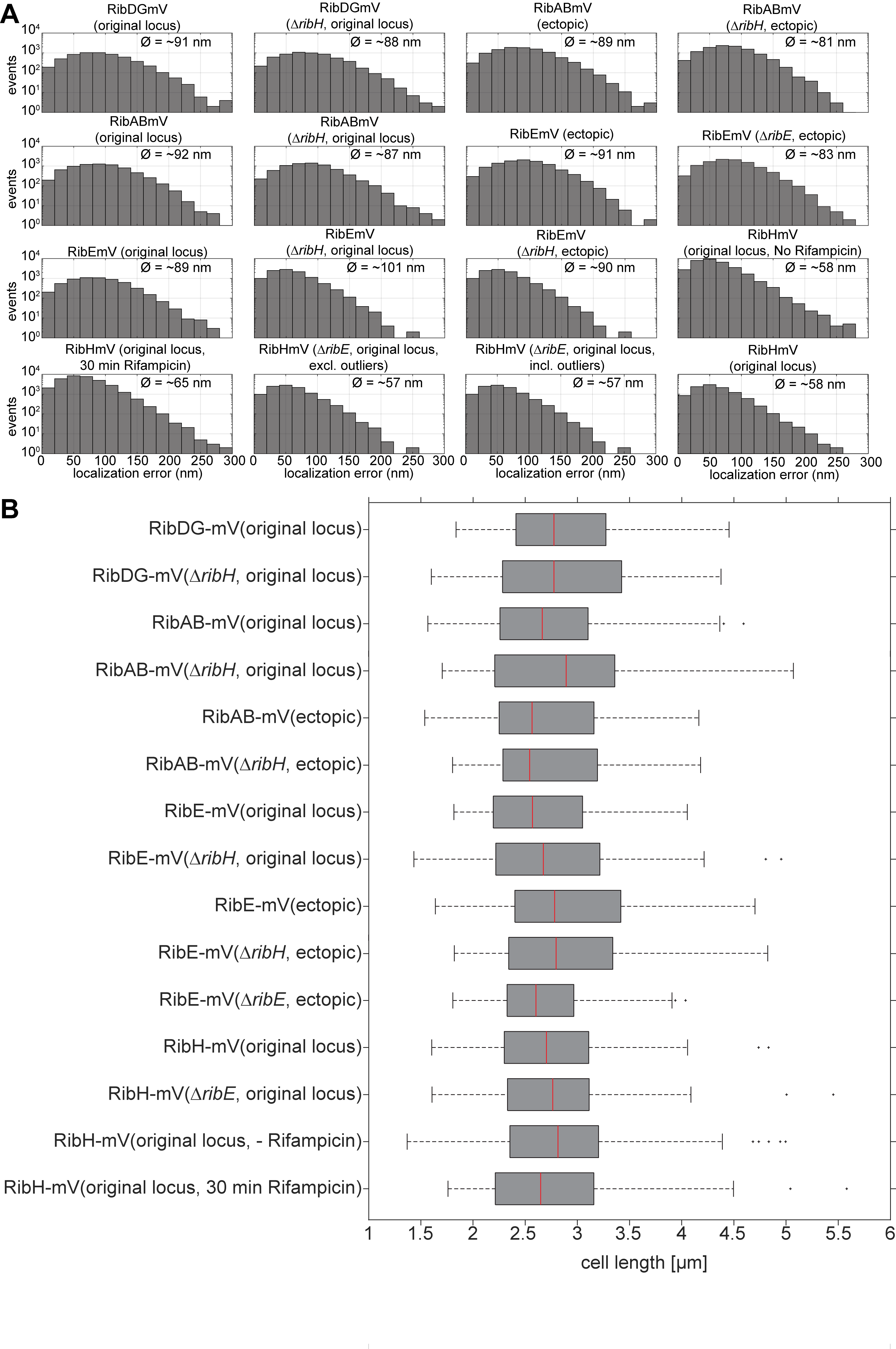


**Figure S6: Localization error histograms and cell size boxplots for all SPT datasets in this study (A)** Localization error histogram for all SPT datasets analyzed. The mean localization error is indicated within each plot. **(B)** Cell size boxplots including all cells analyzed for the particular SPT datasets. Outliers are shown as asterisk.


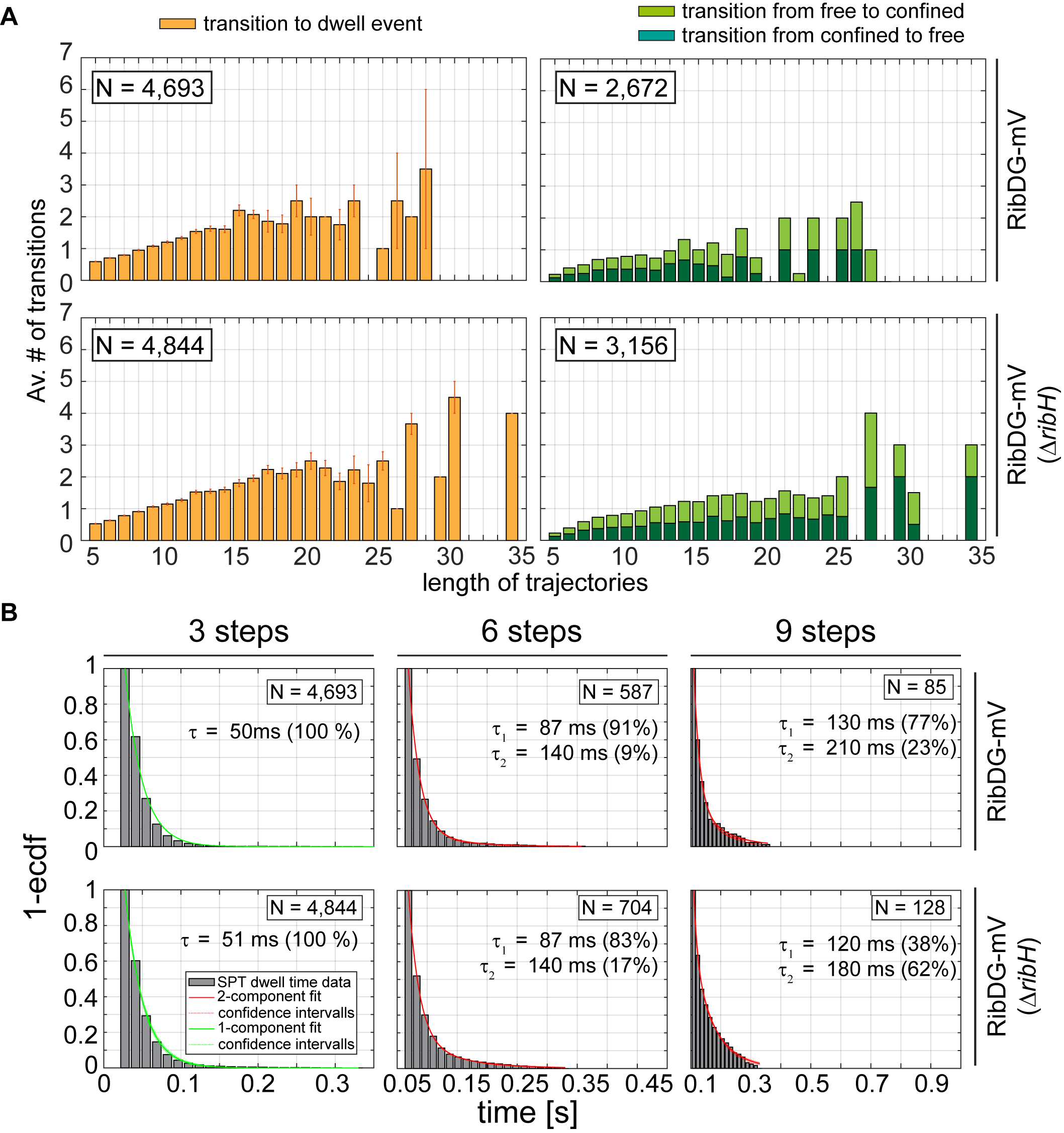


**Figure S7: Transition analysis of RibDG‑mV in the absence and presence of RibH and dwell-times for different numbers of confined steps. (A)**Transitions of trajectories for RibDG‑mV showing mixed behavior from free diffusion to dwell events and confinement (and *vice versa*) in dependency of trajectories length. The number of dwell events detected is given within the respective plots. **(B)** Dwell time empirical histograms for RibDG‑MV for different numbers of steps considered for dwelling. The number of events detected is given within the respective plots. Histograms have been fitted using either mono-exponential decay or bi-exponential decay functions. The resulting dwell times are given within the respective plots and are further listed in table S1.

**
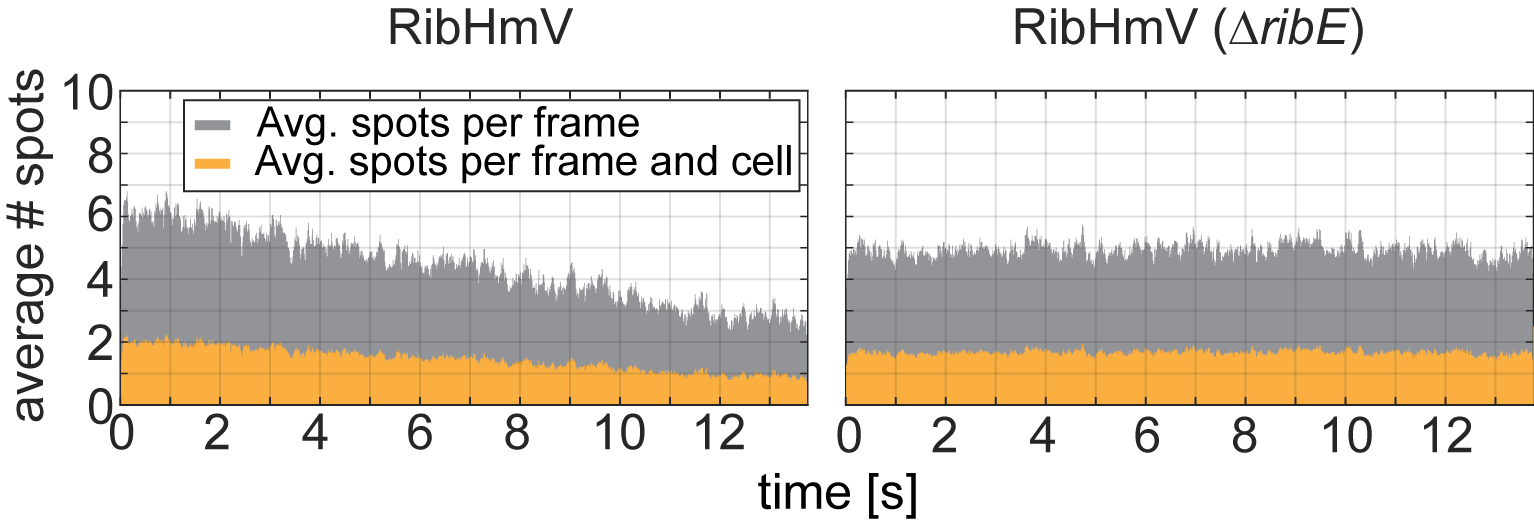
 Figure S8: Spots detected per frame (in gray) and per frame and cell (in orange) for RibH‑mV in the presence (left) and absence (right) of *ribE.*** Data in grey represents the average number of spots per frame and data in orange represents the average number of spots per cell.


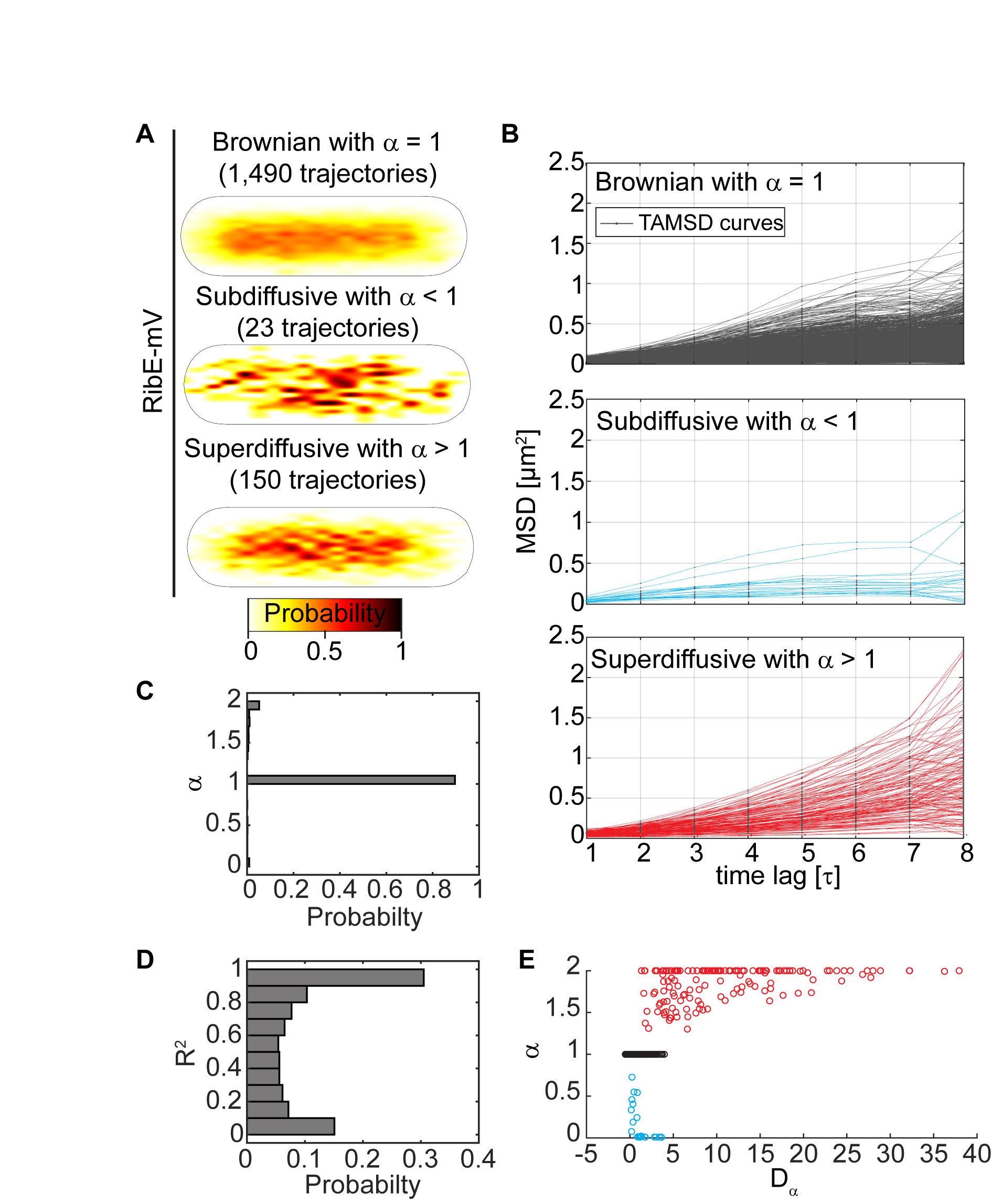


**Figure S9: RibE shows different modes of diffusion in the bacterial cytoplasm.** **(A)** Spatial distribution heat maps for different modes of diffusion of RibE‑mV from an SPT dataset summarized in table 2. Heat maps have been normalized with each other. **(B)** TAMSD plots of trajectories from RibE‑mV displaying the different modes of diffusion. Shown are trajectories with R^2^‑values that are at least 0.9 or higher **(C)** Probability-plot showing the normalized probability of finding exponent α in our RibE‑mV SPT dataset. **(D)** Probability-plot displaying the normalized probability of R^2^‑values for individual fits of all trajectories from the RibE‑mV dataset. **(E)** Distribution of exponent alpha in dependence from its generalized diffusion coefficient. Colors are the same as in B.

**
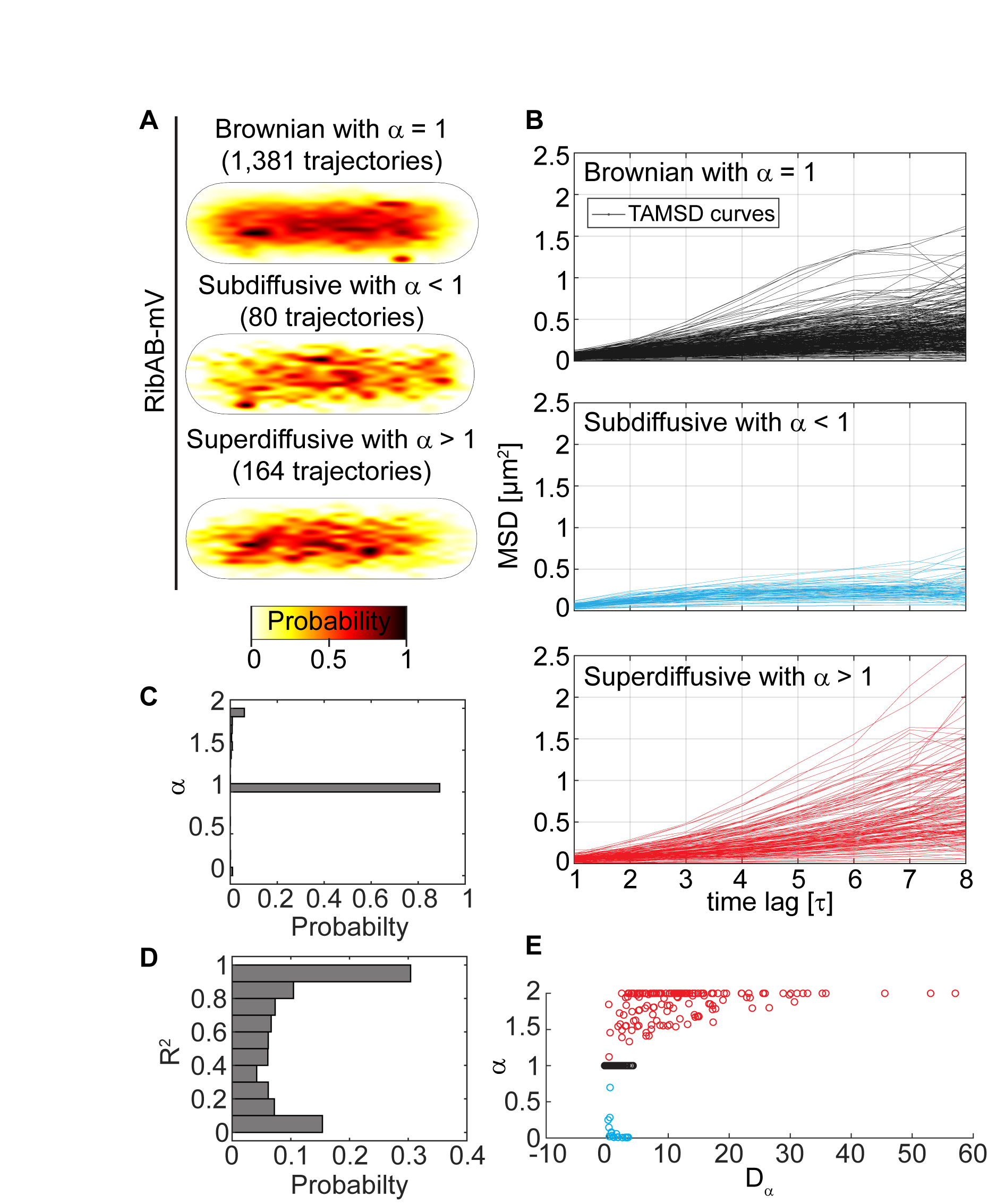
**

**Figure S10: RibAB shows different modes of diffusion in the bacterial cytoplasm.** **(A)** Spatial distribution heat maps for different modes of diffusion of RibAB‑mV from an SPT dataset summarized in table 2. Heat maps have been normalized with each other. **(B)** TAMSD plots of trajectories from RibAB‑mV displaying the different modes of diffusion. Shown are trajectories with R^2^‑values that are at least 0.9 or higher **(C)** Probability-plot showing the normalized probability of finding exponent α in our RibAB‑mV SPT dataset. **(D)** Probability-plot displaying the normalized probability of R^2^‑values for individual fits of all trajectories from the RibE‑mV dataset. **(E)** Distribution of exponent alpha in dependence from its generalized diffusion coefficient. Colors are the same as in (B).


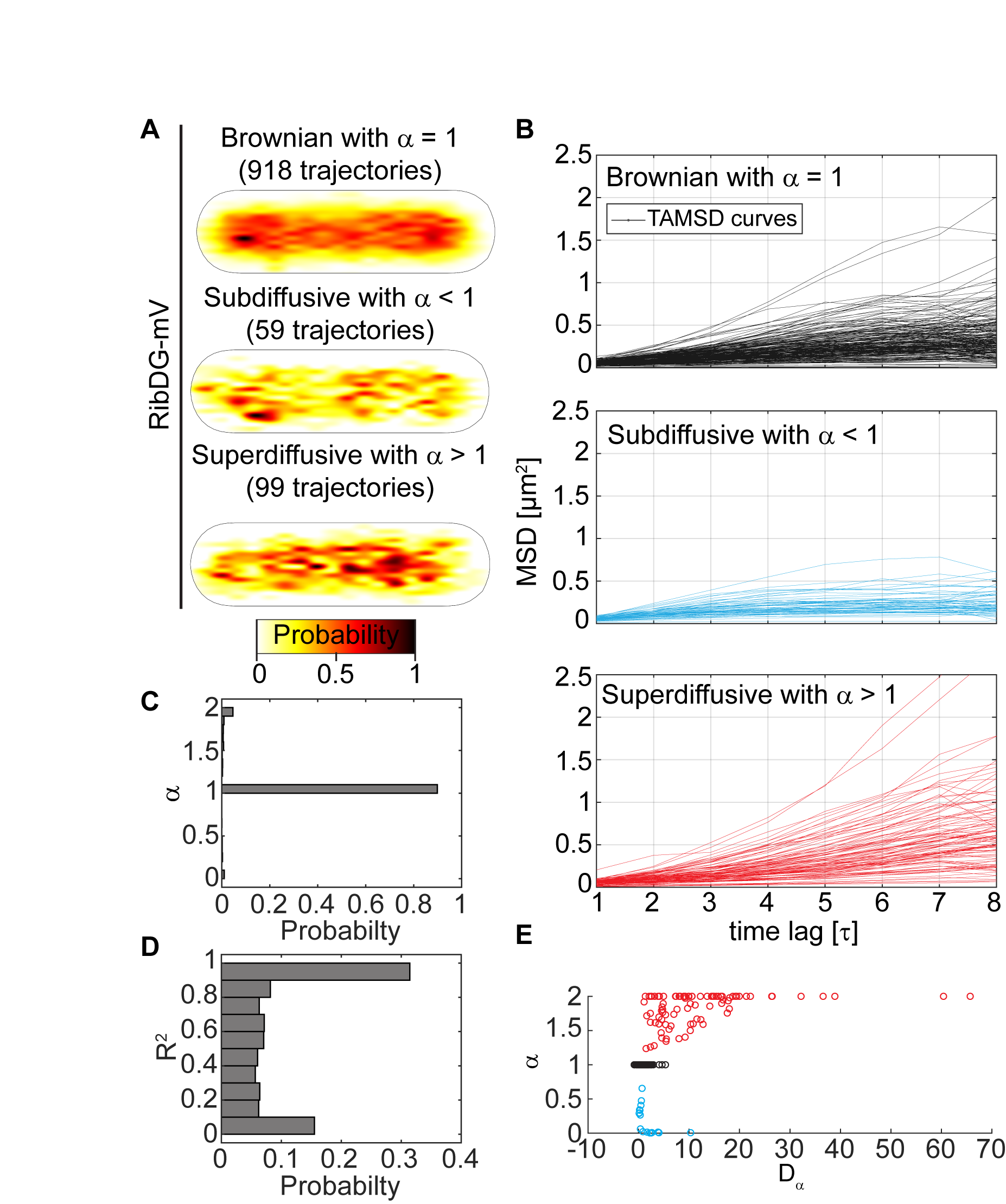


**Figure S11: RibDG shows different modes of diffusion in the bacterial cytoplasm.** **(A)** Spatial distribution heat maps for different modes of diffusion of RibDG‑mV from an SPT dataset summarized in table 2. Heat maps have been normalized with each other. **(B)** TAMSD plots of trajectories from RibDG‑mV displaying the different modes of diffusion. Shown are trajectories with R^2^‑values that are at least 0.9 or higher **(C)** Probability-plot showing the normalized probability of finding exponent α in our RibDG‑mV SPT dataset. **(D)** Probability-plot displaying the normalized probability of R^2^‑values for individual fits of all trajectories from the RibDG‑mV dataset. **(E)** Distribution of exponent alpha in dependence from its generalized diffusion coefficient. Colors are the same as in (B).

**
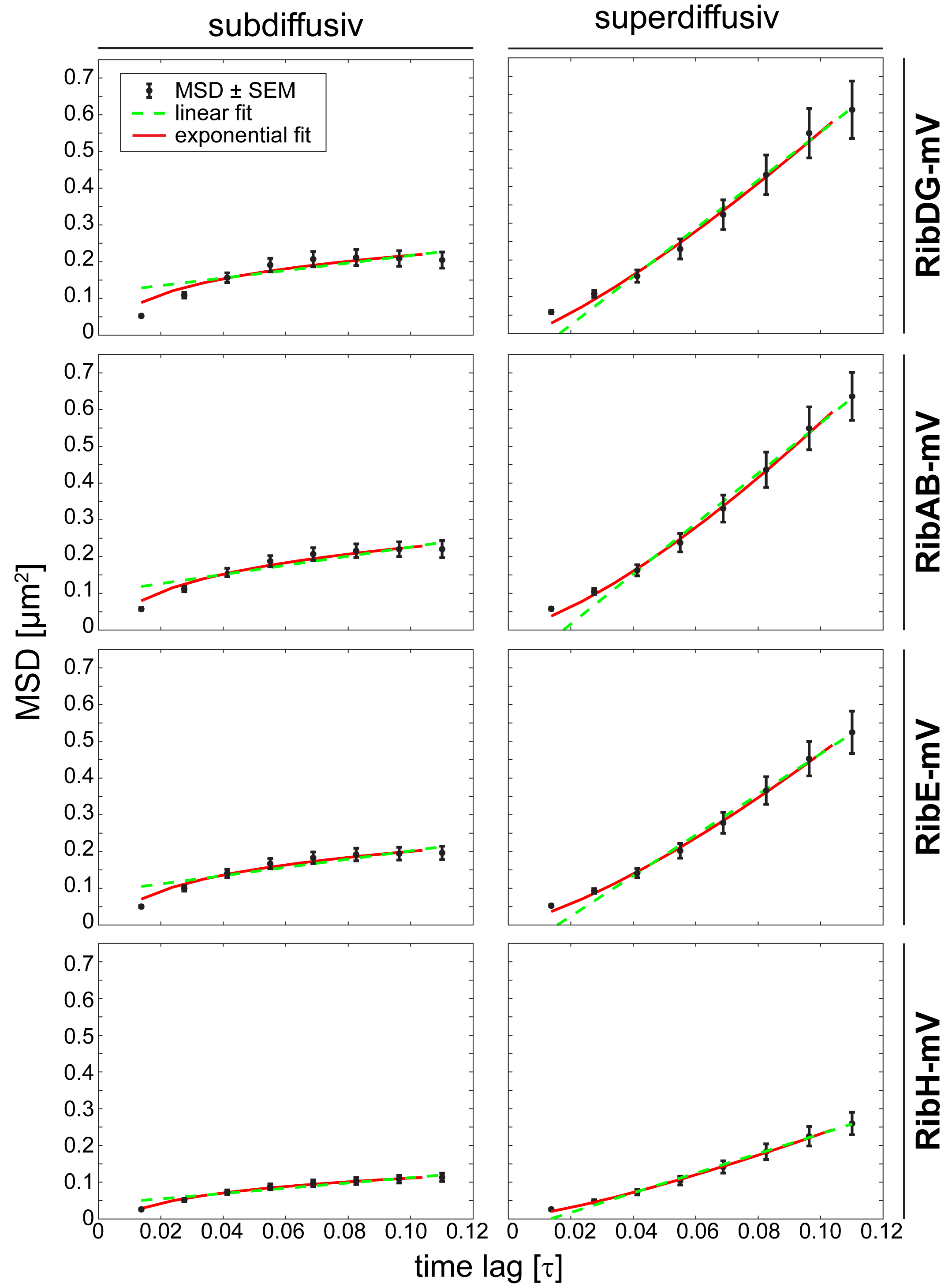
**

| **Dataset** | **p-value (subdiffusive)** | **p-value (superdiffusive)** |
| --- | --- | --- |
| RibDG-mV | 0.07002 | 0.213 (rejected) |
| RibAB‑mV | 0.02345 | 0.02007 |
| RibE‑mV | 0.0186 | 0.02257 |
| RibH‑mV | 0.001287 | 0.01812 |

**Fig. S12: EAMSD plots for trajectories classified as anomalous (either sub‑ or superdiffusive) of all four Rib FP enzymes.** Dashed green lines represent a linear fit assuming a Brownian model, and solid red an exponential fit assuming subdiffusion (left) or superdiffusion (right). An F-test for nested models has been applied to determine if they diffuse significantly anomalously (table S4). The hypothesis of superdiffusion was rejected only in case of RibDG-mV, in all other cases supported (see table).

**Table S1:** List of diffusion coefficients derived from structural data which have been calculated using Hydropro software [23].

| Enzyme (organism) | Oligomeric state | Diffusion in H_2_O at 25°C [µm^2^/s]  (0.001 Pa × s) | Diffusion in cytoplasm at 25°C [µm^2^/s]  (0.0549 Pa × s) | ln (D_0_/D_JD_)  [η] | Structure derived from protein databank file  [reference] | % identity with *B. subtilis* aa sequence |
| --- | --- | --- | --- | --- | --- | --- |
| **RibDG** (*B. subtilis*) | Tetramer | 43.35 | 0.7895 | 3.603  [0.037 Pa × s] | DOI:  [10.2210/pdb3EX8/pdb](http://doi.org/10.2210/pdb3EX8/pdb)  [38] | - |
| **RibA2** (*M. tubercu-losis*) | Dimer | 58.71 | 1.069 | 3.950  [0.049 Pa × s] | DOI: [10.2210/pdb4I14/pdb](http://doi.org/10.2210/pdb4I14/pdb) [39] | 69.85% |
| **RibE** (*E. coli*) | Trimer | 63.12 | 1.15 | 4.031  [0.056 Pa × s] | **DOI:**  [10.2210/pdb1I8D/pdb](http://doi.org/10.2210/pdb1I8D/pdb)  [25] | 45.89 % |
| **RibH** (*B. subtilis*) | Pentamer | 60.84 | 1.108 | 4.098  [0.060 Pa × s] | **DOI:** [10.2210/pdb1RVV/pdb](http://doi.org/10.2210/pdb1RVV/pdb)  [24] | - |
| **RibH** (*B. subtilis*) | 60-mer | 31.30 | 0.57 | 4.437 [0.061 Pa × s] | **DOI:** [10.2210/pdb1RVV/pdb](http://doi.org/10.2210/pdb1RVV/pdb)  [24] | - |

**Table S2:** Mean estimated localization errors and radii of confinement for each SPT dataset used in this study

| **Data set** | **Trajectories (minimum 5 steps)** | **Estimated localization error from TAMSD y-axes interception (nm)** | **Resulting radius of confinement used for analysis (nm) error*2.5** |
| --- | --- | --- | --- |
| **RibDG-mV (original locus)** | 5,936 | 91.09 | 228 |
| **RibDG-mV (Δ*rib*H, original locus)** | 5,918 | 87.57 | 219 |
| **RibAB-mV (original locus)** | 8,161 | 91.76 | 229 |
| **RibAB-mV (Δ*ribH*, original locus)** | 7,262 | 87.53 | 219 |
| **RibH-mV (original locus)** | 10,679 | 58.1 | 145 |
| **RibH-mV (Δ*rib*E, original locus, incl. outliers)** | 10,637 | 56.91 | 142 |
| **RibH-mV (Δ*rib*E, original locus, excl. outliers)** | 10,622 | 56.98 | 142 |
| **RibE-mV (original locus)** | 5,943 | 86.61 | 217 |
| **RibE-mV (Δ*ribH*, original locus)** | 5,868 | 100.70 | 252 |
| **RibE-mV (ectopic)** | 10,690 | 91.32 | 228 |
| **RibE-mV (Δ*ribH*, ectopic)** | 10,265 | 89.98 | 225 |
| **RibE-mV (Δ*ribE*, ectopic)** | 10,612 | 82.82 | 207 |
| **RibAB-mV (ectopic)** | 10,342 | 88.78 | 222 |
| **RibAB-mV (Δ*ribH*, ectopic)** | 11,186 | 80.67 | 202 |
| **RibH-mV (control)** | 33,258 | 56.70 | 142 |
| **RibH-mV (30 min Rifampicin)** | 34,352 | 65.41 | 164 |

**Table S3:** Summary of confinement analysis from all SPT data used in this study. Datasets

are the same as in Tab. 2. For all analysis shown in here either three (a), six (b) or nine (c)

steps of confinement were used.

| **Strain**  **(R of confinement)** | **Static** | **Mobile** | **% trajectories**  **free vs. mixed**  **(Step distribution: free vs. confined)** | **Dwell time** (1-comp. fit)  ± uncertainty | **Dwell times** (2-comp. fit)  ± uncertainty | **Transitions to dwell event (% of trajectories with three, six or nine steps)** | **Transitions from freely diffusive to confined and *vice versa* (% of trajectories with three, six or nine steps** |
| --- | --- | --- | --- | --- | --- | --- | --- |
| **RibDG-mV**  **(228 nm)** | a) 20.9%  b) 4.4%  c) 0.9% | a) 79.1%  b) 95.6%  c) 99.1% | a) 28.1% -51%  (40% - 60%)  b) 90.2 – 5.4%  (88% - 12%)  c) 98.6% - 0.5%  (97.4% - 2.6%) | a) 50 ± 1.3 ms (100%) | b) τ_1_: 87 ± 0.6 ms (91 ± 3.2%)  τ_2_: 140 ± 18 ms  (8.6 ± 3.2%)  c) τ_1_: 130 ± 3 ms (77 ± 9.7%)  τ_2_: 210 ± 32 ms  (23 ± 9.7%) | a) 4,693 (79.0%)  b) 587 (16.5%)  c) 85 (8.9%) | a) 2,672 (45%)  b) 270 (7.6%)  c) 27 (2.8%) |
| **RibDG-mV, Δ*ribH***  **(219 nm)** | a) 16.4%  b) 4.1%  c) 0.9% | a) 83.6%  b) 95.9%  c) 99.1% | a) 29.7% - 53.9%  (42% - 58%)  b) 88.4% - 7.5% (86%-14%)  c) 97.8% - 1.3% (96.2% - 3.8%) | a) 51 ± 1.6 ms (100%) | b) τ_1_:87 ± 1 ms (83 ± 4.3%)  τ_2_:140 ± 12 ms (17 ± 4.3%)  c) τ_1_: 120 ± 4.3 ms (37 ± 8.8%)  τ_2_: 180 ± 7.9 ms (63 ± 8.8%) | a) 4,844 (81.8%)  b) 704 (17.7%)  c) 128 (9.3%) | a) 3,156 (53.3%)  b) 391 (9.8%)  c) 68 (4.9%) |
| **RibAB-mV**  **(229 nm)** | a) 18.7%  b) 4.4%  c) 1% | a) 81.3%  b) 95.6%  c) 99% | a)26.9% - 54.4%  (40% - 60%)  b)87.9% - 7.6% (86% - 14%)  c)98.1% - 0.9%  (96.3% - 3.7%) | a) 51 ± 0.77 ms (100%) | b) τ_1_: 86 ± 0.2 ms (90 ± 0.53%)  τ_2_: 190 ± 5.7 ms (9.6 ± 0.53%)  c) τ_1_: 130 ± 1.3 ms (56 ± 2.3%)  τ_2_: 230 ± 5.5 ms (44 ± 2.3%) | a) 6,879 (84.3%)  b) 1,005 (18.4%)  c) 156 (8.5%) | a) 4,259 (52.2%)  b) 562 (10.3%)  c) 61 (3.3%) |
| **RibAB‑mV*,* Δ*ribH***  **(219 nm)** | a) 17.2%  b) 4.3%  c) 0.6% | a) 82.8%  b) 95.7%  c) 99.4% | a)21.4% - 61.4% (36% - 64%)  b)83.7% - 12.1%  (82% - 18%)  c) 97.4% - 2%)  (96.1% - 3.9%) | a) 53 ± 1.3 ms (100%) | b) τ_1_: 89 ± 0.7 ms (96 ± 3.1%)  τ_2_: 150 ± 38 ms (4.3 ± 3.1%)  c) τ_1_: 150 ± 3.5 ms (58 ± 7.2%)  τ_2_:120 ± 2.1 ms (42 ± 7.2%) | a) 6,992 (96.3%)  b) 1,241 (23.2%)  c) 193 (8.7%) | a) 4,358 (60.0%)  b) 820 (15.3%)  c) 112 (5.0%) |
| **RibAB-mV, ectopic**  **(222 nm)** | a) 17.2%  b) 4%  c) 0.8% | a) 82.8%  b) 96%  c) 99.2% | a)27.9% - 54.8%  (41% - 59%)  b)88.1% - 7.9%  (86% - 14%)  c)98% - 1.2%  (96.4% - 3.6%) | a) 51 ± 0.64 ms (100%) | b) τ_1_:87 ± 0.33ms (86 ± 1.6%)  τ_2_:140 ± 4.6ms (14 ± 1.6%)  c) τ_1_:120 ± 3.1 ms (39 ± 7.9%)  τ_2_:170 ± 2.1 ms (61 ± 7.9%) | a) 8,734 (84.4%)  b) 1,263 (18.1%)  c) 214 (8.6%) | a) 5,558 (53.7%)  b) 760 (10.9%)  c) 95 (3.8%) |
| **RibAB-mV, ectopic, Δ*ribH***  **(202 nm)** | a) 13.8%  b) 3.4%  c) 0.5% | a) 86.2%  b) 96.6%  c) 99.5% | a) 22.3% - 63.9%  (38% - 62%)  b) 84% - 12.6%  (84% - 16%)  c) 97.5% - 2%  (96.6% -3.4%) | a) 52 ± 1.1 ms (100%) | b) τ_1_: 90 ± 0.44 ms (98 ± 2%)  τ_2_: 160 ± 73 ms (1.7 ± 2%)  c) τ_1_: 130 ± 2.7 ms (81 ± 19%)  τ_2_: 160 ± 22 ms (19 ± 19%) | a) 11,310 (101.1%)  b) 1,896 (22.5%)  c) 284 (7.0%) | a) 7,590 (67.8%)  b) 1,438 (17.0%)  c) 203 (5.0%) |
| **RibE-mV**  **(217 nm)** | a) 16.9%  b) 5%  c) 1.2% | a) 83.1%  b) 95%  c) 98.8% | a) 25.6% - 57.5%  (39% - 61%)  b) 85% - 9.9%  (83% - 17%)  c) 97.3% - 1.5%  (95.7% - 4.3%) | a) 53 ± 0.9 ms (100%) | b) τ_1_: 87 ± 1.2 ms (76 ± 8.4%)  τ_2_: 110 ± 6.5 ms (24 ± 8.4%)  c) τ_1_: 140 ± 1.5 ms (91 ± 5.4%)  τ_2_: 220 ± 42 ms (9.4 ± 5.4%) | a) 5,322 (89.5%)  b) 922 (22.2%)  c) 163 (9.8%) | a) 3,328 (56.0%)  b) 556 (13.4%)  c) 80 (4.8%) |
| **RibE mV, Δ*ribH***  **(252 nm)** | a) 23.8%  b) 4.8%  c) 0.7% | a) 76.2%  b) 95.2%  c) 99.3% | a) 26% - 50.2%  (37% - 63%)  b)89.7% - 5.5%  (87% - 13%)  c)98.6% - 0.7%  (97.4% - 2.6%) | a) 50 ± 1.6 ms (100%) | b) τ_1_: 82 ± 1.9 ms (70 ± 14%)  τ_2_: 110 ± 8.5 ms (30 ± 14%)  c) τ_1_: 120 ± 12 ms (35 ± 34%)  τ_2_: 160 ± 15 ms (65 ± 34%) | a) 4,775 (81.4%)  b) 616 (17.9%)  c) 86 (10.1%) | a) 2,535 (43.2%)  b) 268 (7.8%)  c) 29 (3.4%) |
| **RibE-mV, ectopic**  **(228 nm)** | a) 19.4%  b) 4.9%  c) 0.9% | a) 80.6%  b) 95.1%  c) 99.1% | a) 21.3% - 59.3%  (35% - 65%)  b) 84.1% - 11% (82% - 18%)  c) 97.1% - 2%  (95.5% - 4.5%) | a) 53 ± 1 ms (100%) | b) τ_1_: 82 ± 1.9 ms (70 ± 14%)  τ_2_: 110 ± 8.5 ms (30 ± 14%)  c) τ_1_: 140 ± 1.9 ms (83 ± 9.9%)  τ_2_: 180 ± 16 ms (17 ± 9.9%) | a) 10,208 (95.5%)  b) 1,763 (23.0%)  c) 312 (10.3%) | a) 6,060 (56.7%)  b) 1,077 (14.0%)  c) 191 (6.3%) |
| **RibE‑mV, ectopic, Δ*ribH***  **(225 nm)** | a) 18.1%  b) 4.4%  c) 0.5% | a) 81.9%  b) 95.6%  c) 99.5% | a) 21.1% - 60.8%  (35% - 65%)  b) 83.6% - 11.9%  (82% - 18%)  c) 97.5% - 2%  (96.3% - 3.7%) | a) 53 ± 1.2 ms (100%) | b) τ_1_: 90 ± 0.5 ms (98 ± 2.1%)  τ_2_: 150 ± 73 ms (1.6 ± 2.1%)  c) τ_1_:130 ± 3.1 ms (91 ± 17%)  τ_2_:180 ± 71 ms (8.8 ± 17%) | a) 9,882 (96.2%)  b) 1,734 (23.4%)  c) 257 (8.8%) | a) 5,837 (56.8%)  b) 1,111 (15.0%)  c) 165 (5.6%) |
| **RibE‑mV, ectopic, Δ*ribE***  **(207 nm)** | a) 16.1%  b) 3.9%  c) 0.6% | a) 83.9%  b) 96.1%  c) 99.4% | a) 22.1% - 61.8%  (36% - 64%)  b) 83.6% - 12.5%  (83% -17%)  c) 97.2% - 2.2%  (96% - 4%) | a) 53 ± 1.2 ms (100%)  c) 140 ± 0.6 ms (100%) | b) τ_1_: 91 ± 0.7 ms (98 ±3.9%)  τ_2_: 140 ± 69 ms (2.1 ± 3.9%) | a) 10,625 (100.1%)  b) 1,842 (23.3%)  c) 304 (8.7%) | a) 6,452 (60.8%)  b) 1,310 (16.7%)  c) 233 (6.6%) |
| **RibH-mV**  **(145 nm)** | a) 11.6%  b) 3.6%  c) 0.9% | a) 88.4%  b) 96.4%  c) 99.1% | a) 23.4% - 65%  (39% - 61%)  b) 78.3% - 18.1%  (79% - 21%)  c) 94.7% - 4.4%  (93.2% - 6.8%) | a) 56 ± 0.5 ms (100%) | b) τ_1_: 160 ± 26 ms (5.7 ± 2.9%)  τ_2_: 95 ± 0.7 ms (94 ± 2.9%)  c) τ_1_: 130 ± 1.6 ms (68 ± 7%)  τ_2_: 170 ± 6.3 ms (32 ± 7%) | a) 12,062 (113%)  b) 2,661 (30.8%)  c) 606 (12.3%) | a) 8,450 (79.1%)  b) 2,161 (25.0%)  c) 448 (9.1%) |
| **RibH‑mV, Δ*ribE,* incl. outliers**  **(142 nm)** | a) 16.2%  b) 6.5%  c) 2.4% | a) 83.8%  b) 93.5%  c) 97.6% | a) 19.1% - 64.7%  (28% - 72%)  b) 69.9% - 23.6%  (60% - 40%)  c) 88.4% - 9.3%  (74% - 26%) | - | a) τ_1_: 62 ± 0.2 ms (96 ± 0.3%)  τ_2_: 330 ± 22 ms (3.7 ± 0.3%)  b) τ_1_: 110 ± 0.5 ms (91 ± 0.6%)  τ_2_: 400 ± 19 ms (9.3 ± 0.6%)  c) τ_1_: 170 ± 0.9 ms (94 ± 0.3%)  τ_2_: 1500 ± 86 ms (6.3 ± 0.3%) | a) 13,106 (123.2%)  b) 3,986 (46.5%)  c) 1,521 (29.8%) | a) 8,137 (76.5%)  b) 2,728 (31.8%)  c) 893 (17.5%) |
| **RibH‑mV, Δ*ribE*; excl. outliers**  **(142 nm)** | a) 16.2%  b) 6.4%  c) 2.3% | a) 83.8%  b) 93.6%  c) 97.7% | a) 19.1% - 64.7%  (31% - 69%)  b) 70% - 23.6%  (66% - 34%)  c) 88.5% - 9.2%  (81% - 19%) | - | a) τ_1_: 61 ± 0.5 ms (96 ± 0.9%)  τ_2_: 220 ± 30 ms (4.5 ± 0.9%)  b) τ_1_: 100 ± 0.6 ms (82 ± 1.2%)  τ_2_: 220 ± 70 ms (18 ± 1.2%)  c) τ_1_: 160 ± 1.1 ms (82 ± 1.4%)  τ_2_: 350 ± 14 ms (18 ± 1.4%) | a) 13,037 (122.7%)  b) 3,925 (45.5%)  c) 1,463 (28.8%) | a) 8,135 (76.5%)  b) 2,726 (31.6%)  c) 893 (17.6%) |
| **RibH‑mV**  **(no drug)**  **(142 nm)** | a) 12.3%  b) 3.9%  c) 1% | a) 87.7%  b) 96.1%  c) 99% | a) 22.5% - 65.2%  (38% - 62%)  b) 77.4% - 18.7%  (78% - 22%)  c) 93.9% - 5.1%  (92% - 8%) | a) τ: 57 ± 0.6 ms (100%) | b) τ_1_: 95 ± 0.3 ms (87 ± 1.6%)  τ_2_: 150 ± 4.7 ms (13 ± 1.6%)  c) τ_1_: 140 ± 0.8 ms (84 ± 2.5%)  τ_2_: 210 ± 9.3 ms (16 ± 2.5%) | a) 38,612 (115.6%)  b) 8,734 (31.9%)  c) 2,202 (14.7%) | a) 26,564 (79.6%)  b) 7,006 (25.6%)  c) 1,637 (11.0%) |
| **RibH‑mV**  **(30 min drug)**  **(164 nm)** | a) 11.2%  b) 3.2%  c) 0.7% | a) 88.8%  b) 96.8%  c) 99.3% | a) 24.5% - 64.2% (41% - 59%)  b) 82% - 14.8% (82% - 18%)  c) 96.3% - 3% (94.9% - 5.1%) | a) τ: 53 ± 0.4 ms (100%) | b) τ_1_: 94 ± 0.5 ms (86 ± 1.5%)  τ_2_:140 ± 3.6 ms (14 ± 1.5%)  c) τ_1_: 140 ± 0.6 ms (85 ± 1.9%)  τ_2_: 220 ± 8.3 ms (15 ± 1.9%) | a) 36,670 (106.7%)  b) 6,820 (24.9%)  c)1,344 (9.0%) | a) 26,907 (78.3%)  b) 5,700 (20.8%)  c) 984 (6.6%) |

**Table S4**: Strains used in this study

| **Strain:** | **Relevant genotype:** | **Source or reference:** |
| --- | --- | --- |
| *E. coli* XL-1 Blue | *recA1 endA1 gyrA96 thi-1 hsdR17 supE44 relA1 lac* [F ́ *proABlacIqZ∆M15* Tn*10* (Tet^r^)] | Stratagene |
| *B. subtilis* PY79 | Prototrophic derivative of *B. subtilis*  168 | Laboratory stock |
| *B. subtilis* 168 *ΔribH* | *trpC2*; *ΔribH::kan* (Kan^r^) | BGSC: BKK23250 |
| *B. subtilis* 168 *ΔribE* | *trpC2*; *ΔribE::kan* (Kan^r^) | BGSC: BKK23270 |
| **Derivatives of PY79 (transformed with plasmids):** | | |
| PY79 | *ribDG‑mVenus* *cat* (Cm^r^) | This study |
| PY79 | *ribE‑mVenus* *cat* (Cm^r^) | This study |
| PY79 | *ribAB‑mVenus* *cat* (Cm^r^) | This study |
| PY79 | *ribH‑mVenus* *cat* (Cm^r^) | This study |
| PY79 | *amyE::P_Xyl_-ribE-mVenus spc* (Spec^r^) | This study |
| PY79 | *amyE::P_Xyl_-ribAB-mVenus spc* (Spec^r^) | This study |
| **Derivatives of PY79 (transformed with chromosomal DNA of BKK23250 or BK23270):** | | |
| PY79 *ΔribH* | *ΔribH::kan* (Kan^r^) | This study |
| PY79 *ΔribE* | *ΔribE::kan* (Kan^r^) | This study |
| **Derivatives of PY79 *ΔribH*:** | | |
| PY79 *ΔribH:ribE-mVenus* | *ΔribH::kan:ribE‑mVenus cat* (Kan^r^, Cm^r^) | This study |
| PY79 *ΔribH:ribAB-mVenus* | *ΔribH::kan:ribAB‑mVenus cat* (Kan^r^, Cm^r^) | This study |
| PY79 *ΔribH:amyE::P_Xyl_ribE-mVenus* | *ΔribH::kan, amyE::P_Xyl_ribE‑mVenus spc* (Kan^r^, Spec^r^) | This study |
| PY79 *ΔribH::amyE::P_Xyl_ribAB-mVenus* | *ΔribH::kan, amyE::P_Xyl_ribAB‑mVenus spc* (Kan^r^, Spec^r^) | This study |
| **Derivatives of PY79 *ΔribE*:** | | |
| PY79 *ΔribE:ribH-mVenus* | *ΔribE::kan:ribH‑mVenus cat* (Kan^r^, Cm^r^) | This study |

**Table S5:** Antibiotics and drugs used for cultivation of bacteria in this study.

| **Antibiotic** | **Final concentration** | **Use** |
| --- | --- | --- |
| Ampicilin | 100 µg ml^-1^ | Cultivation of XL-1 Blue transformed with *pSG*‑derived plasmids |
| Chloramphenicol | 5  µg ml^-1^ | Cultivation of PY79 strains with integrations at the original gene loci |
| Kanamycin | 10  µg ml^-1^ | Cultivation of 168 deletion mutants and selection of the respective strains of PY79 transformed with chromosomal DNA of 168 deletion mutants |
| Rifampicin | 25  µg ml^-1^ | Cultivation of PY79 *ribH‑mV* strain for comparison with non-drug treated cells |
| Spectinomycin | 50  µg ml^-1^ | Cultivation of strains with ectopic integrations at *amyE* locus |
| Tetracyclin | 20  µg ml^-1^ | Cultivation of XL-1 Blue |

**Table S6:** Plasmids used in this study

| **Plasmid** | **Description / Reference** | **Use and source** |
| --- | --- | --- |
| *pSG1164* | Lewis PJ, Marston AL: GFP vectors for controlled expression and dual labelling of protein fusions in Bacillus subtilis. Gene 1999, 227:101-110. [28] | Construction of *pSG1164‑linker‑mVenus* |
| *pSG1193* | Feucht A, Lewis PJ: Improved plasmid vectors for the production of multiple fluorescent protein fusions in Bacillus subtilis. Gene 2001, 264:289-297. [73] | Construction of *pSG1193‑linker‑mVenus* |
| *pSG1164-linker-mVenus* | Integrative single crossover plasmid for original locus carrying C-terminal coding sequence for linker-mV | This study |
| *pSG1193-linker-mVenus* | Integrative double crossover plasmid for ectopic *amyE* locus integration carrying C‑terminal coding sequence for linker-mV | This study |
| *pSG1164-ribDG-mVenus* | Single crossover plasmid for *ribDG*-mV integration to original locus | This study |
| *pSG1164-ribE-mVenus* | Single crossover plasmid for *ribE*-mV integration to original locus | This study |
| *pSG1164-ribAB-mVenus* | Single crossover plasmid for *ribAB*-mV integration to original locus | This study |
| *pSG1164-ribH-mVenus* | Single crossover plasmid for *ribH*-mV integration to original locus | This study |
| *pSG1193-ribAB-mVenus* | Integrative double crossover plasmid for ectopic *amyE* locus integration of *ribAB‑mV* | This study |
| *pSG1193-ribE-mVenus* | Integrative double crossover plasmid for ectopic *amyE* locus integration of *ribE‑mV* | This study |

**Table S7:** Oligonucleotides used in this study

| Oligonucleotide | 5’-3’ Sequence (Restriction site) | Use |
| --- | --- | --- |
| *Fw linker-mVenus ApaI* | *ATG*GGGCCC*GGTGGAAGTGGAGGTGGATCAGGTG* | Construction of  *pSG1164/1193‑linker‑mVenus* |
| *Rev mVenus SpeI* | *TACACTAGTCTACTTGTAGAGCTCGTCCATTCCAAG* |  |
| *Fw ribDG AvrII* | *GTACCTAGGATGGAAGAGTATTATATGAAGCTGGC* | Construction of  *pSG1164‑ribDG‑mVenus* |
| *Rev ribDG ApaI* | *TACGGGCCCTTCCTTTGTCGGTTTTGCCGTCA* |  |
| *Fw ribE AvrII* | *GTACCTAGGATGGTGACCATGTTTACAGGA* | Construction of  *pSG1164/1193‑ribE‑mVenus* |
| *Rev ribE ApaI* | *TACGGGCCCAAAGCCGTTTTCGCTTAAGAAG* |  |
| *Fw ribAB AvrII* | *GTACCTAGGATGTTTCATCCGATAGAAGAAG* | Construction of  *pSG1164/1193‑ribAB‑mVenus* |
| *Rev ribAB ApaI* | *TACGGGCCCGAAATGAAGTAAATGACCTAGCTTG* |  |
| *Fw ribH ApaI* | *GTACCTAGGATGAATATCATACAAGGAAATTTAG* | Construction of  *pSG1164‑ribH‑mVenus* |
| *Rev ribH AvrII* | *TACGGGCCCTTCAAATGAGCGGTTTAAATTTGCC* |  |
